# A Comprehensive Study on the Chemical Characterization and Neuroprotective Evaluation of Pracaxi Nuts Extracts Obtained by a Sustainable Approach

**DOI:** 10.3390/foods12203879

**Published:** 2023-10-23

**Authors:** Pouya Mohammadnezhad, Alberto Valdés, Ruth E. Barrientos, Elena Ibáñez, Jane Mara Block, Alejandro Cifuentes

**Affiliations:** 1 Foodomics Laboratory, CIAL, CSIC-UAM, Nicolas Cabrera 9, 28049 Madrid, Spain; pouya.mohammadnezhad1990@gmail.com (P.M.); elena.ibanez@csic.es (E.I.); a.cifuentes@csic.es (A.C.); 2 Instituto de Farmacia, Facultad de Ciencias, Universidad Austral de Chile, Valdivia 5090000, Chile; ruth.barrientos@alumnos.uach.cl; 3 Graduate Program in Food Science, Department of Food Science and Technology, Federal University of Santa Catarina, Florianópolis 88034-001, Brazil; janeblock@gmail.com

**Keywords:** green extraction, neuroprotective activity, pracaxi nuts, pressurized liquid extraction, spermidine phenolamides, triterpenoid saponins

## Abstract

The Amazonian *Pentaclethra macroloba* (Willd.) Kuntze nuts contain a lipidic fraction with health-promoting effects, but little is known about the bioactivity of other constituents. In this study, the lipidic fraction obtained using supercritical fluid extraction (SFE) with CO_2_ was chemically characterized by using lipidomics techniques. The SFE-CO_2_ residue, named as pracaxi cake, was re-extracted by pressurized liquid extraction following a biorefinery approach. Using a response surface methodology and based on the extraction yield and different *in vitro* assays, two optimum conditions were obtained: 80% and 12.5% of ethanol at 180 °C. Under these conditions, extraction yield and different *in vitro* measurements related to neuroprotection were assessed. Chemical characterization of these extracts suggested the presence of triterpenoid saponins and spermidine phenolamides, which were not previously reported in pracaxi nuts. These results suggest that pracaxi oil extraction by-products are a valuable source of bioactive compounds with neuroprotective potential.

## 1. Introduction

The Amazon region is the world’s greatest ecosystem and the largest between the six Brazilian biomes. Although this region is characterized by a vast biological variety with high potential for long-term exploitation of new raw material, many plant species remain unknown and/or underexploited [1].

*Pentaclethra macroloba* (Willd.) Kuntze tree is a species native to the Amazon region commonly known as “pracaxi”. It was reported that different chemical constituents in the nuts present healing, anti-inflammatory, larvicidal, or insecticidal activities, while leaves and stem bark extracts possess anti-microbial or anti-haemorrhagic activities, respectively [2]. However, the neuroprotective potential of pracaxi nuts was never reported.

Neurodegenerative disorders are a category of biological diseases characterized by chronic and increasing brain tissue injuries. Alzheimer’s disease (AD), schizophrenia, depression, and Parkinson’s diseases are examples of these illnesses [3]. Recent studies showed that increases in acetylcholinesterase (AChE) and butyrylcholinesterase (BChE) activity levels are the fundamental causes of an AD patient’s increasing memory loss [4]. Oxidative stress caused by the accumulation of reactive oxygen/nitrogen species (ROS/RNS), as well as neuroinflammatory processes linked to the lipoxygenase enzyme (LOX), were also proposed as major causes of neurodegeneration [5,6]. At present, different strategies are followed for trying to prevent or retard AD, such as the search and use of natural constituents present in foods and medicinal plants [7]. These constituents include a myriad of compounds, such as omega-3 fatty acids, fat-soluble vitamins, carotenoids, terpenoids or phenolic acids, among others, which may interfere with different molecular mechanisms related to AD development [8]. Other interesting potential neuroprotective compounds are phenolamides, a family of metabolites that consist of the association of (dihydro) hydroxycinnamic acid derivatives with aliphatic or aromatic amines, that can be found in all plant organs with a predominance in flowers and pollen grains [9]. However, these compounds were never described in pracaxi nuts.

Pracaxi oil obtained from the nuts gained popularity due to its high potential for sustainable exploration [10]. The presence of lipids, carbohydrates, and proteins, as well as secondary compounds, such as triterpene saponins, sterols, or tannins, makes these nuts appropriate for different applications [2]. Edible pracaxi nuts contain 45–48% of oil rich in monounsaturated fatty acids (FAs), oleic acid (47.3–53.5%) being the most abundant, followed by behenic acid (16.1–25.5%), linoleic acid (11.7–13.1%), and lignoceric acid (12.15%) [11,12]. Traditionally, pracaxi oil is extracted by using techniques that require cooking the nuts prior to extraction [13], whereas hydraulic presses are used in the industrial process. However, both methods have low recovery rates and the resulting press cake after the extraction process contains a large amount of oil, a disadvantage that could possibly be overcome by adopting advanced and more effective extraction techniques. Moreover, the resulting press cake can also contain other constituents that can be re-extracted by using green extraction and biorefinery approaches [14]. Supercritical fluid extraction (SFE) and pressurized liquid extraction (PLE) emerged as viable and greener alternatives to traditional extraction processes, and they were demonstrated to be effective in recovering important bioactive compounds from plants, oilseeds, nuts, fruits, and vegetables [15]. In addition, these techniques received great attention due to their advantages, such as the reduction in extraction time, the higher efficiency and selectivity, or the decreased volume of solvent used [16]. SFE is particularly useful for its application on oil-rich materials with promising results [17], and it was successfully applied to obtain oil from pracaxi nuts [11]. On the other hand, PLE proved its efficiency for the extraction of bioactive compounds, such as phenolic compounds, from plants. PLE is characterized by using high temperatures over the boiling point, allowing deeper penetration of the solvent into the sample and higher extraction efficiency. However, this extraction technique was never used to extract bioactive compounds from pracaxi.

In this work, the lipidic fraction of pracaxi nuts was obtained using SFE with CO_2_ (SFE-CO_2_). The oil obtained was chemically characterized by high-pressurized liquid chromatography (HPLC) coupled to a quadrupole-time-of-flight mass spectrometer (Q-TOF MS/MS) and gas chromatography (GC) coupled to a Q-TOF MS instrument. After the lipidic extraction, the defatted residue (pracaxi nuts cake) was re-extracted using an optimized PLE method following a biorefinery approach. The *in vitro* bioactive potential of the optimum extracts was evaluated by measuring the total phenolic content (TPC), the total flavonoid content (TFC), the radical scavenging capacity against ROS and RNS, and the inhibitory capacity against AChE, BChE, and LOX enzymes. Finally, a deep chemical characterization and a comparison between the two optimum extracts was performed by HPLC-Q-TOF MS/MS. The combination of green extraction processes, analytical, and *in vitro* methods is proposed as a powerful strategy for the identification of new compounds, and for the evaluation of their biological activities, that could be associated with potential health benefits.

## 2. Materials and Methods

### 2.1. Samples

Pracaxi nuts (*Pentaclethra macroloba*), from the 2018 harvesting and donated by the company Amazon Oil (Ananindeua, PA, Brazil), were broken with the aid of a stainless-steel hammer, and ground in an IKA A11 mill (IKA, Campinas, SP, Brazil). Then, the powder obtained was sieved through a Tyler mesh n° 14 (average size of 1.19 mm), vacuum packed, and stored at −18 °C for further analysis.

### 2.2. Chemicals and Reagents

Folin-Ciocalteu reagent was obtained from Merck (Darmstadt, Germany). Trizma hydrochloride, AChE enzyme, BChE enzyme, naphthylethylene diamine dihydrochloride, sulphanilamide, acetylthiocholine iodide, linoleic acid, aluminium chloride, phosphoric acid, sodium carbonate, potassium phosphate, monopotassium phosphate, sodium nitroprusside dehydrate, fluorescein, gallic acid (GA), quercetin, galantamine hydrobromide, ascorbic acid, MSTFA (*N*-methyl-*N*-(trimethylsilyl)trifluoroacetamide), TMCS (trimethylchlorosilane), and a 96-well acceptor plate (Catalog no MATRNPS50) were purchased from Sigma-Aldrich (Madrid, Spain). LOX enzyme from *Glycine max* (soybean), 2,2-azobis(2-amidinopropane) dihydrochloride (AAPH), and 4-(amino- 359 sulfonyl)-7-fluoro-2,1,3-benzoxadiazole (ABDF) were obtained from TCI Chemicals (Tokyo, Japan). LC-MS-grade acetonitrile (ACN), LC-MS-grade methanol, and ethanol (EtOH) were obtained from VWR Chemicals (Barcelona, Spain), whereas Milli-Q water was obtained from a Millipore system (Billerica, MA, USA). Formic acid was purchased from Fisher Scientific (Waltham, MA, USA). LC-MS-grade isopropanol, ammonium formate, ammonium acetate, and Val-Tyr-Val were obtained from Sigma-Aldrich (St. Louis, MO, USA). The internal standard 12-[[(cyclohexylamino)-carbonyl]amino]-dodecanoic acid (CUDA) was purchased from LabClinics (Ann Arbor, MI, USA). The lipid standards lysophosphatidylcholine (LPC) 17:0, phosphatidylglycerol (PG) 17:0/17:0, ceramide (Cer) d18:1/17:0, monoacylglycerol (MG) 17:0/0:0/0:0, diacylglycerol (DG) 18:1/2:0/0:0, and triacylglycerol (TG) 17:0/17:1/17:0-d_5_ were provided by Avanti Polar Lipids (Alabaster, AL, USA). The isotope-labelled standard palmitic acid-d_3_ was obtained from Cambridge Isotope Laboratories Inc. (Andover, MA, USA) and fatty acid methyl esters (FAMEs, 400505-51) were obtained from Agilent Technologies (Waldbronn, Germany).

### 2.3. Lipid Extraction of Pracaxi Nuts by Supercritical Fluid Extraction (SFE-CO_2_)

SFE-CO_2_ for lipid extraction of pracaxi nuts was based on the work of Teixeira et al. (2020) [11] and performed using an *in-house* system built in the Foodomics Laboratory at CIAL-CSIC. CO_2_ obtained from a cylinder was cooled and then compressed by a CO_2_ pump from Jasco (Tokyo, Japan). Then, the CO_2_ was preheated and pushed towards the oven, where the extraction cell containing the pracaxi nuts was placed. The flow rate was set to 4 mL/min and extraction time was set at 120 min. Extraction conditions (300 bar and 40 °C) were selected as the optimum conditions as in [11]. Pressure was controlled by adjusting the opening of two needle valves. The extraction cell was filled with a mixture of 2 g of sample and 4 g of sea sand placed between two layers of glass wool. The extracts obtained were collected after CO_2_ expansion (and subsequent cooling) and protected from light by aluminium foil. These experiments were performed in triplicate. Each extract was then transferred to a previously weighed glass vial and evaporated by a gentle nitrogen stream to calculate the extraction yield. The chemical characterization was performed on the pool of extracts obtained after the three extractions.

### 2.4. Experimental Design for Extraction of Bioactive Compounds from Pracaxi Nuts Cake by Pressurized Liquid Extraction (PLE)

After the lipid extraction using SFE-CO_2_, the obtained pracaxi nuts cake was re-extracted by PLE using an accelerated solvent extractor Dionex model ASE 200 (Sunnyvale, CA, USA) equipped with a solvent controller. Briefly, 1 g of pracaxi cake (containing sand from the SFE extraction step) was placed into an 11 mL extraction cell and the extraction was conducted during 20 min using the following parameters: 100 bar, heat-up time 5 min; flush volume, 60%; and purge with N_2_ for 90 s. The experiments were carried out following a central composite design (CCD) with two factors considered at three levels: solvent composition (100% Milli-Q water, 100% EtOH, and 50/50 (*v*/*v*) EtOH/Milli-Q water) and temperature (50, 115, and 180 °C), including four replicates at the central point. This CCD was used to optimize the extraction conditions using a response surface methodology (RSM). The obtained extracts were dried under nitrogen flow, and the extraction yield, TPC, AChE enzymatic inhibition activity, and ROS scavenging capacity were evaluated as response variables by Statgraphics Centurion XVI (v.16.1.11) software (StatPoint Technologies, Inc., Warrenton, VA, USA). Analysis of variance (ANOVA), coefficient of determination (R^2^) of response surfaces, *p* values, standardized Pareto charts, interaction plot, and lack-of-fit testing for the extraction conditions were obtained, accepting significance at *p* < 0.05 (see Tables S1–S4 and Figures S1 and S2, Supplementary Materials). Based on these results, two optimum extraction conditions were selected: 80/20 (*v*/*v*) EtOH/Milli-Q water and 180 °C (PLE80), and 12.5/87.5 (*v*/*v*) EtOH/Milli-Q water and 180 °C (PLE12.5).

### 2.5. Chemical Characterization of Lipids Obtained by SFE-CO_2_ from Pracaxi Nuts

#### 2.5.1. HPLC-CSH-Q-TOF MS/MS Analysis

For the lipid profiling, the pracaxi nut oil obtained by SFE-CO_2_ was diluted to 1 mg/mL in methanol containing an internal standard mixture of LPC (17:0), PG (17:0/17:0), Cer (d18:1/17:0), MG (17:0/0:0/0:0), DG (18:1/2:0/0:0), TG (17:0/17:1/17:0)-d_5_, palmitic acid-d_3_, and CUDA. A volume of 3 μL (for electrospray ionization—ESI positive) and 5 μL (for ESI negative) were injected into a HPLC model 1290 (Agilent Technologies), and compounds were separated using a Waters Acquity CSH C18 column (100 mm length × 2.1 mm id; 1.7 μm particle size) equipped with a Waters Acquity VanGuard CSH C18 pre-column (5 mm × 2.1 mm id; 1.7 μm particle size) as previously described [18]. Compounds were eluted into a Q-TOF series 6540 from Agilent Technologies, and equipped with an Agilent Jet Stream thermal orthogonal ESI source. For proper mass accuracy, spectra were corrected using ions *m*/*z* 121.0509 (C_5_H_4_N_4_) and 922.0098 (C_18_H_18_O_6_N_3_P_3_F_24_) in ESI positive mode, and *m*/*z* 119.0363 (C_5_H_4_N_4_) and 980.0164 (C_18_H_18_O_6_N_3_P_3_F_24_ + acetate) in ESI negative mode, simultaneously pumped into the ionization source. A blank sample including only the internal standards was added for blank subtraction. LC-MS/MS raw data files were converted to Abf format using Abf Converter (v.4.0.0) software, and data processing was conducted using MS-DIAL (v.4.8) software [18] as previously described [19]. Lipid annotation was carried out by using an *in-house* retention time (RT)-*m*/*z* library and the MSP (LipidBlast, version 68) included in MS-DIAL. Lipids were annotated following the Metabolomics Standard Initiative (MSI) guidelines: MSI level 1 for metabolites with precursor *m*/*z*, *in-house* RT-*m*/*z* library, and MS/MS spectral library matching; MSI level 2a for metabolites with precursor *m*/*z* and *in-house* RT-*m*/*z* library matching, and MSI level 2b for metabolites with precursor *m*/*z* and MS/MS spectral library matching. Peak area calculation was performed by combining the area of the different adducts detected for the same compound.

#### 2.5.2. GC-Q-TOF MS Analysis

For GC-MS analyses, 1 mg of pracaxi nuts oil obtained by SFE-CO_2_ and a blank sample were derivatized by adding 10 μL of methoxyamine hydrochloride in pyridine (40 mg/mL) and shaking the samples for 90 min at 30 °C. Then, the SFE extract, the blank sample, and a mixture of FAMEs were trimethylsilylated by adding 90 μL of MSTFA/1% TMCS and incubated at 37 °C for 30 min. Finally, aliquots of 1 μL of the samples were injected in splitless mode and analyzed using an Agilent 7890 GC coupled to an Agilent 7200 Q-TOF MS (Agilent Technologies), equipped with an Agilent 30 m long, 0.25 mm id DB-5MS column (0.25 μm film thickness). The chromatographic gradient started at 60 °C (1 min), 10 °C/min to 325 °C, and was held for 10 min using a constant flow of 1 mL/min. Mass spectrometry data were collected using 750 MCP detector voltage at *m*/*z* 20–600 with 5 spectra/s, electron ionization at −70 eV, and an ion source temperature of 250 °C. GC-MS raw data files were converted to ABF format and processed with MS-DIAL (v.4.8) software. Retention index using FAMEs was used with the following parameters: retention index tolerance for MSP library identification, 3000; EI similarity cut off, 70%; and identification score cut off and similarity tolerance, 70%. The MSP file used for annotation was a combination of NIST17, MassBank of North America (https://mona.fiehnlab.ucdavis.edu/spectra/browse?query=tags.text%3D%3D%22GC-MS%22, accessed on 1 July 2023), and the Fiehn BinBase DB, Rtx5-Sil MS, and FAMEs RI (http://prime.psc.riken.jp/compms/msdial/main.html#MSP, accessed on 1 July 2023).

Compounds identified by HPLC-CSH-Q-TOF MS/MS and GC-Q-TOF MS were classified in different subclasses by using the “ClassyFire” tool from https://cfb.fiehnlab.ucdavis.edu/ (accessed on 1 August 2023).

### 2.6. Extraction Yield, Total Phenolic Content, and Total Flavonoid Content

The extraction yield was expressed as the percentage of the extract mass in the dry basis and the mass of initial pracaxi nuts fed into the SFE-CO_2_ or the PLE extraction cell. TPC and TFC of the extracts obtained by PLE from pracaxi nuts cake were assessed according to previously published methods [20,21,22]. For TPC measurement, the calibration curve was established using 0.031–2 mg GA/mL in EtOH, and it was used to calculate the TPC of the PLE extracts expressed as milligrams of GA equivalents per gram of extract (mg GAE/g extract). For TFC, the results are expressed as milligrams of quercetin equivalents (QE) per gram of extract (mg QE/g extract). All measurements were performed in triplicate.

### 2.7. ROS/RNS Scavenging Capacity, AChE/BChE, and LOX Inhibitory Activity in the Extracts Obtained by PLE from Pracaxi Nuts Cake

The ROS scavenging capacity of the PLE extracts obtained from pracaxi nuts cake was measured using the oxygen radical absorbance capacity (ORAC) assay previously described [23,24]. Ascorbic acid and 10% EtOH (*v*/*v*) were used as reference standard and blank control solutions, respectively. Complementary, the RNS scavenging capacity was estimated referring to the nitric oxide (NO) radical scavenging assay [24,25]. Ascorbic acid was used as the reference standard and 25% EtOH was used as blank control solution. The AChE and BChE inhibitory activities of pracaxi nuts cake PLE extracts were estimated according to the fluorescent enzyme kinetic method described by Sanchez-Martinez et al. [24]. Galantamine hydrobromide was used as the reference inhibitor, and 50% EtOH was used as blank control. Finally, the LOX inhibitory activity of pracaxi nuts cake PLE extracts was determined as described in [26], with slight modifications [24]. Quercetin was used as a reference inhibitory and 25% EtOH was used as a blank control.

### 2.8. Chemical Characterization of Pracaxi Nuts Cake PLE Extracts Using HPLC-C18-Q-TOF MS/MS

The optimum PLE80 and PLE12.5 extracts (Section 2.4) were dissolved in EtOH to a final concentration of 3 mg/mL. Then, samples were vortexed for 30 s, centrifuged at 14,800 rpm for 5 min at 4 °C and the supernatants were collected and stored at −80 °C until analysis. Aliquots of 2 μL were injected into the same HPLC-Q-TOF MS/MS instrument as specified above. Compounds were separated using an Eclipe Plus C18 analytical column (100 mm × 2.1 mm, particle size 1.8 μm) and a C18 guard column (0.5 cm × 2.1 mm, particle size 1.8 μm), both from Agilent. Milli-Q water was used as mobile phase (A) and ACN as mobile phase (B), and 0.1% formic acid was used as a mobile phase modifier. The column temperature was held at 40 °C and the flow rate was set to 0.5 mL/min, with the following gradient: 0–30% B in 7 min; 30–80% B in 9 min; 80–100% B in 11 min; and 100% B for 14 min. The mass spectrometer was operated in ESI positive and ESI negative modes, using the following parameters: capillary voltage of 3000 V for ESI positive and −3000 V for ESI negative; mass range from 25 to 1100 *m*/*z*; nebulizer pressure of 40 psig; and drying gas flow rate of 8 L/min and 300 °C. The sheath gas flow was 11 L/min at 350 °C. MS/MS analyses were performed employing the auto MS/MS mode using five precursors per cycle, dynamic exclusion after two spectra (released after 0.5 min), and collision energies of 20 and 40 V. Mass accuracy was corrected as explained above. Data processing was performed using MS-DIAL (v.4.8) software, and MS/MS spectra from NIST20, LipidBLAST, and the MoNA databases were used for the tentative identification of compounds **1**–**42**, **100**–**104**, and **106**–**114**. Moreover, the most abundant compounds annotated as “unknowns” by MS-DIAL (v.4.8) software were individually inspected and tentatively identified by combining SIRIUS 4 software [27], the manual interpretation of their acquired MS/MS spectra, and taking into consideration their retention times. Peak areas were normalized by the total sum of identified peaks in each sample. Principal component analysis (PCA), partial least squares discriminant analysis (PLS-DA), and two-sample *t*-test were performed with the MetaboAnalyst 5.0 website tool (https://www.metaboanalyst.ca, accessed on 1 August 2023), and differences between metabolites were considered significant when *p* < 0.05.

## 3. Results and Discussion

### 3.1. Extraction Yield and Chemical Characterization of the Lipids Obtained by SFE-CO_2_ from Pracaxi Nuts

**Figure 1:**
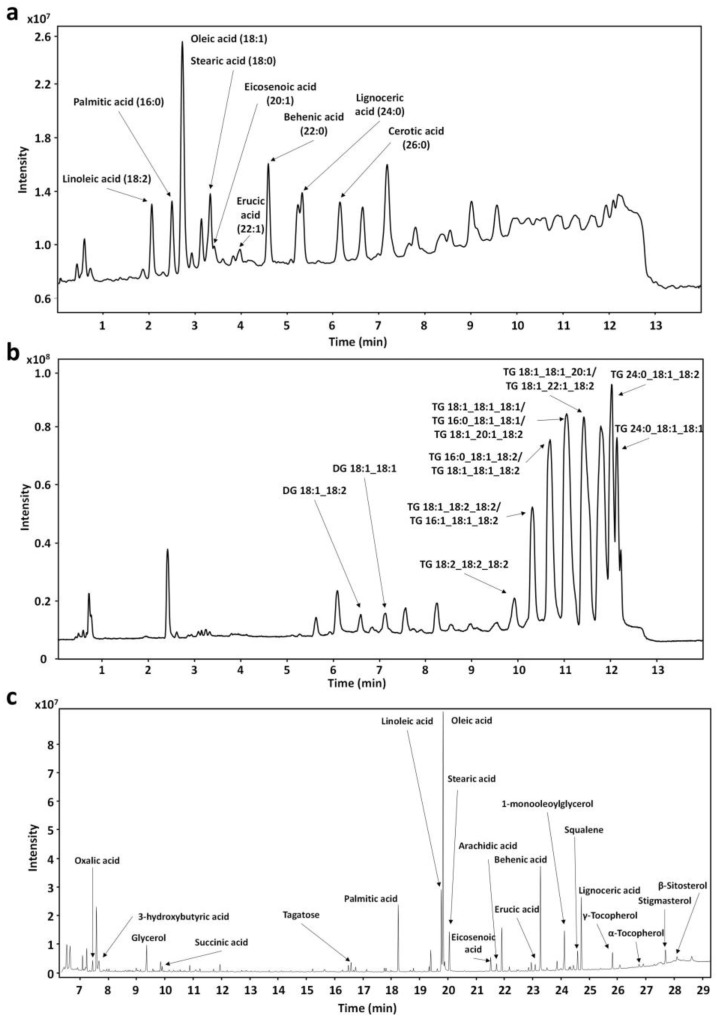
Total ion current (TIC) chromatograms with the most abundant annotated compounds analyzed by HPLC-CSH-Q-TOF MS/MS in ESI negative ion mode (**a**), ESI positive ion mode (**b**), and GC-Q-TOF MS (**c**).

In the present work, we selected the extraction conditions previously optimized by Teixeira et al. (2020) for the same raw material [11]. In order to fix the same S/F ratio (solvent-to-feed), extraction time was fixed at 120 min in the present work. Under these conditions, the extraction yield obtained for the lipidic fraction was 29.3 ± 2.5%, which is lower than previous results (42.0 ± 3.4%) [11]. This can be due to the different configuration of the system employed in both works. The analysis performed by HPLC-CSH-Q-TOF MS/MS resulted in the annotation of 30 compounds in ESI (−) and 97 in ESI (+) modes, giving a total of 127 compounds annotated in the lipid extract of pracaxi nuts. The main annotated compounds in ESI (−) were free fatty acids (FA), with 29 different species. The most abundant FA identified was oleic acid (C18:1), followed by behenic acid (C22:0), linoleic acid (C18:2), lignoceric acid (C24:0), stearic acid (C18:0), and palmitic acid (C16:0) (Figure 1a). These results are in agreement with previous studies that demonstrated that pracaxi oil is rich in oleic, behenic, linoleic, lignoceric, stearic, and palmitic acids [11,12]. In addition, it was possible to detect the presence of other less abundant lipids, such as eicosenoic (C20:1), erucic (C22:1), arachidic (C20:0), cerotic (C26:0), nervonic (C24:1), or palmitoleic (C16:1) acids (Table S5, Supplementary Materials). TGs were the main compounds annotated in ESI (+), corresponding to 66 different species. They were followed by DGs with 17 species and oxidized TGs with 10 species (Table S6, Supplementary Materials). Figure 1b shows that the most abundant peaks correspond to TGs, such as 54:3|18:1_18:1_18:1, 58:3|22:0_18:1_18:2, 58:4|18:1_22:1_18:2, 56:3|18:1_18:1_20:1, or 54:5|18:1_18:2_18:2. As expected, these TGs were composed of the main FAs identified in ESI (−) mode (oleic, behenic, and linoleic acids). DGs (36:2|18:1_18:1 or 36:3|18:1_18:2) and the oxidized TGs (58:2;1O|22:0_18:1_18:1;1O or 54:3;1O|18:1_18:1_18:1;1O) were mainly composed also of these FAs. TGs composition of pracaxi oil with the same FAs was previously reported [11]. In addition, with the results obtained in the present study, it was possible to expand the chemical characterization of this complex matrix since a greater number of TGs (66), DGs (17), and oxidized TGs (10) were identified. Complementarily, the GC-Q-TOF MS analysis resulted in the annotation of 110 compounds, FAs and conjugates (16%), carbohydrates and carbohydrate conjugates (15%), and FAs esters (6%) being the most represented subclasses (Table S7, Supplementary Materials). The most abundant FAs were oleic, palmitic, and stearic acids, followed by glycerol, 1-monooleoylglycerol, and lignoceric acid (Figure 1c). These results confirm what was determined by CSH-Q-TOF MS/MS. Dicarboxylic acids and derivatives (oxalic acid, methylmalonic acid, succinic acid, glutaconic acid, and malonic acid); three quinone and hydroquinone lipids (γ, α, and δ-tocopherol); two stigmastanes and derivatives (stigmasterol and β-sitosterol); one pyrimidine nucleoside (uridine); one pyrrolidone (2-pyrrolidinone); and one triterpenoid (squalene), were also identified. These results also agree well with previous publications that demonstrate that pracaxi nuts contain sterols, such as stigmasterol and β-sitosterol [28]; and tocopherols [29]. In addition, the high amounts for oxalic acid detected in pracaxi nuts were also reported for other nuts, such as almonds or Brazilian nuts [30,31].

### 3.2. Optimization of Bioactive Compounds Extraction Conditions from Pracaxi Cake by PLE

The PLE design selected was a CCD, considering the optimization of factors that improve the antioxidant and neuroprotective activities of interest that could be found in pracaxi cake. This design was used to further attain the best of the selected factors, i.e., temperature (°C) and water/EtOH ratio (%), to obtain the highest yield and the highest antioxidant, TPC, and neuroprotective activities (antioxidant, ROS, and AChE inhibitory activity) from pracaxi residue. Following this, new response variables related to bioactive content (TFC) and neuroprotective activities (such as radical scavenging capacity against RNS, and the inhibitory capacity against BChE and LOX enzymes) were tested using the optimum extracts. Table 1 shows the results of the experimental design, including the response variables employed for the optimization of bioactives extraction from pracaxi cake.

Results show that the extraction yield was significantly higher at high temperatures. These results are expected because an increase in temperature increases the solubility of compounds and reduces the solvent viscosity, enhancing the mass transfer from the sample to the extraction solvent, as previously reported [32,33]. The highest extraction yield (22.8%) was achieved when 100% EtOH at 180 °C was used. On the other hand, the lowest yield (3.5%) was obtained with 100% water at 50 °C. The temperature was also an important factor for the extraction and activity of the bioactive compounds. The highest values for TPC (176.5, 167.4, and 163.8 mg GAE/g) were obtained with 100% water, 50% EtOH, and 100% EtOH at 180 °C, respectively. Otherwise, the lowest values were obtained with 100% water (75.0 mg GAE/g) and 50% EtOH (99.7 mg GAE/g) at 50 °C. The half maximal inhibitory concentration (IC_50_) values for ROS and AChE assays decreased as the temperature increased, but the best results for these assays were obtained at different conditions. The best result for ROS was obtained with 100% water at 180 °C (IC_50_ of 1.98 μg/mL). On the other hand, the best value for AChE was obtained with 50% EtOH at 180 °C (IC_50_ of 247 μg/mL). Since ROS and AChE results are expressed as IC_50_ (μg/mL), it means that higher activities are achieved when lower IC_50_ values are obtained.

The optimum PLE conditions were calculated considering the extraction yield, TPC, ROS, and AChE values as response variables using RSM. In addition, RSM was also performed excluding the extraction yield since previous studies reported that the yield is not always necessarily related to the neuroprotective potential of natural extracts [34]. The optimum extraction conditions were obtained with 80% EtOH at 180 °C (PLE80) when the extraction yield was included. On the other hand, the optimum conditions were 12.5% EtOH at 180 °C (PLE12.5) when the extraction yield was not included in the response variables (Table S8, Supplementary Materials). From the Pareto charts of each model (Figure S1, Supplementary Materials), it can be seen that temperature significantly affects all the response variables, while the solvent composition affects all variables except TPC. It is also clear that the temperature was an important variable in both experimental designs since it was the same (180 °C) for PLE80 and PLE12.5 (Figure S2, Supplementary Materials).

### 3.3. Comparison between the Two PLE Optimum Conditions

Three independent experiments were performed for each optimum to experimentally confirm the predicted values for TPC, ROS, and AChE. In addition, four more assays were included (TFC, RNS, BChE, and LOX) to obtain more information on the neuroprotective, anti-inflammatory, and antioxidant potential of these extracts (Table 2).

The extraction yield for PLE80 (24.03%) was significantly higher than that obtained for PLE12.5 (15.9%). This was expected since the optimum condition was calculated including the extraction yield as a response variable for obtaining PLE80. In addition, the extraction yield for PLE80 extract was better than the one predicted by RSM (Table S8, Supplementary Materials). The extraction yield observed for PLE12.5 was similar to that obtained with 50% EtOH and 100% water at 180 °C (see Table 1). The difference for a TPC value between PLE80 (91.9 mg GAE/g) and PLE12.5 (103.9 mg GAE/g) was not significantly different. However, the TPC results are lower than those predicted by RSM (Table S8, Supplementary Materials). On the other hand, the TFC value obtained for PLE12.5 (7.0 mg QE/g) was significantly higher than that for PLE80 (6.6 mg QE/g). These results indicate that some of the flavonoid and phenolic compounds present in pracaxi nuts are relatively polar, as they were slightly better extracted when more water was included during the extraction. It is also interesting that the TPC and TFC values are not correlated to the extraction yield, suggesting that other compounds different from phenolics and flavonoids are being extracted when using a higher EtOH percentage. Furthermore, the TPC (2.66 mg GAE/g) and TFC (0.11 mg of rutin equivalents/g), reported for pracaxi cake extracted by percolation with 70% EtOH [35,36], indicate that PLE is a more efficient method for obtaining phenolic and flavonoid compounds from this matrix.

The IC_50_ values for ROS and RNS scavenging capacity obtained for both extracts were similar (Table 2). In addition, the values obtained for ROS (IC_50_ of 1.5 μg/mL for PLE80; 1.6 μg/mL for PLE12.5) were better in both optimum conditions than those predicted by RSM (Table S8, Supplementary Materials). The ROS results complement previous studies showing the antioxidant potential of pracaxi oil [11] and pracaxi cake [35], highlighting the potential of pracaxi co-products as a good source of antioxidant compounds.

Moreover, the AChE inhibitory capacity for the extract PLE80 was slightly higher than that obtained for the extract PLE12.5, and it was significantly higher for the BChE inhibitory capacity (Table 2). Both extracts exhibited a moderate anti-cholinergic activity compared to galantamine, the reference inhibitor used in this study. The results for AChE inhibitory capacity were expected as the best value obtained during the PLE optimization achieved when using 50% EtOH and 180 °C, but the results were slightly better when 100% EtOH was compared to 100% water (Table 1). However, these results are lower than those predicted by RSM (Table S8, Supplementary Materials). Moreover, the AChE results correlate well with those for BChE inhibitory activity, but they are not correlated with the TPC or TFC content, suggesting that other compounds different than phenolics and flavonoids could also be responsible for the ChE inhibitory potential.

Finally, the IC_50_ value for the LOX inhibitory capacity was lower for PLE12.5 (14.9 μg/mL) than for PLE80 (18.6 μg/mL) extracts. Both results are close to the refence LOX inhibitor (quercetin) used in this study (12.2 μg/mL). Nobre Lamarão et al. (2023) summarized the anti-inflammatory capacity of pracaxi oil, but this activity for pracaxi cake was not found in the literature [2]. These values are also correlated with the slightly higher TPC and TFC content observed for PLE12.5, and previous studies demonstrated the anti-inflammatory properties of different phenolic [37] and flavonoid [38] compounds. These authors indicated that the presence of hydroxyl groups in these molecules are related with their anti-inflammatory activity.

### 3.4. Chemical Characterization of Extracts from Pracaxi Nuts Cake Obtained by PLE

**Figure 2:**
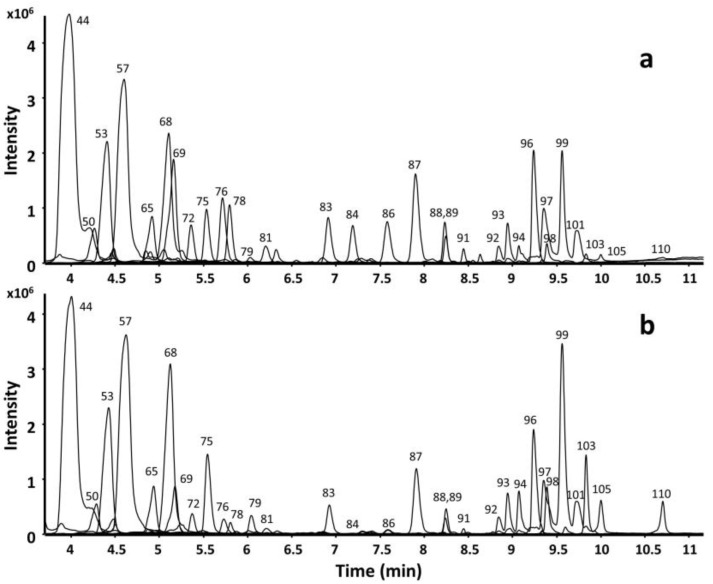
Representative extracted ion chromatograms (EICs) of the most abundant spermidine phenolamides and triterpenoid saponins tentatively identified in pracaxi cake PLE80 (**a**) and PLE12.5 (**b**) extracts, after HPLC-C18-Q-TOF MS/MS ESI (+) analyses. The tentative compound name corresponding to each number can be found in Table 3 and Table S9.

The chemical characterization of PLE80 and PLE12.5 pracaxi nuts cake extracts is presented in Figure 2 and Table 3. The analysis performed in ESI (+) and ESI (−) mode allowed the tentative identification of 99 and 68 compounds, respectively. Among the 114 tentative identified compounds in both ionization modes, 49 spermidine phenolamides and 12 triterpenoid saponins (hederagenin, oleanolic acid, and their respective glucosides) were the main compounds (Table 3). The tentative metabolite name, proposed molecular formula, retention time, exact mass, adduct type, MS/MS spectra, and peak area of these compounds are shown in Table S9, Supplementary Materials.

Among the different tentative identified compounds (Table 3), compounds **43**–**91** were identified as spermidine phenolamides, which occur in vegetables mainly as hydroxycinnamic acids and derivatives covalently linked through amide bonds to an aliphatic polyamine [9]. Previous studies reported the characteristic MS/MS fragmentation patterns of several (dihydro) hydroxycinnamic acids and their amide derivatives [39,40]. These authors reported that hydroxycinnamic acids containing coumaric acid are identified in MS analyses mainly by a neutral loss of 146.04 (–C_9_H_6_O_2_) and for having a characteristic fragment ion in ESI (+) at *m*/*z* 147.04. In addition, a neutral loss of 162.03 (–C_9_H_6_O_3_) and a fragment ion in ESI (+) at *m*/*z* 163.04 indicate the presence of caffeic acid in these compounds. Additionally, a neutral loss of 176.05 (–C_10_H_8_O_3_) and a fragment ion in ESI (+) at *m*/*z* 177.05 are related to ferulic acid. On the other hand, dihydrohydroxycinnamic acids containing dihydrocoumaric acid are identified by neutral losses of 148.05 (–C_9_H_8_O_2_) and 106.04 (–C_7_H_6_O); dihydrocaffeic acid is identified by neutral losses of 164.04 (–C_9_H_8_O_3_) and 122.04 (C_7_H_6_O_2_), and for having a characteristic fragment ion in ESI (+) at *m*/*z* 165.05; and dihydroferulic acid is identified by neutral losses of 178.06 (–C_10_H_10_O_3_) and 136.05 (–C_8_H_8_O_2_), and for having a characteristic fragment ion in ESI (+) at *m*/*z* 179.07. Moreover, hydroxycinnamic acid amide glucoside derivatives have similar characteristic fragmentation patterns to their corresponding hydroxycinnamic acid amides, in addition to the neutral loss of the corresponding sugar moiety, such as 162.05 (–C_6_H_10_O_5_), 132.04 (–C_5_H_8_O_4_), or 146.06 (–C_6_H_10_O_4_).

**Figure 3:**
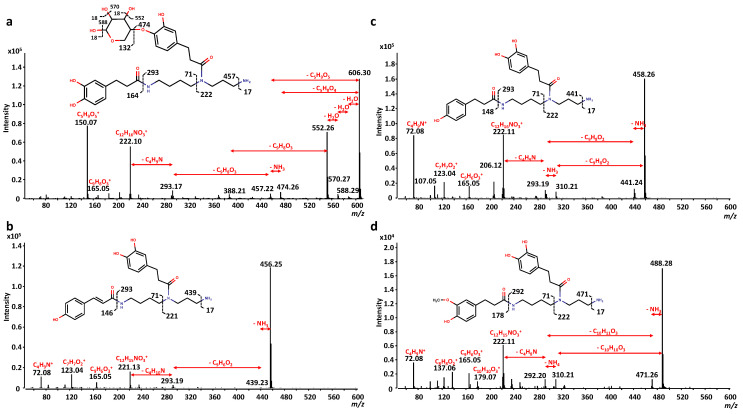
MS/MS fragmentation patterns and tentative structures with key fragments assigned for different spermidine phenolamides: (**a**) compound **44**; (**b**) compound **57**; (**c**) compound **87**; and (**d**) compound **50**.

Based on the previous information, compounds **44** and **47** were identified as *N*-*N*’-bis-(dihydrocaffeoyl) spermidine isomers, with [M + H]^+^ ions at *m*/*z* 474.26, and fragment ions at *m*/*z* 457.23 [M + H-NH_3_]^+^, 310.21 [M + H-dihydrocaffeoyl]^+^, 293.19 [M + H-dihydrocaffeoyl-NH_3_]^+^, 222.11, 165.05 [dihydrocaffeoyl]^+^, 123.04, and 72.08 in ESI (+). Moreover, compound **44** had a [M−H]^−^ ion at *m*/*z* 472.24, with fragment ions at *m*/*z* 350.21 [M-H-122]^−^, 308.20 [M-H-dihydrocaffeoyl]^−^, 186.16 [M-H-dihydrocaffeoyl-122]^−^, and 121.03 (Figure 3a). Based on a neutral loss that yields a *m*/*z* at 457.23 (NH_3_ loss) as a first fragment and a subsequent hydrogen rearrangement in ESI (+) mode, we propose a non-linear configuration (N^1^–N^5^– or N^5^–N^10^–) of compounds **44** and **47**. In addition, the MS/MS spectra of *N*-*N*’-bis-(dihydrocaffeoyl) spermidine were previously reported [41], which perfectly matched those obtained in the present work. Based on the similar MS/MS fragmentation pattern as compounds **44** and **47**, compounds **46**, **52**, **54**, **56**, **57**, **59**–**64**, **67**–**75**, **77**, and **80** were tentatively identified as *N*-*N*’-bis-(dihydrocaffeoyl) spermidine conjugates, with a non-linear configuration (N^1^–N^5^– or N^5^–N^10^–). All these compounds present at least two characteristic ions from *N*-*N*’-bis-(dihydrocaffeoyl) spermidine (474.26, 457.22, 222.11, and/or 165.05 *m*/*z*) in ESI (+), and most of them had neutral losses of 164.05 (dihydrocaffeoyl, -C_9_H_8_O_3_) and 122.04 (–C_7_H_6_O_2_) in ESI (−) (see Table S9, Supplementary Materials). A proposed MS/MS interpretation for compound **57** in ESI (+) is shown as an example in Figure 3b. A non-linear configuration (N^1^–N^5^– or N^5^–N^10^–) is proposed based on a neutral loss of 95.04 (–C_5_H_5_NO) as a first fragment (instead of a NH_3_ loss), with a subsequent dihydrocaffeoyl (–C_9_H_8_O_3_) loss. The same behaviour was observed for the other compounds.

Moreover, compounds **76**, **78**, **81**–**90**, and **92** were also tentatively identified as *N*-*N***′**-bis-(dihydrocaffeoyl) spermidine conjugates, as they exhibit a fragment ion at *m*/*z* 222.11 in ESI (+), neutral losses of 164.05 and 122.04 in ESI (−), and some of them present a fragment ion at *m*/*z* 165.05 in ESI (+), all characteristics of *N*-*N***′**-bis-(dihydrocaffeoyl) spermidine derivatives. However, a N^1^-N^10^-configuration for these compounds is proposed. This inference is based on the absence of a neutral loss in ESI (+) that yields an *m*/*z* at 474.26 or 457.22 (NH_3_ loss for compound **44**; and C_5_H_5_NO loss for compound **57**, respectively) as a first fragment. In a N^1^–N^10^ configuration, all nitrogen atoms are in internal positions and their loss as a first fragment would require a molecular reorganization, resulting in novel linkage types. Since this is not a common event in MS, a linear structure should be considered, where both dihydrocaffeic acid molecules are linked to the terminal nitrogen atoms of the spermidine skeleton, and a radical (R1) is attached to the central nitrogen. A proposed MS/MS interpretation in ESI (+) of compound **87** is shown in Figure 3c. Complementary to the differences in the MS/MS fragmentation patterns, the retention time of these compounds also indicates the proposed configuration, as the linear (N^1^–N^10^–) configuration confers a higher lipophilicity than the non-linear (N^1^–N^5^– or N^5^–N^10^–) configurations. Some common patterns were also observed for compounds **76**, **78**, **81**, **82**, **84**, **86**, and **88**. They have a dihydrocaffeoyl loss (–C_9_H_8_O_3_) as the first fragment in ESI (+). In addition, compounds **83**, **84**, **86**–**89**, and **91** formed abundant [M + Na]^+^ and [M + K]^+^ adducts in ESI (+); and [M + Cl]^−^ and [M-H + HNO_3_]^−^ adducts in ESI (−). These results suggest that they are chemically related.

Furthermore, compounds **50** (Figure 3d) and **55** were tentatively identified as *N*-caffeoyl-*N*’-dihydrocaffeoyl spermidine isomers. Compound **50** exhibited a [M + H]^+^ ion at *m*/*z* 472.24 with fragment ions at *m*/*z* 457.23 [M + H-NH]^+^, 310.21 [M + H-caffeoyl]^+^, 293.19 [M + H-caffeoyl-NH_3_]^+^, 222.11, 165.05 [dihydrocaffeoyl]^+^, 163.04 [caffeoyl]^+^, 123.04, and 72.08. Similarly, compound **55** exhibited an [M + H]^+^ ion at *m*/*z* 472.24, but the fragment ions were slightly different (*m*/*z* at 455.22 [M + H-NH_3_]^+^, 310.21 [M + H-caffeoyl]^+^, 293.18 [M + H-caffeoyl-NH_3_]^+^, 239.14, 222.11, 220.10, 163.04 [caffeoyl]^+^, and 123.04 and 72.08). This result indicates some differences in their chemical configuration. Complementary, these compounds have similar [M-H]^−^ ions at *m*/*z* 470.23, producing major fragment ions at *m*/*z* 350.21 [M-H-120]^−^, 308.20 [M-H-caffeoyl]^−^, 186.16 [M-H-caffeoyl-122]^−^, and 161.02 [caffeoyl]^−^. Based on the MS/MS fragmentation similarities, compound **49** was tentatively identified as *N*-caffeoyl-*N*’-dihydrocaffeoyl spermidine conjugate (+C_5_H_2_O). This compound presents two characteristic fragment ions of dihydrocaffeoyl spermidine in ESI (+) (*m*/*z* at 222.11 and 165.05), and an ion at *m*/*z* 455.22 instead of 457.22, suggesting the presence of a caffeoyl group instead of a second dihydrocaffeoyl group.

**Figure 4:**
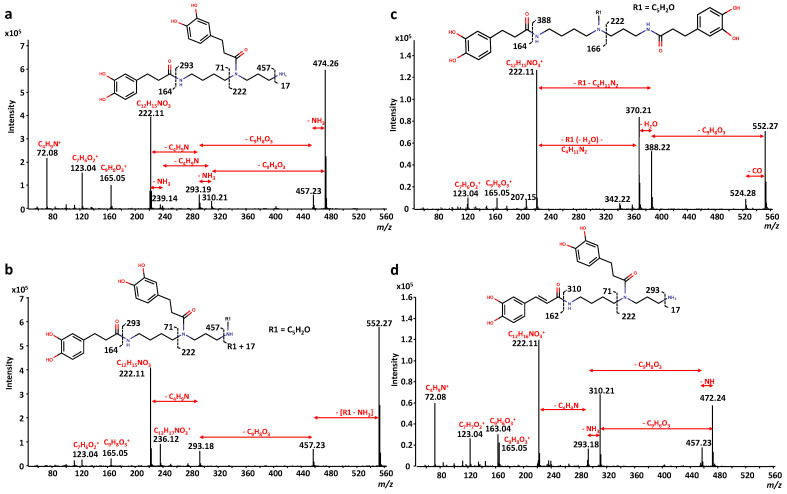
MS/MS fragmentation patterns and tentative structures with key fragments assigned for different spermidine phenolamides: (**a**) compound **45**; (**b**) compound **48**; (**c**) compound **53**; and (**d**) compound **58**.

Based on previous information [40], we tentatively identified compounds **43**, **45**, and **51** as *N*-*N*’-bis-(dihydrocaffeoyl) spermidine monoglucosides (+C_6_H_10_O_5_, +C_5_H_8_O_4_, and +C_6_H_10_O_4_, respectively). Compound **43** exhibited a [M + H]^+^ ion at *m*/*z* 636.31, having fragment ions at *m*/*z* 618.33 [M + H-H_2_O]^+^, 600.30 [M + H-H_2_O-H_2_O]^+^, 474.26 [M + H-C_6_H_10_O_5_]^+^, 457.23 [M + H-C_6_H_10_O_5_-NH_3_]^+^, 384.17, 293.19 [M + H-C_6_H_10_O_5_-NH_3_-dihydrocaffeoyl]^+^, 222.11, 112.11, and 72.08. In addition, the [M-H]^−^ ion at *m*/*z* 634.30 has mainly fragment ions at *m*/*z* 616.28 [M-H-H_2_O]^−^ and 472.24 [M + H-C_6_H_10_O_5_]^−^. Compound **45** (Figure 4a) exhibited a [M + H]^+^ ion at *m*/*z* 606.30, having major fragment ions at *m*/*z* 588.29 [M + H-H_2_O]^+^, 570.28 [M + H-H_2_O-H_2_O]^+^, 552.26 [M + H-H_2_O-H_2_O-H_2_O]^+^, 474.26 [M + H-C_5_H_8_O_4_]^+^, 457.22 [M + H-C_5_H_8_O_4_-NH_3_]^+^, 293.17 [M + H-C_5_H_8_O_4_-NH_3_-dihydrocaffeoyl]^+^, 222.10, and 150.07. Moreover, the [M-H]^−^ ion at *m*/*z* 604.29 has five fragment ions at *m*/*z* 586.28 [M-H-H_2_O]^−^, 568.26 [M-H-H_2_O-H_2_O]^−^, 545.27, 472.24 [M-H-C_5_H_8_O_4_]^−^, and 350.21 [M-H-C_5_H_8_O_4_-122]^−^. On the other hand, compound **51** exhibited a [M + H]^+^ ion at *m*/*z* 620.32, and fragment ions at *m*/*z* 602.31 [M + H-H_2_O]^+^, 584.29 [M + H-H_2_O-H_2_O]^+^, 566.28 [M + H-H_2_O-H_2_O-H_2_O]^+^, 474.26 [M + H-C_6_H_10_O_4_]^+^, 457.23 [M + H-C_6_H_10_O_4_-NH_3_]^+^, 293.18 [M + H-C_6_H_10_O_4_-NH_3_-dihydrocaffeoyl]^+^, 222.11, and 164.11.

Compound **48** (Figure 4b) was identified as *N*-coumaroyl-*N*’-dihydrocaffeoyl spermidine, as it exhibited a [M + H]^+^ ion at *m*/*z* 456.25, having fragment ions at *m*/*z* 439.23 [M + H-NH_3_]^+^, 293.19 [M + H-NH_3_-coumaroyl]^+^, 221.13, 165.05 [dihydrocaffeoyl]^+^, 123.04, and 72.08; and a [M-H]^−^ ion at *m*/*z* 454.23, with fragment ions at *m*/*z* 332.20 [M-H-122]^−^, 297.15, 233.96, and 192.02.

Compound **53** (Figure 4c) was tentatively identified as *N*-dihydrocoumaroyl-*N*’-dihydrocaffeoyl spermidine, as it exhibited a [M + H]^+^ ion at *m*/*z* 458.26, and fragment ions at *m*/*z* 441.24 [M + H-NH_3_]^+^, 310.21 [M + H-dihydrocoumaroyl]^+^, 293.19 [M + H-NH_3_-dihydrocoumaroyl]^+^, 222.11, 206.12, 165.05 [dihydrocaffeoyl]^+^, 123.04, and 72.08; and a [M-H]^−^ ion at *m*/*z* 456.25, with fragment ions at *m*/*z* 334.41 [M-H-122]^−^, 308.20 [M-H-dihydrocoumaroyl]^−^, 186.16 [M-H-122-dihydrocoumaroyl]^−^, and 121.03. Based on the same premises as compound **44**, a non-linear (N^1^–N^5^– or N^5^–N^10^–) configuration of this compound is proposed. Moreover, compound **66** ([M + H]^+^ ion at *m*/*z* 536.28) was tentatively identified as *N*-dihydrocoumaroyl-*N*’-dihydrocaffeoyl spermidine conjugate (+C_5_H_2_O). It presents a characteristic ion of *N*-dihydrocoumaroyl-*N*’-dihydrocaffeoyl spermidine (at *m*/*z* 441.24 [M + H-C_5_H_5_NO]^+^), and other related fragment ions at *m*/*z* 293.18 [M + H-C_5_H_5_NO-dihydrocoumaroyl]^+^, 277.19 [M + H-C_5_H_5_NO-dihydrocaffeoyl]^+^, 222.11, 206.12, and 165.05 [dihydrocaffeoyl]^+^ in ESI (+). It also presents fragment ions at *m*/*z* 439.22 [M-H-C_5_H_5_NO]^−^, 386.20 [M-H-dihydrocoumaroyl]^−^, 291.17 [M-H-C_5_H_5_NO-dihydrocoumaroyl]^−^, and 94.03 in ESI (-). Additionally, compound **79** ([M + H]^+^ ion at *m*/*z* 500.27) and compound **91** ([M + H]^+^ ion at *m*/*z* 536.28) were tentatively identified as *N*-dihydrocoumaroyl-*N*’-dihydrocaffeoyl spermidine conjugates (+C_2_H_2_O for compound **79** and +C_5_H_2_O for compound **90**). These inferences were based on the presence of fragment ions at *m*/*z* 222.11 and 165.05 and neutral losses of 148.05 (dihydrocoumaroyl, –C_9_H_8_O_2_) in ESI (+); and fragment ions at *m*/*z* 121.03 and neutral losses of 148.05 (dihydrocoumaroyl, –C_9_H_8_O_2_) and 122.04 (–C_7_H_6_O_2_) in ESI (−) (see Table S9, Supplementary Materials for more details). However, as none of these compounds present the characteristic ion of the non-linear (N^1^–N^5^– or N^5^–N^10^–) configuration of *N*-dihydrocoumaroyl-*N*’-dihydrocaffeoyl spermidine (*m*/*z* at 441.24), a linear (N^1^–N^10^–) configuration for these compounds is proposed.

Lastly, compound **58** (Figure 4d) was tentatively identified as *N*-dihydroferuloyl-*N*’-dihydrocaffeoyl spermidine, as it exhibited a [M + H]^+^ ion at *m*/*z* 488.28, and has fragment ions at *m*/*z* 471.25 [M + H-NH_3_]^+^, 310.21 [M + H-dihydroferuloyl]^+^, 292.20 [M + H-dihydroferuloyl-NH_4_]^+^, 222.11, 179.07 [dihydroferuloyl]^+^, 165.05 [dihydrocaffeoyl]^+^, and 72.08; and the [M-H]^−^ ion at *m*/*z* 486.26 produced three major fragment ions at *m*/*z* 468.17 [M-H-H_2_O]^−^, 441.29, and 308.21 [M-H-dihydroferuloyl]^−^. On the other hand, compound **65** was tentatively identified as *N*-feruroyl-*N*’-dihydrocaffeoyl spermidine. It exhibited a [M + H]^+^ ion at *m*/*z* 486.26 and fragment ions at *m*/*z* 469.23 [M + H-NH_3_]^+^, 310.21 [M + H-feruloyl]^+^, 293.19 [M + H-NH_3_-feruloyl]^+^, 222.11, and 177.06 [feruloyl]^+^; and a [M-H]^−^ ion at *m*/*z* 484.24, producing two major fragment ions at *m*/*z* 308.20 [M-H-feruloyl]^−^ and 175.04 [feruloyl]^−^.

Pentacyclic triterpenoid saponins, such as hederagenin, oleanolic acid, and their respective glucosides, were the second main class of compounds tentatively identified in pracaxi cake. Hederagenin is an olean-12-en-28-oic acid substituted by a beta-hydroxy group at positions 3 and 23 while oleanolic acid is only substituted by a beta-hydroxy group at position 3, and both compounds were previously reported in pracaxi nuts [42]. Compound **103**, which presented ions at *m*/*z* 495.35 [M + Na]^+^, 455.35 [M + H-H_2_O]^+^, 437.34 [M + H-H_2_O-H_2_O]^+^, 945.72 [2M + H]^+^, and 967.70 [2M + Na]^+^, was identified as hederagenin. Based on the same *in-source* fragmentation pattern, compounds **92**, **93**, **94**, **96**, and **98**, were tentatively identified as hederagenin glucosides, with different sugar moieties: C_6_H_10_O_5_ (+162.05), C_5_H_8_O_4_ (+132.04), and/or C_6_H_10_O_4_ (+146.06). Compound **92** was tentatively identified as hederagenin tetraglucoside. In ESI (−), this compound exhibits a [M-H]^−^ ion at *m*/*z* 1073.55 and several adduct ions at *m*/*z* 1109.53 [M + Cl]^−^, 1136.55 [M-H + HNO_3_]^−^, and 1191.49 [M-H + 118]^−^. Among them, the ion at *m*/*z* 1073.55 [M-H]^−^ produced major fragment ions at *m*/*z* 911.50 [M-H-162.05]^−^, 765.44 [M-H-162.05-146.06]^−^, 749.45 [M-H-162.05-162.05]^−^, 603.39 [M-H-162.05-162.05-146.06]^−^, 585.37 [M-H-162.05-162.05-146.06-H_2_O]^−^, and 471.35 [M-H-162.05-162.05-146.06-132.04]^−^; and the ion at *m*/*z* 1136.55 [M-H + HNO_3_]^−^ produced two major fragments at *m*/*z* 1073.55 [M-H]^−^ and 911.50 [M-H-162.05]^−^. Complementary, the ESI (+) analysis shows a protonated [M + H]^+^ ion at *m*/*z* 1075.57, and *in-source* fragment ions at *m*/*z* 913.52 [M + H-162.05]^+^, 751.46 [M + H-162.05-162.05]^+^, 455.3532 [M + H-162.05-162.05-146.06-132.04-H_2_O]^+^, and 437.34 [M + H-162.05-162.05-146.06-132.04-H_2_O-H_2_O]^+^. Moreover, the ion at *m*/*z* 913.52 [M + H-162.05]^+^ produced three fragment ions at *m*/*z* 751.47 [M + H-162.05-162.05]^+^, 455.35 [M + H-162.05-162.05-146.06-132.04-H_2_O]^+^, and 437.34 [M + H-162.05-162.05-146.06-132.04-H_2_O-H_2_O]^+^; and the ion at *m*/*z* 751.46 [M + H-162.05-162.05]^+^ produced three fragment ions at *m*/*z* 455.35 [M + H-162.05-162.05-146.06-132.04-H_2_O]^+^, 437.34 [M + H-162.05-162.05-146.06-132.04-H_2_O-H_2_O]^+^ and 279.11. This MS/MS fragmentation pattern agrees with previous studies reported by Viana et al. (2004) [42]. The authors showed different triterpenoid saponins in the stem bark of the pracaxi tree. Glucose (C_6_H_10_O_5_), rhamnose (C_6_H_10_O_4_), and arabinose (C_5_H_8_O_4_) were the main sugars determined in these complex compounds. Based on this information, a hederagenin tetraglucoside with a branched sugar chain involving a two-terminal glucopyranosyl (Glu), one rhamnopyranosyl (Rha), and one arabinopyranosyl (Ara) moieties was proposed for compound **92**. Additionally, compound **93** was tentatively identified as hederagenin triglucoside (Ara-Rha-Glu), compound **94** as hederagenin diglucoside (Ara-Glu), compound **96** as alpha-hederin (hederagenin diglucoside, Ara-Rha), and compound **98** as hederagenin monoglucoside (Glu). In addition, the elution time of these compounds (**92** < **93** < **94** < **96** < **98** < **103**) agrees with their glycosylated degree. The most glycosylated form (compound **92**) was the first one to elute, and the aglycon form (compound **103**, hederagenin) was the last one.

Similarly to hederagenin, compound **110** was identified as oleanolic acid by MS-DIAL software, with several adduct and *in-source* fragment ions matching to this compound, such as *m*/*z* at 479.35 [M + Na]^+^, 439.36 [M + H-H_2_O]^+^, and 935.71 [2M + Na]^+^. Based on the same *in-source* fragmentation pattern, and after the manual interpretation of the acquired MS/MS spectra, compounds **95**, **97**, **99**, **101**, and **105**, were tentatively identified as oleanolic acid glucosides, with different sugar residues. Compound **95**, for instance, was tentatively identified as oleanolic acid tetraglucoside (Ara-Rha-Glu-Glu). In ESI (−), this compound exhibited a [M-H]^−^ ion at *m*/*z* 1057.56, and several adducts ions at *m*/*z* 1093.53 [M + Cl]^−^, 1120.55 [M-H + HNO_3_]^−^, and 1175.50 [M-H + 118]^−^. The ion at *m*/*z* 1057.56 [M-H]^−^ produced four major fragment ions at *m*/*z* 895.51 [M-H-162.05]^−^, 733.45 [M-H-162.05-162.05]^−^, 587.40 [M-H-162.05-162.05-146.06]^−^, and 455.35 [M-H-162.05-162.05-146.06-132.04-]. The ion at *m*/*z* 1093.53 [M + Cl]^−^, produced three major fragment ions at *m*/*z* 1057.56 [M-H]^−^, 895.51 [M-H-162.05]^−^, and 733.45 [M-H-162.05-162.05]^−^. Complementarily, the ESI (+) analysis shows a [M + Na]^+^ ion at *m*/*z* 1081.56 and a [M + NH_4_]^+^ ion at *m*/*z* 1076.60. Compound **97** was tentatively identified as oleanolic acid triglucoside (Ara-Rha-Glu), compound **99** as oleanolic acid diglucoside (Ara-Glu), compound **101** as beta-hederin (oleanolic acid diglucoside, Ara-Rha), and compound **105** as oleanolic acid monoglucoside (Ara). In addition, the elution time of these compounds (**95** < **97** < **99** < **101** < **105** < **110**) is related with their degree of glycosation, being the most glycosylated form (oleanolic acid tetraglucoside, compound **95**) the first to elute, and the aglycon form (oleanolic acid, compound **110**) the last. In addition to the similarity between hederagenin and oleanolic acid, both compounds have the same combination of sugar residues (Glu, Rha, and Ara). It is expected that these sugar moieties are the same as those present in spermidine phenolamides glucoside derivatives (compounds **43**, **45**, and **51**).

Finally, the metabolic composition of the PLE12.5 and PLE80 extracts was further analyzed by PCA, PLS-DA, and *t*-test to identify differences between the two optimum extraction conditions. The PCA established two principal components (PC1/PC2) from the metabolites in the different PLE extracts, explaining 47.0% (PC1) and 29.4% (PC2) of the variance for ESI (+), and 49.8% (PC1) and 33.2% (PC2) of the variance for ESI (−) analyses (Figure S3, Supplementary Materials). Given the good separation using the PCA, the supervised PLS-DA analysis was further applied to classify the samples, to estimate the importance of each metabolite in the separation of the two groups (based on the VIP values), and to identify those metabolites mostly affected by the different EtOH percentages used during the extraction (Figure S4, Supplementary Materials). Based on the “Leave-one-out” cross-validation method, one component was selected for ESI (+) (R^2^ = 0.971, Q^2^ = 0.725) and ESI (−) (R^2^ = 0.967, Q^2^ = 0.754) analyses. These values indicate that the variability is well explained in both models, and the predictive ability of these models is good. According to these models, 32 and 21 metabolites had VIP values > 1.2 in ESI (+) and ESI (−) analyses, respectively (Table 3). Moreover, the two-sample *t*-test univariate analysis shows 32 and 21 metabolites with *p* < 0.05 in ESI (+) and ESI (−), respectively. A total of 40 differentially accumulated compounds was determined in PLE12.5 and PLE80 extracts. There was a significant enrichment of sugars (D-lyxose) and glycosylated forms, such as compounds **45**, **94**, **95**, **98**, **99**, **105**, and **114**, and their aglycon forms (hederagenin and oleanolic acid) in PLE12.5 extract. The higher water content (87.5%) used during PLE12.5 extraction probably facilitated the extraction of compounds with higher polarity, such as sugars and glycosylated compounds. Two *N*-*N***′**-bis-(dihydrocaffeoyl) spermidine conjugates (compounds **68** and **75**), two ethanolamides (compounds **106** and **108**), glycerophosphocholine, and compounds **49** and **79** were also determined in high concentrations in PLE12.5. Previous studies demonstrated that oleanolic acid, hederagenin, and their saponin derivatives have anti-inflammatory properties. It may explain the slightly higher LOX inhibitory activity observed for PLE12.5 extract [43,44]. These results also suggest that PLE 12.5 extract can be evaluated for its *in vitro* anti-inflammatory capacity in future studies. On the other hand, the PLE80 extract was significantly enriched in several spermidine phenolamides, such as *N*-*N***′**-bis-(dihydrocaffeoyl) spermidine derivatives (compounds **43**, **46**, **54**, **59**, **61**, **67**, **69**, **71**, **72**, **74**, **78**, **83**, **84**, and **86**) and *N*-coumaroyl-*N***′**-dihydrocaffeoyl spermidine. The higher abundance of these spermidine phenolamides derivatives in the PLE80 extracts could explain the lower IC_50_ values obtained for BChE. The interaction or inhibition of ChE enzymes by spermidine phenolamides was not reported yet and it can also be explored in future studies. Moreover, previous studies demonstrated that phenolamides have ROS-scavenging capacity, and this activity is dependent on the type and position of the (dihydro) hydroxycinnamic acid moieties. It could explain the extraordinary antioxidant capacity exerted by both PLE extracts.

## 4. Conclusions

In conclusion, a biorefinery process including two-sequential green extraction techniques (SFE and PLE), different advanced analytical techniques (HPLC-Q-TOF MS/MS and GC-Q-TOF MS), and diverse *in vitro* assays were successfully applied in the present study. This strategy was used to extract, characterize, and evaluate the potential properties of bioactive compounds present in pracaxi oil and cake. After the SFE-CO_2_ step, the chemical characterization of the resulting oil allowed the annotation of more than 220 compounds. Therefore, this work represents the most comprehensive study ever performed on this matrix. Thereafter, a PLE method was optimized to re-extract and revalorize the cake obtained after the extraction with SFE-CO_2_. Temperature was the main parameter affecting the extraction yield, total phenolic content, and anti-cholinesterase and antioxidant capacities. On the other hand, results show that the extraction yield is not necessarily associated with the recovery of compounds with higher bioactivity. The extracts obtained at two optimum extraction conditions (80% and 12.5% of EtOH at 180 °C) showed interesting *in vitro* antioxidant and anti-inflammatory capacities. Moreover, it is the first time that a moderate anti-cholinesterase activity is reported for pracaxi, showing promising results. Finally, different triterpenoid saponins, and for the first time, several spermidine phenolamides, were tentatively identified in the extracts obtained from pracaxi nuts. All these compounds might be responsible for the *in vitro* activities observed, indicating that pracaxi oil and its co-products are a valuable source of bioactive compounds with neuroprotective activity.

## Figures and Tables

**Table 1:** Response variables (extraction yield, total phenolic content, ROS scavenging capacity, and AChE inhibitory activity) used for optimization of the conditions for extraction of bioactive compounds from pracaxi cake by PLE.

Sample	Temperature (°C)	Solvent Composition	Extraction Yield (%)	TPC (mg GAE/g)	ROS (IC_50_ μg/mL)	AChE (IC_50_ μg/mL)
1	115	50% EtOH	12.2	147.5 ± 1.2	4.7 ± 0.3	334 ± 28
2	115	50% EtOH	12.0	108.1 ± 4.4	3.0 ± 0.2	315 ± 32
3	180	50% EtOH	17.4	167.4 ± 2.5	2.4 ± 0.1	247 ± 25
4	50	50% EtOH	7.1	99.7 ± 1.2	11.3 ± 1.4	301 ± 16
5	115	100% water	7.2	112.3 ± 4.4	5.5 ± 0.7	395 ± 48
6	180	100% water	14.4	176.5 ± 2.1	1.9 ± 0.3	378 ± 28
7	115	100% EtOH	17.9	116.6 ± 5.3	3.1 ± 0.2	1018 ± 126
8	115	50% EtOH	13.7	134.9 ± 1.2	3.4 ± 0.4	320 ± 5
9	180	100% EtOH	22.8	163.8 ± 9.2	2.7 ± 0.1	342 ± 25
10	115	50% EtOH	14.0	144.8 ± 0.1	3.2 ± 0.3	352 ± 9
11	50	100% water	3.5	75.0 ± 14.7	11.4 ± 1.2	629 ± 26
12	50	100% EtOH	8.2	122.2 ± 3.2	3.0 ± 0.9	1071 ± 33

TPC: Total phenolic content; ROS: reactive oxygen species; and AChE: acetylcholinesterase.

**Table 2:** Extraction yield, TPC, TFC, and neuroprotective potential evaluation of extracts obtained from pracaxi cake obtained by PLE under optimized conditions.

Sample	Extraction Yield (%)	TPC (mg GAE/g)	TFC (mg QE/g)	ROS (IC_50_ μg/mL)	RNS (IC_50_ μg/mL)	AChE (IC_50_ μg/mL)	BChE (IC_50_ μg/mL)	LOX (IC_50_ μg/mL)
PLE80	24.0 ± 3.0 *	91.9 ± 0.9	6.6 ± 0.2	1.5 ± 1.0	2092 ± 217	276 ± 17	348 ± 25 *	18.6 ± 1.0
PLE12.5	15.9 ± 1.8	103.9 ± 0.5	7.0 ± 0.1 *	1.6 ± 1.0	2559 ± 331	315 ± 29	457 ± 38	14.9 ± 1.2 *
Galantamine						0.8 ± 0.1	3.1 ± 0.3	
Quercetin								12.2 ± 0.7
Ascorbic acid				3.2 ± 0.2	1120 ± 16			

PLE 80: extract obtained at 80% EtOH and 180 °C; PLE12.5: extract obtained at 12.5% EtOH and 180 °C; TPC: total phenolic content; TFC: total flavonoid content; ROS: reactive oxygen species; RNS; reactive nitrogen species; AChE: acetylcholinesterase; BChE: butyrylcholinesterase; LOX: lipoxygenase; GAE: gallic acid equivalents; and QE: quercetin equivalents. Asterisks indicate significant differences between PLE80 vs. PLE12.5 extracts (for each assay) after a two-sample *t*-test, *p*-value < 0.05.

**Table 3:** Chemical composition of pracaxi cake PLE extracts obtained after HPLC-C18-Q-TOF MS/MS ESI (+/−) analyses.

No	Tentative Compound Name	Molecular Formula	HPLC-C18-Q-TOF MS ESI (+)				HPLC-C18-Q-TOF MS ESI (−)			
			RT	PLE80/PLE12.5			RT	PLE80/PLE12.5		
				VIP	FC	*p*-Val		VIP	FC	*p*-Val
**1**	Agmatine	C_5_H_14_N_4_	0.497	0.16	1.05	0.840				
**2**	L-Arginine	C_6_H_14_N_4_O_2_	0.537	0.40	0.87	0.612				
**3**	Serine	C_3_H_7_NO_3_					0.545	0.90	0.73	0.189
**4**	D-Asparagine	C_4_H_8_N_2_O_3_					0.552	0.52	0.61	0.482
**5**	Threonine	C_4_H_9_NO_3_	0.554	0.07	1.02	0.929				
**6**	D-Arabinonic acid	C_5_H_10_O_6_					0.571	0.32	0.84	0.675
**7**	Galactonic acid	C_6_H_12_O_7_					0.572	0.18	0.90	0.811
**8**	Choline cation	C_5_H_14_NO	0.585	0.01	1.00	0.997				
**9**	**D-Lyxose**	C_5_H_10_O_5_					0.611	**1.33**	**0.16**	**0.009**
**10**	**Glycerophosphocholine**	C_8_H_20_NO_6_P	0.616	**1.39**	**0.46**	**0.007**				
**11**	L-Monomethylarginine	C_7_H_16_N_4_O_2_	0.618	0.78	0.82	0.284				
**12**	4-O-.beta.-Galactopyranosyl-D-mannopyranose	C_12_H_22_O_11_	0.634	0.66	0.91	0.379	0.639	0.58	0.94	0.428
**13**	Trigonelline	C_7_H_7_NO_2_	0.650	1.20	0.82	0.054				
**14**	3-Hydroxypyridine	C_5_H_5_NO	0.656	0.76	1.14	0.301				
**15**	5-Hydroxy-2-methylpyridine	C_6_H_7_NO	0.673	0.85	0.84	0.236				
**16**	Betaine	C_5_H_11_NO_2_	0.674	0.46	1.21	0.551				
**17**	Malic acid	C_4_H_6_O_5_					0.677	0.82	1.18	0.242
**18**	**Cadaverine**	C_5_H_14_N_2_	0.678	**1.44**	**2.69**	**0.002**				
**19**	His-Pro	C_11_H_16_N_4_O_3_	0.680	0.01	1.00	0.999				
**20**	***N*** **-Methyl-L-leucine**	C_7_H_15_NO_2_	0.681	**1.31**	**32.87**	**0.021**				
**21**	D-Pyroglutamic acid	C_5_H_7_NO_3_					0.690	0.13	1.03	0.867
**22**	alpha-Cyclopropyl-3-pyridinemethanol	C_9_H_11_NO	0.692	0.34	1.33	0.664				
**23**	3-Hydroxypicolinic acid	C_6_H_5_NO_3_					0.725	0.43	1.39	0.569
**24**	Succinic acid	C_4_H_6_O_4_					1.083	0.39	0.71	0.601
**25**	2-Amino-2-methylpentanoic acid	C_6_H_13_NO_2_	1.135	0.12	1.07	0.883				
**26**	4-aminobutyrate	C_4_H_9_NO_2_	1.379	0.88	2.56	0.218				
**27**	Itaconic acid	C_5_H_6_O_4_					1.788	0.12	0.95	0.876
**28**	**Adenosine**	C_10_H_13_N_5_O_4_	1.886	**1.29**	**0.76**	**0.027**				
**29**	Guanosine	C_10_H_13_N_5_O_5_	1.969	0.23	0.88	0.766				
**30**	*N*-(2,4-Dimethylphenyl)formamide	C_9_H_11_NO	2.003	0.38	1.31	0.621				
**31**	**4-Methyl-1H-benzimidazole**	C_8_H_8_N_2_	2.055	**1.22**	**2.42**	**0.048**				
**32**	L-Phenylalanine	C_9_H_11_NO_2_	2.079	0.97	0.56	0.164				
**33**	1,3-Dimethyl-1H-pyrazole-4-carbaldehyde	C_6_H_8_N_2_O	2.361	0.22	1.11	0.778				
**34**	5-Hydroxymethylfurfural	C_6_H_6_O_3_	2.650	1.00	1.73	0.146				
**35**	Catechol	C_6_H_6_O_2_					2.661	0.40	0.77	0.597
**36**	5,6-Dimethylbenzimidazole	C_9_H_10_N_2_	2.712	0.55	1.54	0.475				
**37**	L-Tryptophan	C_11_H_12_N_2_O_2_	2.846	0.03	1.03	0.974				
**38**	*N*-Acetyl-DL-valine	C_7_H_13_NO_3_	3.014	0.14	1.09	0.860				
**39**	*N*-L-amma-Glutamyl-L-leucine	C_11_H_20_N_2_O_5_	3.061	0.12	1.08	0.878	2.903	0.50	0.86	0.507
**40**	**3,4-Dihydroxybenzaldehyde**	C_7_H_6_O_3_					3.033	**1.30**	**0.40**	**0.014**
**41**	*N*-(3-(Aminomethyl)benzyl)acetamidine	C_10_H_15_N_3_	3.125	1.10	1.73	0.095				
**42**	Diethyl L-glutamate	C_9_H_17_NO_4_	3.164	1.09	8.28	0.097				
**43**	***N*** **-*N*′-bis-(dihydrocaffeoyl) spermidine-monoglucoside (+C_6_H_10_O_5_)**	C_31_H_45_N_3_O_11_	3.766	0.73	1.76	0.320	3.604	**1.29**	**3.37**	**0.016**
**44**	*N*-*N*′-bis-(dihydrocaffeoyl) spermidine isomer 1	C_25_H_35_N_3_O_6_	4.013	0.70	0.85	0.350	3.657	0.96	1.69	0.152
**45**	***N*** **-*N*′-bis-(dihydrocaffeoyl) spermidine-monoglucoside (+C_5_H_8_O_4_)**	C_30_H_43_N_3_O_10_	4.096	**1.45**	**0.34**	**0.001**	3.726	**1.31**	**0.41**	**0.013**
**46**	***N*** **-*N*′-bis-(dihydrocaffeoyl) spermidine-conjugate (+CH_2_)**	C_26_H_37_N_3_O_6_	4.148	**1.30**	**3.37**	**0.024**	3.763	**1.40**	**8.70**	**0.001**
**47**	*N*-*N*′-bis-(dihydrocaffeoyl) spermidine isomer 2	C_25_H_35_N_3_O_6_	4.230	0.88	3.40	0.214	3.891	0.56	1.72	0.452
**48**	***N*** **-coumaroyl-*N*′-dihydrocaffeoyl spermidine**	C_25_H_33_N_3_O_5_	4.231	**1.24**	**3.46**	**0.040**	3.840	1.06	2.42	0.099
**49**	***N*** **-caffeoyl-*N*′-dihydrocaffeoyl spermidine-conjugate (+C_5_H_2_O)**	C_30_H_35_N_3_O_7_	4.242	**1.33**	**0.01**	**0.017**	3.886	**1.43**	**0.06**	**0.001**
**50**	*N*-caffeoyl-*N*′-dihydrocaffeoyl spermidine isomer 1	C_25_H_33_N_3_O_6_	4.303	0.66	1.51	0.376	3.945	1.00	1.88	0.128
**51**	*N*-*N*′-bis-(dihydrocaffeoyl) spermidine-monoglucoside (+C_6_H_10_O_4_)	C_31_H_45_N_3_O_10_	4.327	1.11	0.41	0.087				
**52**	*N*-*N*′-bis-(dihydrocaffeoyl) spermidine-conjugate (+C_2_H_4_)	C_27_H_39_N_3_O_6_	4.334	1.10	5.49	0.093	3.964	1.01	8.35	0.123
**53**	*N*-dihydrocoumaroyl-*N*′-dihydrocaffeoyl spermidine	C_25_H_35_N_3_O_5_	4.440	0.03	0.99	0.973	4.061	0.70	1.82	0.332
**54**	***N*** **-*N*′-bis-(dihydrocaffeoyl) spermidine-conjugate (+C_5_H_2_)**	C_30_H_37_N_3_O_6_	4.468	**1.46**	**1.94**	**0.001**				
**55**	*N*-caffeoyl-*N*′-dihydrocaffeoyl spermidine isomer 2	C_25_H_33_N_3_O_6_	4.519	0.74	1.68	0.315	4.167	0.89	4.82	0.193
**56**	*N*-*N*′-bis-(dihydrocaffeoyl) spermidine-conjugate (+C_4_H_6_)	C_29_H_41_N_3_O_6_	4.538	1.05	37.36	0.117				
**57**	*N*-*N*′-bis-(dihydrocaffeoyl) spermidine-conjugate (+C_5_H_2_O) isomer 1	C_30_H_37_N_3_O_7_	4.634	0.85	0.69	0.236	4.313	0.21	1.16	0.785
**58**	*N*-dihydroferuloyl-*N*′-dihydrocaffeoyl spermidine	C_26_H_37_N_3_O_6_	4.637	0.38	1.47	0.627	4.253	0.68	1.57	0.349
**59**	***N*** **-*N*′-bis-(dihydrocaffeoyl) spermidine-conjugate (+C_4_H_5_N)**	C_29_H_40_N_4_O_6_	4.679	**1.39**	**1.73**	**0.006**				
**60**	*N*-*N*′-bis-(dihydrocaffeoyl) spermidine-conjugate (+C_4_H_3_N)	C_29_H_38_N_4_O_6_	4.763	0.83	0.76	0.253	4.374	0.81	0.66	0.248
**61**	***N*** **-*N*′-bis-(dihydrocaffeoyl) spermidine-conjugate (+C_4_H_6_O_2_)**	C_29_H_41_N_3_O_8_	4.834	**1.24**	**2.50**	**0.038**	4.425	**1.41**	**12.75**	**0.001**
**62**	*N*-*N*′-bis-(dihydrocaffeoyl) spermidine-conjugate (+C_6_H_5_N) isomer 1	C_31_H_40_N_4_O_6_	4.831	0.99	1.79	0.153				
**63**	*N*-*N*′-bis-(dihydrocaffeoyl) spermidine-conjugate (+C_6_H_9_NO)	C_31_H_44_N_4_O_7_	4.888	1.11	1.27	0.090				
**64**	*N*-*N*′-bis-(dihydrocaffeoyl) spermidine-conjugate (+C_5_H_5_N)	C_30_H_40_N_4_O_6_	4.941	0.40	1.08	0.611	4.560	0.60	1.46	0.415
**65**	*N*-feruroyl-*N*′-dihydrocaffeoyl spermidine	C_26_H_35_N_3_O_6_	4.954	0.28	1.15	0.721	4.563	0.65	1.57	0.371
**66**	*N*-dihydrocoumaroyl-*N*′-dihydrocaffeoyl spermidine conjugate (+C_5_H_2_O)	C_30_H_37_N_3_O_6_	5.065	1.19	0.42	0.056	4.719	0.99	0.49	0.136
**67**	***N*** **-*N*′-bis-(dihydrocaffeoyl) spermidine-conjugate (+C_5_H_3_N)**	C_30_H_38_N_4_O_6_	5.078	**1.46**	**56.70**	**0.001**	4.749	**1.35**	**23.37**	**0.006**
**68**	***N*** **-*N*′-bis-(dihydrocaffeoyl) spermidine-conjugate (+C_7_H_7_N)**	C_32_H_42_N_4_O_6_	5.136	**1.30**	**0.64**	**0.022**	4.738	1.00	0.47	0.126
**69**	***N*** **-*N*′-bis-(dihydrocaffeoyl) spermidine-conjugate (+C_5_H_8_O_2_)**	C_30_H_43_N_3_O_8_	5.190	**1.40**	**1.83**	**0.005**	4.796	**1.24**	**2.45**	**0.030**
**70**	*N*-*N*′-bis-(dihydrocaffeoyl) spermidine-conjugate (+C_6_H_7_N)	C_31_H_42_N_4_O_6_	5.344	1.11	3.29	0.089	4.951	1.09	6.91	0.081
**71**	***N*** **-*N*′-bis-(dihydrocaffeoyl) spermidine-conjugate (+C_6_H_5_N) isomer 2**	C_31_H_40_N_4_O_6_	5.351	**1.41**	**2.27**	**0.004**	4.984	0.91	4.54	0.181
**72**	***N*** **-*N*′-bis-(dihydrocaffeoyl) spermidine-conjugate (+CO)**	C_26_H_35_N_3_O_7_	5.388	0.79	1.29	0.280	5.339	**1.27**	**2.63**	**0.022**
**73**	*N*-*N*′-bis-(dihydrocaffeoyl) spermidine-conjugate (+C_8_H_7_N)	C_33_H_42_N_4_O_6_	5.395	1.14	0.59	0.076				
**74**	***N*** **-*N*′-bis-(dihydrocaffeoyl) spermidine-conjugate (+C_8_H_9_N) isomer 1**	C_33_H_44_N_4_O_6_	5.543	**1.35**	**2.39**	**0.013**				
**75**	***N*** **-*N*′-bis-(dihydrocaffeoyl) spermidine-conjugate (+C_2_H_2_O)**	C_27_H_37_N_3_O_7_	5.563	**1.46**	**0.61**	**0.001**	5.511	0.97	0.80	0.147
**76**	*N*-*N*′-bis-(dihydrocaffeoyl) spermidine-conjugate (+C_4_H_2_O_3_)	C_29_H_37_N_3_O_9_	5.747	1.06	3.21	0.113	5.697	1.00	4.37	0.127
**77**	*N*-*N*′-bis-(dihydrocaffeoyl) spermidine-conjugate (+C_8_H_9_N) isomer 2	C_33_H_44_N_4_O_6_	5.756	0.88	2.36	0.219				
**78**	***N*** **-*N*′-bis-(dihydrocaffeoyl) spermidine-conjugate (+C_6_H_4_O_5_)**	C_31_H_39_N_3_O_11_	5.824	**1.34**	**4.68**	**0.016**	5.783	**1.24**	**5.50**	**0.029**
**79**	***N*** **-dihydrocoumaroyl-*N*′-dihydrocaffeoyl spermidine-conjugate (+C_2_H_2_O)**	C_27_H_37_N_3_O_6_	6.058	**1.48**	**0.33**	**0.001**	6.010	**1.35**	**0.50**	**0.006**
**80**	*N*-*N*′-bis-(dihydrocaffeoyl) spermidine-conjugate (+C_8_H_6_O_3_)	C_33_H_41_N_3_O_9_	6.160	1.18	7.49	0.061				
**81**	*N*-*N*′-bis-(dihydrocaffeoyl) spermidine-conjugate (+C_4_H_2_O_2_)	C_29_H_37_N_3_O_8_	6.230	0.78	1.75	0.286	6.179	0.85	2.11	0.222
**82**	*N*-*N*′-bis-(dihydrocaffeoyl) spermidine-conjugate (+C_3_H_2_O_2_)	C_28_H_37_N_3_O_8_	6.233	0.09	1.05	0.908	6.188	0.49	1.29	0.515
**83**	***N*** **-*N*′-bis-(dihydrocaffeoyl) spermidine-conjugate (+C_6_H_4_O_2_)**	C_31_H_39_N_3_O_8_	6.944	1.19	1.28	0.056	6.897	**1.21**	**1.98**	**0.039**
**84**	***N*** **-*N*′-bis-(dihydrocaffeoyl) spermidine-conjugate (+C_8_H_8_O_5_)**	C_33_H_43_N_3_O_11_	7.216	**1.24**	**35.78**	**0.041**	7.169	1.09	62.32	0.085
**85**	*N*-*N*′-bis-(dihydrocaffeoyl) spermidine-conjugate (+C_6_H_5_N) isomer 3	C_31_H_40_N_4_O_6_	7.359	1.14	3.39	0.077	7.423	1.11	4.03	0.073
**86**	***N*** **-*N*′-bis-(dihydrocaffeoyl) spermidine-conjugate (+C_5_H_2_O_2_)**	C_30_H_37_N_3_O_8_	7.610	**1.27**	**5.69**	**0.031**	7.562	1.14	7.82	0.063
**87**	*N*-*N*′-bis-(dihydrocaffeoyl) spermidine-conjugate (+C_5_H_2_O) isomer 2	C_30_H_37_N_3_O_7_	7.930	0.05	0.98	0.950	7.886	0.58	1.47	0.431
**88**	*N*-*N*′-bis-(dihydrocaffeoyl) spermidine-conjugate (+C_4_H_2_)	C_29_H_37_N_3_O_6_	8.246	1.02	1.71	0.136	8.224	1.10	2.89	0.079
**89**	*N*-*N*′-bis-(dihydrocaffeoyl) spermidine-conjugate (+C_6_H_4_O)	C_31_H_39_N_3_O_7_	8.257	0.71	0.78	0.337	8.234	0.17	1.06	0.822
**90**	*N*-dihydrocoumaroyl-*N*′-dihydrocaffeoyl spermidine-conjugate (+C_5_H_2_O)	C_30_H_37_N_3_O_6_	8.278	1.07	0.56	0.109	8.254	0.53	0.79	0.472
**91**	*N*-*N*′-bis-(dihydrocaffeoyl) spermidine-conjugate (+C_8_H_8_O_2_)	C_33_H_43_N_3_O_8_	8.452	0.69	1.78	0.359	8.437	0.73	2.00	0.309
**92**	Hederagenin-tetraglucoside	C_53_H_86_O_22_	8.851	1.14	0.80	0.076	8.835	1.12	0.83	0.073
**93**	Hederagenin-triglucoside	C_47_H_76_O_17_	8.952	1.10	0.80	0.092	8.936	0.74	0.91	0.300
**94**	**Hederagenin-diglucoside**	C_41_H_66_O_13_	9.078	**1.39**	**0.29**	**0.006**	9.061	**1.29**	**0.33**	**0.016**
**95**	**Oleanolic acid-tetraglucoside**	C_53_H_86_O_21_	9.199	**1.28**	**0.78**	**0.028**	9.180	**1.33**	**0.90**	**0.009**
**96**	alpha-hederin	C_41_H_66_O_12_	9.245	0.90	0.86	0.207	9.247	1.04	0.95	0.106
**97**	Oleanolic acid-triglucoside	C_47_H_76_O_16_	9.353	1.11	1.28	0.090	9.369	1.12	1.33	0.071
**98**	**Hederagenin-monoglucoside**	C_35_H_56_O_8_	9.395	**1.36**	**0.30**	**0.011**	9.393	**1.34**	**0.29**	**0.008**
**99**	**Oleanolic acid-diglucoside**	C_41_H_66_O_12_	9.566	**1.31**	**0.37**	**0.021**	9.543	1.17	0.49	0.051
**100**	1,2-dioleoyl-sn-glycero-3-phosphatidylcholine	C_44_H_84_NO_8_P	9.665	0.26	1.07	0.741				
**101**	Beta-hederin	C_41_H_66_O_11_	9.732	0.09	0.97	0.906	9.767	0.75	1.12	0.292
**102**	1-Oleoyl-2-palmitoyl-sn-glycero-3-phosphocholine	C_42_H_82_NO_8_P	9.816	0.48	1.26	0.532				
**103**	**Hederagenin**	C_30_H_48_O_4_	9.835	**1.48**	**0.11**	**0.001**	9.823	**1.41**	**0.16**	**0.001**
**104**	1-Palmitoyl-sn-glycero-3-phosphocholine	C_24_H_50_NO_7_P	9.852	0.40	0.57	0.611				
**105**	**Oleanolic acid-monoglucoside**	C_35_H_56_O_7_	10.003	0.55	0.66	0.472	9.983	**1.32**	**0.30**	**0.011**
**106**	**Palmitoyl ethanolamide**	C_18_H_37_NO_2_	10.581	**1.30**	**0.40**	**0.023**				
**107**	1-Oleoyl-sn-glycero-3-phosphocholine	C_26_H_52_NO_7_P	10.689	0.19	1.41	0.808				
**108**	***N*** **-Oleoylethanolamine**	C_20_H_39_NO_2_	10.700	**1.22**	**0.43**	**0.046**				
**109**	**16-Hydroxypalmitic acid**	C_16_H_32_O_3_					10.566	**1.31**	**0.38**	**0.013**
**110**	**Oleanolic acid**	C_30_H_48_O_3_	10.701	**1.47**	**0.09**	**0.000**	10.686	**1.39**	**0.21**	**0.002**
**111**	**Triethylene glycol bis(2-ethylhexanoate)**	C_22_H_42_O_6_	10.834	**1.30**	**1.37**	**0.022**				
**112**	**Diisodecyl phthalate**	C_28_H_46_O_4_	12.202	**1.48**	**7.96**	**0.000**				
**113**	Oleic acid	C_18_H_34_O_2_					11.195	1.07	0.85	0.092
**114**	**Brassicasterol 3-monoglucoside**	C_34_H_56_O_6_					11.708	**1.40**	**0.09**	**0.002**

RT, retention time; VIP, variable importance in the projection; and FC, fold change. Compounds in bold indicate significant differences (*p*-value < 0.05 and VIP > 1.2) between PLE80 and PLE12.5 extracts.

## Data Availability

All data generated or analyzed during this study are included in this published article and its Supplementary Material files.

## Supplementary Materials

The following supplementary material can be downloaded at: https://www.mdpi.com/article/10.3390/foods12203879/s1; Table S1: Analysis of Variance of extraction yield variable for response surface modeling showing linear, quadratic and interaction relations, and coefficient for model prediction; Table S2: Analysis of Variance of TPC variable for response surface modeling showing linear, quadratic and interaction relations, and coefficient for model prediction; Table S3: Analysis of Variance of ROS variable for response surface modeling showing linear, quadratic and interaction relations, and coefficient for model prediction; Table S4: Analysis of Variance of AChE variable for response surface modeling showing linear, quadratic and interaction relations, and coefficient for model prediction; Table S5: Annotated compounds and their total compound contribution (%) in pracaxi SFE extract after HPLC-CSH-Q-TOF MS/MS ESI (−) analysis; Table S6: Annotated compounds and their total compound contribution (%) in pracaxi SFE extract after HPLC-CSH-Q-TOF MS/MS ESI (+) analysis; Table S7: Annotated compounds and their total compound contribution (%) in pracaxi SFE extract after GC-Q-TOF MS analysis; Table S8: PLE conditions, desirability and predicted response values at the optimum conditions predicted by the model, and experimental response values for the selected optimum conditions; Table S9: Tentative identified compounds in pracaxi nuts PLE extracts after HPLC-C18-Q-TOF MS/MS ESI (+/−) analyses; Figure S1: Estimated response surfaces for each response variable, and their corresponding Standardized Pareto charts: (**A**) Extraction yield (%); (**B**) TPC (mg GAE/mL); (**C**) IC_50_ ROS (µg/mL); (**D**) IC_50_ AChE (µg/mL); Figure S2: Desirability response surface to optimize response variables: (**A**) including extraction yield as response variable; (**B**) excluding extraction yield as response variable; Figure S3: PCA score plots of pracaxi nuts PLE extracts data obtained by: (**A**) HPLC-C18-Q-TOF MS/MS ESI (+); (**B**) HPLC-C18-Q-TOF MS/MS ESI (−); Figure S4: PLS-DA score plots of pracaxi nuts PLE extracts data obtained by: (**A**) HPLC-C18-Q-TOF MS/MS ESI (+); (**B**) HPLC-C18-Q-TOF MS/MS ESI (−).

## References

[1] Diniz M.B., Teixeira M.J., Ferreira e Silva A.L., Cardoso de Barrios M.L., Ferreira Lima E.B. (2019). Região Amazônica: Biodiversidade E Possibilidades De Transformação Industrial. Cad. CEPEC.

[2] Nobre Lamarão M.L., Ferreira L.M.M.C., Gyles Lynch D., Morais L.R.B., Silva-Júnior J.O.C., Ribeiro-Costa R.M. (2023). *Pentaclethra macroloba*: A Review of the Biological, Pharmacological, Phytochemical, Cosmetic, Nutritional and Biofuel Potential of This Amazonian Plant. Plants.

[3] Péter S., Friedel A., Roos F.F., Wyss A., Eggersdorfer M., Hoffmann K., Weber P. (2015). A Systematic Review of Global Alpha-Tocopherol Status as Assessed by Nutritional Intake Levels and Blood Serum Concentrations. Int. J. Vitam. Nutr. Res..

[4] Hampel H., Mesulam M., Cuello A.C., Farlow M.R., Giacobini E., Grossberg G.T., Khachaturian A.S., Vergallo A., Cavedo E., Snyder P. (2018). The Cholinergic System in the Pathophysiology and Treatment of Alzheimer’s Disease. Brain.

[5] Czapski G.A., Czubowicz K., Strosznajder J.B., Strosznajder R.P. (2015). The Lipoxygenases: Their Regulation and Implication in Alzheimer’s Disease. Neurochem. Res..

[6] Singh A., Kukreti R., Saso L., Kukreti S. (2019). Oxidative Stress: A Key Modulator in Neurodegenerative Diseases. Molecules.

[7] Pandey S.N., Singh G., Chander B., Gupta G., Saad K., Almalki W., Albratty M., Najmi A., Meraya A.M. (2022). Therapeutic Approaches of Nutraceuticals in the Prevention of Alzheimer’s Disease. J. Food Biochem..

[8] Grodzicki W., Dziendzikowska K. (2020). The Role of Selected Bioactive Compounds in the Prevention of Alzheimer’s Disease. Antioxidants.

[9] Roumani M., Duval E., Ropars A., Risler A., Robin C., Larbat R. (2020). Phenolamides: Plant Specialized Metabolites with a Wide Range of Promising Pharmacological and Health-Promoting Interests. Biomed. Pharmacother..

[10] Polmann G., Badia V., Danielski R., Salvador S., Mara J. (2021). Non-Conventional Nuts: An Overview of Reported Composition and Bioactivity and New Approaches for Its Consumption and Valorization of Co-Products. Futur. Foods.

[11] Teixeira G.L., Galvao L., Mazzutti S., Bernardo Gonçalves C., Salvador Ferreira S.R., Mara Block J. (2020). Composition, Thermal Behavior and Antioxidant Activity of Pracaxi (*Pentaclethra macroloba*) Seed Oil Obtained by Supercritical CO_2_. Biocatal. Agric. Biotechnol..

[12] Pereira E., Cravo Ferreira M., AraújoSampaio K., Grimaldi R., de Almeida Meirelles A.J., Maximo G.J. (2019). Physical Properties of Amazonian Fats and Oils and Their Blends. Food Chem..

[13] Cruz E., Demétrio Barros H.S. (2016). Germinação de Sementes de Espécies Amazônicas: Pracaxi [*Pentaclethra macroloba* (Willd.) Kuntze]. Embrapa Comun. Téc..

[14] Herrero M., Ibañez E. (2018). Green Extraction Processes, Biorefineries and Sustainability: Recovery of High Added-Value Products from Natural Sources. J. Supercrit. Fluids.

[15] Gallego R., Bueno M., Herrero M. (2019). Sub- and Supercritical Fluid Extraction of Bioactive Compounds from Plants, Food-by-Products, Seaweeds and Microalgae—An Update. Trends Anal. Chem..

[16] Herrero M., Sánchez-Camargo A.d.P., Cifuentes A., Ibáñez E. (2015). Plants, Seaweeds, Microalgae and Food by-Products as Natural Sources of Functional Ingredients Obtained Using Pressurized Liquid Extraction and Supercritical Fluid Extraction. Trends Anal. Chem..

[17] Catchpole O., Moreno T., Montañes F., Tallon S. (2018). Perspectives on Processing of High Value Lipids Using Supercritical Fluids. J. Supercrit. Fluids.

[18] Tsugawa H., Cajka T., Kind T., Ma Y., Higgins B., Ikeda K., Kanazawa M., Vandergheynst J., Fiehn O., Arita M. (2015). MS-DIAL: Data-Independent MS/MS Deconvolution for Comprehensive Metabolome Analysis. Nat. Methods.

[19] Gallego R., Valdés A., Sánchez-Martínez J.D., Suárez-Montenegro Z.J., Ibáñez E., Cifuentes A., Herrero M. (2022). Study of the Potential Neuroprotective Effect of *Dunaliella salina* Extract in SH-SY5Y Cell Model. Anal. Bioanal. Chem..

[20] Koşar M., Dorman H.J.D., Hiltunen R. (2005). Effect of an Acid Treatment on the Phytochemical and Antioxidant Characteristics of Extracts from Selected Lamiaceae Species. Food Chem..

[21] Woisky R.G., Salatino A. (1998). Analysis of Propolis: Some Parameters and Procedures for Chemical Quality Control. J. Apic. Res..

[22] Tripodo G., Ibáñez E., Cifuentes A., Gilbert-López B., Fanali C. (2018). Optimization of Pressurized Liquid Extraction by Response Surface Methodology of Goji Berry (*Lycium barbarum* L.) Phenolic Bioactive Compounds. Electrophoresis.

[23] Ou B., Hampsch-Woodill M., Prior R.L. (2001). Development and Validation of an Improved Oxygen Radical Absorbance Capacity Assay Using Fluorescein as the Fluorescent Probe. J. Agric. Food Chem..

[24] Sánchez-Martínez J.D., Bueno M., Alvarez-Rivera G., Tudela J., Ibanez E., Cifuentes A. (2021). In Vitro Neuroprotective Potential of Terpenes from Industrial Orange Juice By-Products. Food Funct..

[25] Ho S., Tang Y., Lin S., Liew Y. (2010). Evaluation of Peroxynitrite-Scavenging Capacities of Several Commonly Used Fresh Spices. Food Chem..

[26] Whent M.O.W., Ping T., Kenworthy W., Yu L. (2010). High-Throughput Assay for Detection of Soybean Lipoxygenase-1. J. Agric. Food Chem..

[27] Dührkop K., Fleischauer M., Ludwig M., Aksenov A.A., Melnik A.V., Meusel M., Dorrestein P.C., Rousu J., Böcker S. (2019). SIRIUS 4: A rapid tool for turning tandem mass spectra into metabolite structure information. Nat. Methods.

[28] Suffredini I.B., Frana S.A., Santos Á.M.M., Díaz I.E.C., Bernardi M.M. (2017). Pracaxi Impairs General Activity and Locomotion in Male Mice. Pharmacology.

[29] Serra J.L., da Cruz Rodrigues A.M., Alves de Freitas R., de Almeida Meirelles A.J., Darnet S.H., Meller da Silva L.H. (2019). Alternative Sources of Oils and Fats from Amazonian Plants: Fatty Acids, Methyl Tocols, Total Carotenoids and Chemical Composition. Food Res. Int..

[30] Chai W., Liebman M. (2005). Oxalate Content of Legumes, Nuts, and Grain-Based Flours. J. Food Compos. Anal..

[31] Ritter M.M.C., Savage G.P. (2007). Soluble and Insoluble Oxalate Content of Nuts. J. Food Compos. Anal..

[32] Leyva-Jiménez F.J., Lozano-Sánchez J., Borrás-Linares I., Arráez-Román D., Segura-Carretero A. (2018). Comparative Study of Conventional and Pressurized Liquid Extraction for Recovering Bioactive Compounds from *Lippia citriodora* Leaves. Food Res. Int..

[33] Adams M., Gmünder F., Hamburger M. (2007). Plants Traditionally Used in Age Related Brain Disorders—A Survey of Ethnobotanical Literature. J. Ethnopharmacol..

[34] Suárez-Montenegro Z.J., Ballesteros-Vivas D., Gallego R., Valdés A., Sánchez-Martínez J.D., Parada-Alfonso F., Ibáñez E., Cifuentes A., Silva M.F. (2021). Neuroprotective Potential of Tamarillo (*Cyphomandra betacea*) Epicarp Extracts Obtained by Sustainable Extraction Process. Front. Nutr..

[35] Bezerra H.M. (2018). Avaliação da Atividade Antioxidante e Otimização das Condições de Microencapsulação Por Spray Drying do Extrato Seco do Subproduto Agro-Industrial do Pracaxi (*Pentaclethra macroloba* Willd.). Master’s Thesis.

[36] Silva da Costa R., Gabbay Alves V., Lopes da Silva R., de Meneses Costa M. (2021). Agro-Industrial By-Products from Amazonian Fruits: Use for Obtaining Bioproducts. Bioactive Compounds in Nutraceutical and Functional Food for Good Human Health.

[37] Ambriz-Pérez D.L., Leyva-López N., Gutierrez-Grijalva E.P., Heredia J.B. (2016). Phenolic Compounds: Natural Alternative in Inflammation Treatment. A Review. Cogent Food Agric..

[38] Ribeiro D., Freitas M., Tomé S.M., Silva A.M.S., Porto G., Cabrita E.J., Marques M.M.B., Fernandes E. (2014). Inhibition of LOX by Flavonoids: A Structure-Activity Relationship Study. Eur. J. Med. Chem..

[39] Narváez-Cuenca C.E., Vincken J.P., Gruppen H. (2012). Identification and Quantification of (Dihydro) Hydroxycinnamic Acids and Their Conjugates in Potato by UHPLC-DAD-ESI-MSn. Food Chem..

[40] Li Z., Zhao C., Zhao X., Xia Y., Sun X., Xie W., Ye Y., Lu X., Xu G. (2018). Deep Annotation of Hydroxycinnamic Acid Amides in Plants Based on Ultra-High-Performance Liquid Chromatography-High-Resolution Mass Spectrometry and Its in Silico Database. Anal. Chem..

[41] Parr A.J., Mellon F.A., Colquhoun I.J., Davies H.V. (2005). Dihydrocaffeoyl Polyamines (Kukoamine and Allies) in Potato (*Solanum tuberosum*) Tubers Detected during Metabolite Profiling. J. Agric. Food Chem..

[42] Viana F.A., Braz-Filho R., Pouliquen Y.B.M., Andrade Neto M., Santiago G.M.P., Rodrigues-Filho E. (2004). Triterpenoid Saponins from Stem Bark of *Pentaclethra macroloba*. J. Braz. Chem. Soc..

[43] Rezgui A., Mitaine-Offer A.C., Miyamoto T., Tanaka C., Delemasure S., Dutartre P., Lacaille-Dubois M.A. (2016). Oleanolic Acid and Hederagenin Glycosides from *Weigela stelzneri*. Phytochemistry.

[44] Yao Y., Yang X., Shi Z., Ren G. (2014). Anti-Inflammatory Activity of Saponins from Quinoa (*Chenopodium quinoa* Willd.) Seeds in Lipopolysaccharide-Stimulated RAW 264.7 Macrophages Cells. J. Food Sci..

