# Supplementary material for: A Comprehensive Study on the Chemical Characterization and Neuroprotective Evaluation of Pracaxi Nuts Extracts Obtained by a Sustainable Approach
Source: Foods. 2023 Oct 23;12(20):3879. doi: 10.3390/foods12203879 (PMC10606833; doi:10.3390/foods12203879)
Supplement: Supplementary file 1 [file foods-12-03879-s001.zip › foods-2669879-supplementary.pdf]

foods
MDPI

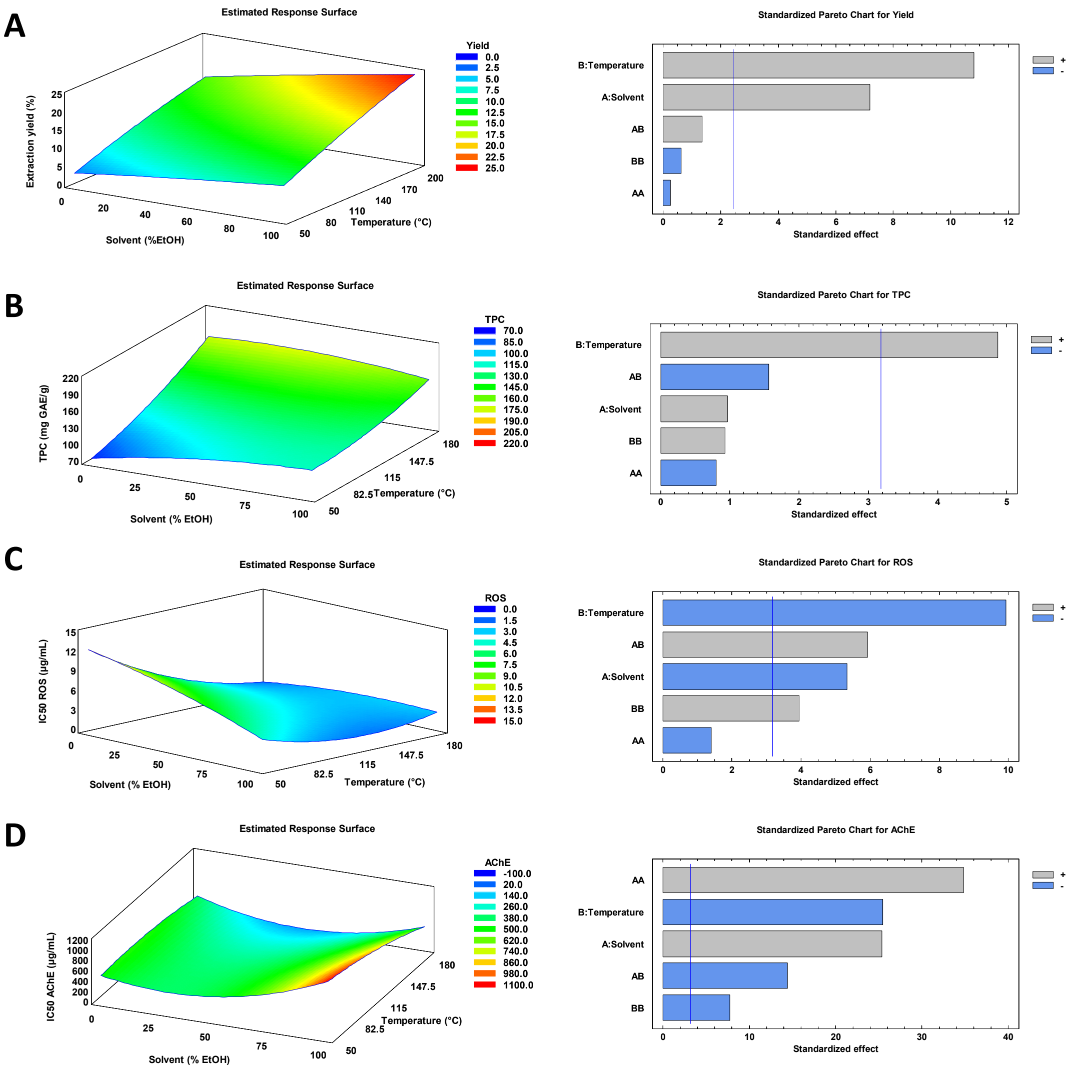

Figure S1. Estimated response surfaces for each response variable, and their corresponding Standardized Pareto charts: **A**) Extraction yield (%); **B**) TPC (mg GAE/mL); **C**) IC_{50} ROS (µg/mL); **D**, IC_{50} AChE (µg/mL).

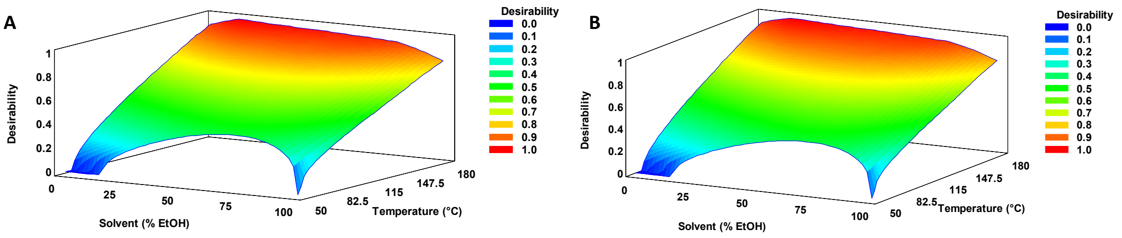

Figure S2. Desirability response surface to optimize response variables: **A**) including extraction yield as response variable; **B**) excluding extraction yield as response variable.

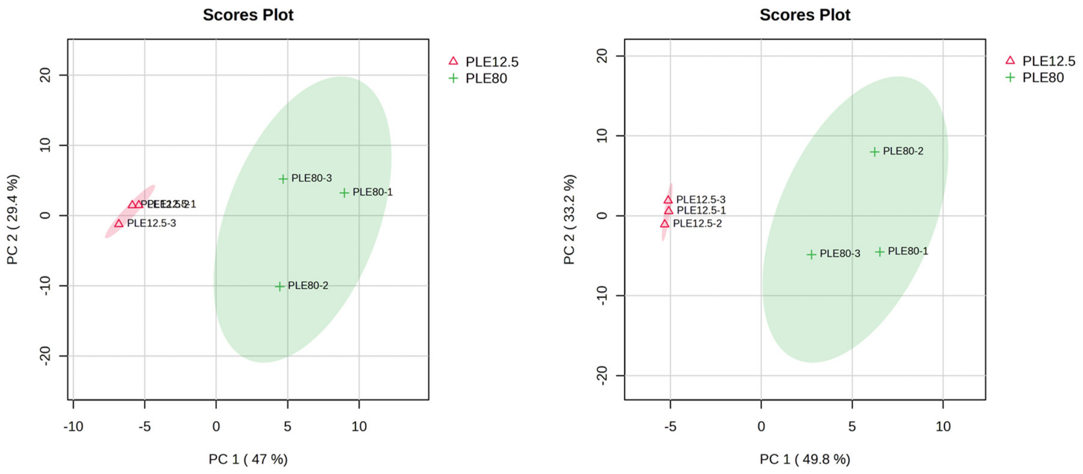

Figure S3. PCA score plots of pracaxi nuts PLE extracts data obtained by: **A**) HPLC-C18-Q-TOF MS/MS ESI (+); **B**) HPLC-C18-Q-TOF MS/MS ESI (–).

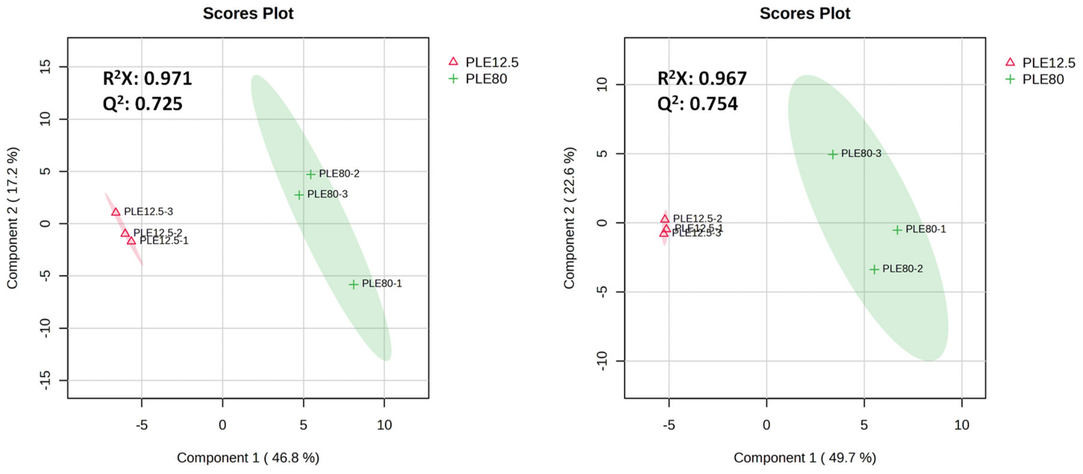

Figure S4. PLS-DA score plots of pracaxi nuts PLE extracts data obtained by: **A**) HPLC-C18-Q-TOF MS/MS ESI (+); **B**) HPLC-C18-Q-TOF MS/MS ESI (–).

Table S1. Analysis of Variance of extraction yield variable for response surface modeling showing linear, quadratic and interaction relations, and coefficient for model prediction.

Source	*Sum of Squares*	*Df*	*CE*		*F-Ratio*	*P-Value*
Model			β_0	−1.84655		
Solvent composition (% EtOH)	94.4067	1	β_1	0.0556026	90.56	0.0025*
Temperature (°C)	213.607	1	β_2	0.106144	204.9	0.0007*
Solvent composition^2	0.135	1	$\beta_{1,1}$	-9.00×10^{-5}	0.13	0.7428
Solvent composition x Temperature	3.4225	1	$\beta_{1,2}$	2.85×10^{-4}	3.28	0.1677
Temperature^2	0.735	1	$\beta_{2,2}$	-1.24×10^{-4}	0.71	0.4627
Lack-of-fit	7.84833	3			2.51	0.2349
Pure error	3.1275	3				
Total (corr.)	323.627	11				

Df (degree of freedom)

CE (coefficients of regression equation)

* Denotes statistical differences ($p < 0.05$)

R-squared = 96.6085 percent

R-squared (adjusted for d.f.) = 93.7822 percent

Standard Error of Est. = 1.02103

Mean absolute error = 0.888889

Durbin-Watson statistic = 2.14667 (P=0.5543)

Lag 1 residual autocorrelation = -0.189627

Asterisk indicates statistical significance (p-value < 0.05).

Table S2. Analysis of Variance of TPC variable for response surface modeling showing linear, quadratic and interaction relations, and coefficient for model prediction.

Source	*Sum of Squares*	*Df*	*CE*		*F-Ratio*	*P-Value*
Model			β_0	58.4568		
Solvent composition (% EtOH)	299.627	1	β_1	0.992372	0.93	0.4068
Temperature (°C)	7661.23	1	β_2	0.206933	23.69	0.0166*
Solvent composition^2	207.682	1	$\beta_{1,1}$	-3.53×10^{-3}	0.64	0.4816
Solvent composition x Temperature	792.422	1	$\beta_{1,2}$	-4.33×10^{-3}	2.45	0.2155
Temperature^2	281.535	1	$\beta_{2,2}$	$-.43 \times 10^{-3}$	0.87	0.4197
Lack-of-fit	379.968	3			0.39	0.7692
Pure error	970.388	3				
Total (corr.)	10472.6	11				

Df (degree of freedom)

CE (coefficients of regression equation)

* Denotes statistical differences ($p < 0.05$)

R-squared = 87.1059 percent

R-squared (adjusted for d.f.) = 76.3608 percent

Standard Error of Est. = 17.9851

Mean absolute error = 8.39028

Durbin-Watson statistic = 1.96499 (P=0.3927)

Lag 1 residual autocorrelation = -0.0617368

Asterisk indicates statistical significance (p-value < 0.05)

Table S3. Analysis of Variance of ROS variable for response surface modeling showing linear, quadratic and interaction relations, and coefficient for model prediction.

Source	*Sum of Squares*	*Df*	*CE*		*F-Ratio*	*P-Value*
Model			β_0	20.5195		
Solvent composition (% EtOH)	16.9277	1	β_1	−0.0880672	28.37	0.0129*
Temperature (°C)	58.7251	1	β_2	−0.184977	98.43	0.0022*
Solvent composition^2	1.16998	1	$\beta_{1,1}$	-2.65×10^{-4}	1.96	0.2559
Solvent composition x Temperature	20.9444	1	$\beta_{1,2}$	7.04×10^{-4}	35.1	0.0096*
Temperature^2	9.2964	1	$\beta_{2,2}$	4.42×10^{-4}	15.58	0.029*
Lack-of-fit	11.3316	3			6.33	0.0819
Pure error	1.78989	3				
Total (corr.)	119.02	11				

Df (degree of freedom)

CE (coefficients of regression equation)

* Denotes statistical differences ($p < 0.05$)

R-squared = 88.9754 percent

R-squared (adjusted for d.f.) = 79.7882 percent

Standard Error of Est. = 0.772419

Mean absolute error = 0.876167

Durbin-Watson statistic = 2.3977 (P=0.7609)

Lag 1 residual autocorrelation = -0.234766

Asterisk indicates statistical significance (p-value < 0.05)

Table S4. Analysis of Variance of AChE variable for response surface modeling showing linear, quadratic and interaction relations, and coefficient for model prediction.

Source	*Sum of Squares*	*Df*	*CE*		*F-Ratio*	*P-Value*
Model			β_0	366.498		
Solvent composition (% EtOH)	176474	1	β_1	−6.48654	641.92	0.0001*
Temperature (°C)	178193	1	β_2	3.48097	648.17	0.0001*
Solvent composition^2	333468	1	$\beta_{1,1}$	1.41×10^{-1}	1212.98	0.0001*
Solvent composition x Temperature	57121	1	$\beta_{1,2}$	-3.68×10^{-2}	207.78	0.0007*
Temperature^2	16590	1	$\beta_{2,2}$	-1.87×10^{-2}	60.35	0.0044*
Lack-of-fit	123530	3			149.78	0.0009*
Pure error	824.75	3				
Total (corr.)	874174	11				

Df (degree of freedom)

CE (coefficients of regression equation)

* Denotes statistical differences ($p < 0.05$)

R-squared = 85.7746 percent

R-squared (adjusted for d.f.) = 73.92 percent

Standard Error of Est. = 16.5806

Mean absolute error = 77.4722

Durbin-Watson statistic = 2.00322 (P=0.4261)

Lag 1 residual autocorrelation = -0.00284093

Asterisk indicates statistical significance (p-value < 0.05)

Table S5. Annotated compounds and their total compound contribution (%) in pracaxi SFE extract after HPLC-CSH-Q-TOF MS/MS ESI (-) analysis.

Average Mz	*Average RT (min)*	*Metabolite name*	*Adduct type*	*MSI Level*	*MSI Level*	*Molecular Formula*	*Compound subclass*	*Total compound contribution (%)*
281.24976	2.73	**FA 18:1; (oleic acid)**	[M-H]-	1	*m/z*, MS/MS, RT	C18H34O2	Fatty acids and conjugates	37.81
339.32681	4.59	**FA 22:0; (behenic acid)**	[M-H]-	1	*m/z*, MS/MS, RT	C22H44O2	Fatty acids and conjugates	14.28
279.23251	2.07	**FA 18:2; (linoleic acid)**	[M-H]-	1	*m/z*, MS/MS, RT	C18H32O2	Linoleic acids and derivatives	11.61
367.35602	5.31	**FA 24:0; (lignoceric acid)**	[M-H]-	1	*m/z*, MS/MS, RT	C24H48O2	Fatty acids and conjugates	10.43
283.26358	3.34	**FA 18:0; (stearic acid)**	[M-H]-	1	*m/z*, MS/MS, RT	C18H36O2	Fatty acids and conjugates	9.85
255.23288	2.51	**FA 16:0; (palmitic acid)**	[M-H]-	1	*m/z*, MS/MS, RT	C16H32O2	Fatty acids and conjugates	8.92
309.27914	3.42	**FA 20:1; (eicosenoic acid)**	[M-H]-	1	*m/z*, MS/MS, RT	C20H38O2	Fatty acids and conjugates	1.76
337.30914	3.98	**FA 22:1; (erucic acid)**	[M-H]-	1	*m/z*, MS/MS, RT	C22H42O2	Fatty acids and conjugates	1.64
311.29413	3.94	**FA 20:0; (arachidic acid)**	[M-H]-	1	*m/z*, MS/MS, RT	C20H40O2	Fatty acids and conjugates	1.02
395.39133	6.12	**FA 26:0; (cerotic acid)**	[M-H]-	1	*m/z*, MS/MS, RT	C26H52O2	Fatty acids and conjugates	0.89
365.34146	4.61	**FA 24:1; (nervonic acid)**	[M-H]-	1	*m/z*, MS/MS, RT	C24H46O2	Fatty acids and conjugates	0.35
253.21912	1.86	**FA 16:1; (palmitoleic acid)**	[M-H]-	1	*m/z*, MS/MS, RT	C16H30O2	Fatty acids and conjugates	0.29
353.34186	4.96	**FA 23:0**	[M-H]-	2b	*m/z*, MS/MS	C23H46O2	Fatty acids and conjugates	0.18
277.21735	1.58	**FA 18:3; (linolenic acid)**	[M-H]-	1	*m/z*, MS/MS, RT	C18H30O2	Linoleic acids and derivatives	0.16
227.20163	1.63	**FA 14:0; (myristic acid)**	[M-H]-	1	*m/z*, MS/MS, RT	C14H28O2	Fatty acids and conjugates	0.15
269.24841	3.00	**FA 17:0; (margaric acid)**	[M-H]-	1	*m/z*, MS/MS, RT	C17H34O2	Fatty acids and conjugates	0.14
381.37344	5.72	**FA 25:0**	[M-H]-	2b	*m/z*, MS/MS	C25H50O2	Fatty acids and conjugates	0.09
295.22772	0.82	**FA 18:2;O**	[M-H]-	2b	*m/z*, MS/MS	C18H32O3	Linoleic acids and derivatives	0.06
241.21724	2.04	**FA 15:0; (pentadecylic acid)**	[M-H]-	2a	*m/z*, RT	C15H30O2	Fatty acids and conjugates	0.05
267.23019	2.25	**FA 17:1**	[M-H]-	2b	*m/z*, MS/MS	C17H32O2	Fatty acids and conjugates	0.04
325.31213	4.26	**FA 21:0**	[M-H]-	2b	*m/z*, MS/MS	C21H42O2	Fatty acids and conjugates	0.04
439.35886	4.17	**FA 30:6**	[M-H]-	2b	*m/z*, MS/MS	C30H48O2	Fatty acids and conjugates	0.04
225.18394	1.22	**FA 14:1; (physeteric acid)**	[M-H]-	1	*m/z*, MS/MS, RT	C14H26O2	Fatty acids and conjugates	0.04
199.16656	1.05	**FA 12:0; (lauric acid)**	[M-H]-	2a	*m/z*, RT	C12H24O2	Fatty acids and conjugates	0.04
574.49921	6.07	**Ceramide d34:0**	[M+Cl]-	2a	*m/z*, RT	C34H69NO3	Ceramides	0.03
307.2627	2.95	**FA 20:2; (eicosadienoic acid)**	[M-H]-	1	*m/z*, MS/MS, RT	C20H36O2	Fatty acids and conjugates	0.02
415.35745	5.07	**FA 28:4**	[M-H]-	2b	*m/z*, MS/MS	C28H48O2	Fatty acids and conjugates	0.02
251.19859	1.37	**FA 16:2**	[M-H]-	2b	*m/z*, MS/MS	C16H28O2	Fatty acids and conjugates	0.02
335.2966	3.54	**FA 22:2; (docosadienoic acid)**	[M-H]-	2a	*m/z*, RT	C22H40O2	Fatty acids and conjugates	0.01
143.10736	0.57	**FA 8:0; (caprylic acid)**	[M-H]-	2a	*m/z*, RT	C8H16O2	Fatty acids and conjugates	0.01

Table S6. Annotated compounds and their total compound contribution (%) in pracaxi SFE extract after HPLC-CSH-Q-TOF MS/MS ESI (+) analysis.

Average Mz	*Average RT (min)*	*Metabolite name*	*Adduct type*	*MSI Level*	*MSI Level*	*Molecular Formula*	*Compound subclass*	*Total compound contribution (%)*
923.7563_907.7833_902.8348	11.03	**TG 54:3|TG 18:1_18:1_18:1**	[M+K]+_[M+Na]+_[M+NH4]+	1	m/z, MS/MS, RT	C57H104O6	Triacylglycerol	10.73
979.8079_963.8411_958.8925	11.81	**TG 58:3|TG 22:0_18:1_18:2**	[M+K]+_[M+Na]+_[M+NH4]+	1	m/z, MS/MS, RT	C61H112O6	Triacylglycerol	10.50
977.7941_961.8201_956.8676	11.53	**TG 58:4|TG 18:1_22:1_18:2**	[M+K]+_[M+Na]+_[M+NH4]+	1	m/z, MS/MS, RT	C61H110O6	Triacylglycerol	8.60
930.8611	11.41	**TG 56:3|TG 18:1_18:1_20:1**	[M+NH4]+	2a	m/z, RT	C59H108O6	Triacylglycerol	7.05
898.7927_903.7485	10.31	**TG 54:5|TG 18:1_18:2_18:2**	[M+NH4]+_[M+Na]+	1	m/z, MS/MS, RT	C57H100O6	Triacylglycerol	6.90
960.9102_965.8542_981.8278	11.98	**TG 58:2|TG 22:0_18:1_18:1**	[M+NH4]+_[M+Na]+_[M+K]+	1	m/z, MS/MS, RT	C61H114O6	Triacylglycerol	6.43
921.7391_905.7672_900.8184	10.69	**TG 54:4|TG 18:1_18:1_18:2**	[M+K]+_[M+Na]+_[M+NH4]+	1	m/z, MS/MS, RT	C57H102O6	Triacylglycerol	6.00
986.917	12.03	**TG 60:3|TG 24:0_18:1_18:2**	[M+NH4]+	2b	m/z, MS/MS	C63H116O6	Triacylglycerol	5.33
993.8839_1009.8556_988.9398	12.13	**TG 60:2|TG 24:0_18:1_18:1**	[M+Na]+_[M+K]+_[M+NH4]+	1	m/z, MS/MS, RT	C63H118O6	Triacylglycerol	4.31
932.8668_953.7968	11.75	**TG 56:2|TG 20:0_18:1_18:1**	[M+NH4]+_[M+K]+	1	m/z, MS/MS, RT	C59H110O6	Triacylglycerol	4.24
933.7853_949.7598_928.8427	11.10	**TG 56:4|TG 18:1_20:1_18:2**	[M+Na]+_[M+K]+_[M+NH4]+	1	m/z, MS/MS, RT	C59H106O6	Triacylglycerol	3.55
881.7598_897.7347_876.8074	10.99	**TG 52:2|TG 16:0_18:1_18:1**	[M+Na]+_[M+K]+_[M+NH4]+	1	m/z, MS/MS, RT	C55H102O6	Triacylglycerol	3.37
984.8951_1005.8330	11.87	**TG 60:4|TG 18:1_24:1_18:2**	[M+NH4]+_[M+K]+	1	m/z, MS/MS, RT	C63H114O6	Triacylglycerol	2.68
909.8022_925.7759_904.8306	11.42	**TG 54:2|TG 18:0_18:1_18:1**	[M+Na]+_[M+K]+_[M+NH4]+	1	m/z, MS/MS, RT	C57H106O6	Triacylglycerol	1.93
1037.889_1021.9243_1016.9614	12.22	**TG 62:2**	[M+K]+_[M+Na]+_[M+NH4]+	2a	m/z, RT	C65H122O6	Triacylglycerol	1.65
659.5071_643.5297_638.5728	7.12	**DG 36:2|DG 18:1_18:1**	[M+K]+_[M+Na]+_[M+NH4]+	1	m/z, MS/MS, RT	C39H72O5	Diacylglycerols	1.53
872.7745	10.29	**TG 52:4|TG 16:1_18:1_18:2**	[M+NH4]+	1	m/z, MS/MS, RT	C55H98O6	Triacylglycerol	1.22

895.7213_879.7471_874.7914	10.63	**TG 52:3|TG 16:0_18:1_18:2**	[M+K]+_[M+Na]+_[M+NH4]+	1	m/z, MS/MS, RT	C55H100O6	Triacylglycerol	1.20
917.7013_901.7272_896.7706	9.93	**TG 54:6|TG 18:2_18:2_18:2**	[M+K]+_[M+Na]+_[M+NH4]+	1	m/z, MS/MS, RT	C57H98O6	Triacylglycerol	1.15
657.4871_641.5132_636.5561	6.58	**DG 36:3|DG 18:1_18:2**	[M+K]+_[M+Na]+_[M+NH4]+	1	m/z, MS/MS, RT	C39H70O5	Diacylglycerols	0.87
959.8052_954.8490_975.7749	11.16	**TG 58:5|TG 22:1_18:2_18:2**	[M+Na]+_[M+NH4]+_[M+K]+	1	m/z, MS/MS, RT	C61H108O6	Triacylglycerol	0.81
947.7493_931.7764_926.8237	10.74	**TG 56:5|TG 20:1_18:2_18:2**	[M+K]+_[M+Na]+_[M+NH4]+	1	m/z, MS/MS, RT	C59H104O6	Triacylglycerol	0.76
974.9140_979.8685	12.08	**TG 59:2**	[M+NH4]+_[M+Na]+	2a	m/z, RT	C62H116O6	Triacylglycerol	0.66
878.8253	11.40	**TG 52:1|TG 16:0_18:0_18:1**	[M+NH4]+	1	m/z, MS/MS, RT	C55H104O6	Triacylglycerol	0.65
993.8309_977.8547_972.8976	11.93	**TG 59:3|TG 23:0_18:1_18:2**	[M+K]+_[M+Na]+_[M+NH4]+	1	m/z, MS/MS, RT	C62H114O6	Triacylglycerol	0.58
962.9131	12.14	**TG 58:1**	[M+NH4]+	2a	m/z, RT	C61H116O6	Triacylglycerol	0.47
906.8511_927.7825	11.74	**TG 54:1|TG 18:0_18:0_18:1**	[M+NH4]+_[M+K]+	1	m/z, MS/MS, RT	C57H108O6	Triacylglycerol	0.44
911.7518_895.7775_890.8185	11.23	**TG 53:2|TG 17:0_18:1_18:1**	[M+K]+_[M+Na]+_[M+NH4]+	1	m/z, MS/MS, RT	C56H104O6	Triacylglycerol	0.44
934.8787	12.01	**TG 56:1|TG 16:0_22:0_18:1**	[M+NH4]+	2b	m/z, MS/MS	C59H112O6	Triacylglycerol	0.42
967.8142_951.8406_946.8867	11.92	**TG 57:2|TG 21:0_18:1_18:1**	[M+K]+_[M+Na]+_[M+NH4]+	1	m/z, MS/MS, RT	C60H112O6	Triacylglycerol	0.41
1014.9418_1035.8682	12.16	**TG 62:3**	[M+NH4]+_[M+K]+	2a	m/z, RT	C65H120O6	Triacylglycerol	0.39
696.6494_701.6060	8.97	**DG 40:1|DG 22:0_18:1**	[M+NH4]+_[M+Na]+	2b	m/z, MS/MS	C43H82O5	Diacylglycerols	0.38
853.7298_869.7048_848.7749	10.61	**TG 50:2|TG 16:0_16:1_18:1**	[M+Na]+_[M+K]+_[M+NH4]+	1	m/z, MS/MS, RT	C53H98O6	Triacylglycerol	0.34
851.7112_867.6879_846.7594	10.25	**TG 50:3 A**	[M+Na]+_[M+K]+_[M+NH4]+	2a	m/z, RT	C53H96O6	Triacylglycerol	0.30
976.8957	11.12	**TG 58:2;1O|TG 22:0_18:1_18:1;1O**	[M+NH4]+	2b	m/z, MS/MS	C61H114O7	Oxidized triglyceride	0.25
855.7427_850.7925	10.99	**TG 50:1|TG 16:0_16:0_18:1**	[M+Na]+_[M+NH4]+	1	m/z, MS/MS, RT	C53H100O6	Triacylglycerol	0.25
909.7307_893.7579_888.8032	10.86	**TG 53:3|TG 17:0_18:1_18:2**	[M+K]+_[M+Na]+_[M+NH4]+	1	m/z, MS/MS, RT	C56H102O6	Triacylglycerol	0.23

724.6851_729.6406	9.53	**DG 42:1|DG 24:0_18:1**	[M+NH4]+_[M+Na]+	2b	m/z, MS/MS	C45H86O5	Diacylglycerols	0.18
937.764_921.7919_916.8352	11.23	**TG 55:3|TG 18:1_18:1_19:1**	[M+K]+_[M+Na]+_[M+NH4]+	1	m/z, MS/MS, RT	C58H106O6	Triacylglycerol	0.17
918.8124	9.87	**TG 54:3;1O|TG 18:1_18:1_18:1;1O**	[M+NH4]+	2b	m/z, MS/MS	C57H104O7	Oxidized triglyceride	0.15
972.8616	10.38	**TG 58:4;1O|TG 18:1_18:2_22:1;1O**	[M+NH4]+	2b	m/z, MS/MS	C61H110O7	Oxidized triglyceride	0.14
633.4877_617.5115_612.5547	7.06	**DG 34:1|DG 16:0_18:1**	[M+K]+_[M+Na]+_[M+NH4]+	1	m/z, MS/MS, RT	C37H70O5	Diacylglycerols	0.14
655.472_639.4988_634.5423	6.04	**DG 36:4|DG 18:2_18:2**	[M+K]+_[M+Na]+_[M+NH4]+	1	m/z, MS/MS, RT	C39H68O5	Diacylglycerols	0.14
944.867	11.63	**TG 57:3|TG 21:0_18:1_18:2**	[M+NH4]+	2b	m/z, MS/MS	C60H110O6	Triacylglycerol	0.13
891.6811_875.7121_870.7532	9.90	**TG 52:5|TG 18:1_16:2_18:2**	[M+K]+_[M+Na]+_[M+NH4]+	1	m/z, MS/MS, RT	C55H96O7	Triacylglycerol	0.12
661.5183_645.5452_640.5891	7.73	**DG 36:1|DG 18:0_18:1**	[M+K]+_[M+Na]+_[M+NH4]+	1	m/z, MS/MS, RT	C39H74O5	Diacylglycerols	0.11
974.8779	10.74	**TG 58:3;1O|TG 18:1_18:1_22:1;1O**	[M+NH4]+	2b	m/z, MS/MS	C61H112O7	Oxidized triglyceride	0.10
844.7417	9.90	**TG 50:4|TG 14:1_18:1_18:2**	[M+NH4]+	2b	m/z, MS/MS	C53H94O6	Triacylglycerol	0.09
970.8812	11.69	**TG 59:4|TG 23:0_18:2_18:2**	[M+NH4]+	2b	m/z, MS/MS	C62H112O6	Triacylglycerol	0.09
923.8068_918.8463	11.60	**TG 55:2|TG 18:0_17:1_20:1**	[M+Na]+_[M+NH4]+	1	m/z, MS/MS, RT	C58H108O6	Triacylglycerol	0.08
1012.926	12.06	**TG 62:4|TG 26:0_18:2_18:2**	[M+NH4]+	1	m/z, MS/MS, RT	C65H118O6	Triacylglycerol	0.08
982.8835	11.49	**TG 60:5**	[M+NH4]+	2a	m/z, RT	C63H112O6	Triacylglycerol	0.07
886.7846_907.7150	10.49	**TG 53:4|TG 17:1_18:1_18:2**	[M+NH4]+_[M+K]+	1	m/z, MS/MS, RT	C56H100O6	Triacylglycerol	0.06
722.6694_743.6091	9.09	**DG 42:2|DG 24:0_18:2**	[M+NH4]+_[M+K]+	2b	m/z, MS/MS	C45H84O5	Diacylglycerols	0.06
915.684_899.7114_894.7565	9.54	**TG 54:7|TG 18:2_18:2_18:3**	[M+K]+_[M+Na]+_[M+NH4]+	1	m/z, MS/MS, RT	C57H96O6	Triacylglycerol	0.06
631.476_615.4995_610.5392	6.52	**DG 34:2|DG 16:0_18:2**	[M+K]+_[M+Na]+_[M+NH4]+	1	m/z, MS/MS, RT	C37H68O5	Diacylglycerols	0.06
843.6837_827.7095_822.7546	10.59	**TG 48:1|TG 14:0_16:0_18:1**	[M+K]+_[M+Na]+_[M+NH4]+	1	m/z, MS/MS, RT	C51H96O6	Triacylglycerol	0.06
916.7979	9.46	**TG 54:4;1O|TG 18:1_18:2_18:1;1O**	[M+NH4]+	2b	m/z, MS/MS	C57H102O7	Oxidized triglyceride	0.05
666.6044_671.5598	7.74	**DG 38:2|DG 18:1_20:1**	[M+NH4]+_[M+Na]+	2b	m/z, MS/MS	C41H76O5	Diacylglycerols	0.05

841.6709_825.6935_820.7418	10.18	**TG 48:2**	[M+K]+_[M+Na]+_[M+NH4]+	2a	m/z, RT	C51H94O6	Triacylglycerol	0.05
914.8154	10.89	**TG 55:4|TG 18:1_19:1_18:2**	[M+NH4]+	2b	m/z, MS/MS	C58H104O6	Triacylglycerol	0.05
818.7216	9.90	**TG 48:3|TG 14:0_16:1_18:2**	[M+NH4]+	2b	m/z, MS/MS	C51H92O6	Triacylglycerol	0.04
1002.9177	11.36	**TG 60:3;1O|TG 18:1_20:1_22:1;1O**	[M+NH4]+	2b	m/z, MS/MS	C63H116O7	Oxidized triglyceride	0.04
948.8986	12.08	**TG 57:1**	[M+NH4]+	2a	m/z, RT	C60H114O6	Triacylglycerol	0.04
1042.9739	12.23	**TG 64:3**	[M+NH4]+	2a	m/z, RT	C67H124O6	Triacylglycerol	0.04
883.7145_867.7424_862.7841	10.84	**TG 51:2|TG 16:0_17:1_18:1**	[M+K]+_[M+Na]+_[M+NH4]+	1	m/z, MS/MS, RT	C54H100O6	Triacylglycerol	0.04
860.7714	10.51	**TG 51:3|TG 15:1_18:1_18:1**	[M+NH4]+	2b	m/z, MS/MS	C54H98O6	Triacylglycerol	0.04
892.8317	11.58	**TG 53:1|TG 17:0_18:0_18:1**	[M+NH4]+	1	m/z, MS/MS, RT	C56H106O6	Triacylglycerol	0.04
864.8019	11.22	**TG 51:1|TG 16:0_17:0_18:1**	[M+NH4]+	1	m/z, MS/MS, RT	C54H102O6	Triacylglycerol	0.04
694.6366	8.35	**DG 40:2|DG 18:1_22:1**	[M+NH4]+	2b	m/z, MS/MS	C43H80O5	Diacylglycerols	0.02
668.6227_673.5733	8.38	**DG 38:1|DG 20:0_18:1**	[M+NH4]+_[M+Na]+	2b	m/z, MS/MS	C41H78O5	Diacylglycerols	0.02
946.8414	9.54	**TG 56:3;1O|TG 20:1_18:2_18:0;1O**	[M+NH4]+	2b	m/z, MS/MS	C59H108O7	Oxidized triglyceride	0.02
920.8729	11.91	**TG 55:1**	[M+NH4]+	2a	m/z, RT	C58H110O6	Triacylglycerol	0.02
970.8424	10.03	**TG 58:5;1O|TG 18:2_18:2_22:1;1O**	[M+NH4]+	2b	m/z, MS/MS	C61H108O7	Oxidized triglyceride	0.02
842.7237	9.50	**TG 50:5**	[M+NH4]+	2a	m/z, RT	C53H92O6	Triacylglycerol	0.02
914.7808	9.15	**TG 54:5;1O|TG 18:1_18:2_18:2;1O**	[M+NH4]+	2b	m/z, MS/MS	C57H100O7	Oxidized triglyceride	0.02
664.5866	7.22	**DG 38:3|DG 20:1_18:2**	[M+NH4]+	2b	m/z, MS/MS	C41H74O5	Diacylglycerols	0.02
1044.9919	12.30	**TG 64:2**	[M+NH4]+	2a	m/z, RT	C67H126O6	Triacylglycerol	0.02
964.9267	12.21	**TG 58:0**	[M+NH4]+	2a	m/z, RT	C61H118O6	Triacylglycerol	0.02
202.1894	0.45	**NAE 9:0**	[M+H]+	2b	m/z, MS/MS	C11H23NO2	Amines	0.02
818.7230_823.6804	9.75	**TG 48:3|TG 12:0_18:1_18:2**	[M+NH4]+_[M+Na]+	1	m/z, MS/MS, RT	C51H92O6	Triacylglycerol	0.02
794.7263_799.6751	10.16	**TG 46:1**	[M+NH4]+_[M+Na]+	2a	m/z, RT	C49H92O6	Triacylglycerol	0.02
952.8367	10.78	**TG 58:6**	[M+NH4]+	2a	m/z, RT	C61H106O6	Triacylglycerol	0.01
752.7138	10.04	**DG 44:1|DG 26:0_18:1**	[M+NH4]+	2b	m/z, MS/MS	C47H90O5	Diacylglycerols	0.01
836.7705	10.81	**TG 49:1**	[M+NH4]+	2a	m/z, RT	C52H98O6	Triacylglycerol	0.01
884.7700_889.7270	10.09	**TG 53:5**	[M+NH4]+_[M+Na]+	2a	m/z, RT	C56H98O6	Triacylglycerol	0.01
991.8675_1007.8397	11.95	**TG 60:3**	[M+Na]+_[M+K]+	2a	m/z, RT	C63H116O6	Triacylglycerol	0.01
692.6197	7.85	**DG 40:3|DG 22:1_18:2**	[M+NH4]+	2b	m/z, MS/MS	C43H78O5	Diacylglycerols	0.01

912.7636	8.73	**TG 54:6;1O|TG 18:2_18:2_18:2;1O**	[M+NH4]+	2b	m/z, MS/MS	C57H98O7	Oxidized triglyceride	0.01
374.3294	2.87	**MG 18:1**	[M+NH4]+	2b	m/z, MS/MS	C21H40O4	Monoacylglycerols	0.01
356.3564	4.13	**NAE 20:0**	[M+H]+	2b	m/z, MS/MS	C22H45NO2	Amines	0.01
608.5269	5.96	**DG 34:3**	[M+NH4]+	2a	m/z, RT	C37H66O5	Diacylglycerols	0.01
694.6561	10.94	**CE 20:2**	[M+NH4]+	2a	m/z, RT	C47H80O2	Steroid esters	< 0.01
856.7423	9.69	**TG 51:5**	[M+NH4]+	2a	m/z, RT	C54H94O6	Triacylglycerol	< 0.01
816.7056	9.34	**TG 48:4**	[M+NH4]+	2a	m/z, RT	C51H90O6	Triacylglycerol	< 0.01
851.7086	10.40	**TG 50:3 B**	[M+Na]+	2a	m/z, RT	C53H96O6	Triacylglycerol	< 0.01
632.5263	5.59	**DG 36:5**	[M+NH4]+	2a	m/z, RT	C39H66O5	Diacylglycerols	< 0.01

Table S7. Annotated compounds and their total compound contribution (%) in pracaxi SFE extract after GC-Q-TOF MS analysis.

Average RT (min)	*Metabolite name*	*MSI Level*	*MSI Level*	*Molecular Formula*	*Compound subclass*	*Total compound contribution (%)*
19.829	**Oleic acid**	1	MS, RT	C18H34O2	Fatty acids and conjugates	11.87
18.247	**Palmitic acid**	1	MS, RT	C16H32O2	Fatty acids and conjugates	11.34
20.054	**Stearic acid**	1	MS, RT	C18H36O2	Fatty acids and conjugates	6.94
9.355/9.572	**Glycerol**	1	MS, RT	C3H8O3	Carbohydrates and carbohydrate conjugates	6.63
24.122	**1-monooleoylglycerol**	1	MS, RT	C21H40O4	Monoacylglycerols	6.61
24.721	**Lignoceric acid**	1	MS, RT	C24H48O2	Fatty acids and conjugates	6.27
7.675	**Alanine, N-methyl-N-allyloxycarbonyl-, undecyl ester**	2	MS	C19H35NO4	Amino acids, peptides, and analogues	5.94
7.664	**2-Pyrrolidinone**	1	MS, RT	C4H7NO	Pyrrolidones	5.89
19.77	**Linoleic acid**	1	MS, RT	C18H32O2	Linoleic acids and derivatives	3.66
7.441/9.106	**Oxalic acid**	1	MS, RT	C2H2O4	Dicarboxylic acids and derivatives	3.29
16.58	**Tagatose**	2	MS	C6H12O6	Carbohydrates and carbohydrate conjugates	2.60
25.822	**gamma-tocopherol**	1	MS, RT	C28H48O2	Quinone and hydroquinone lipids	2.54
16.484	**Uridine**	2	MS	C9H12N2O6	Pyrimidine nucleosides	1.98
7.219/7.241	**Hydroxylamine**	1	MS, RT	H3NO	Homogeneous other non-metal compounds	1.45
9.887	**Methylmalonic acid**	2	MS	C4H6O4	Dicarboxylic acids and derivatives	1.23
9.905	**Succinic acid**	1	MS, RT	C4H6O4	Dicarboxylic acids and derivatives	0.96
24.585	**Squalene**	1	MS, RT	C30H50	Triterpenoids	0.82
27.695/28.082	**Stigmasterol**	2	MS	C29H48O	Stigmastanes and derivatives	0.78
11.103	**4-aminobutyric acid**	2	MS	C4H9NO2	Amino acids, peptides, and analogues	0.76
7.085	**2-methyl-5-aminobenzoxazole**	2	MS	C8H8N2O	Benzoxazoles	0.74
15.627	**D-(+)-Glucuronic acid .gamma.-lactone, tris(trimethylsilyl) ether, methyloxime (anti)**	2	MS	C16H35NO6 Si3	Gamma butyrolactones	0.69
16.482	**D-(-)-Fructose**	1	MS, RT	C6H12O6	Carbohydrates and carbohydrate conjugates	0.65
22.951	**Behenic acid**	2	MS	C22H44O2	Fatty acids and conjugates	0.64
6.274	**L-norvaline**	2	MS	C5H11NO2	Amino acids, peptides, and analogues	0.55
16.614/16.742	**Gluconolactone**	1	MS, RT	C6H10O6	Carbohydrates and carbohydrate conjugates	0.53
7.833	**3-hydroxybutyric acid**	1	MS, RT	C4H8O3	Beta hydroxy acids and derivatives	0.52
6.535	**Lactic acid**	1	MS, RT	C3H6O3	Alpha hydroxy acids and derivatives	0.49
10.307	**2-oxohexanoic acid**	2	MS	C6H10O3	Medium-chain keto acids and derivatives	0.47

26.763	**alpha-tocopherol**	1	MS, RT	C29H50O2	Quinone and hydroquinone lipids	0.45
22.175	**(Z)-Docos-9-enenitrile**	2	MS	C22H41N	Organic cyanides	0.42
17.82	**7-Tetradecenal, (Z)-**	2	MS	C14H26O	Fatty aldehydes	0.41
16.28	**Myristic acid**	2	MS	C14H28O2	Fatty acids and conjugates	0.39
24.341	**Undec-10-ynoic acid, undec-2-en-1-yl ester**	2	MS	C22H38O2	Fatty alcohol esters	0.38
17.13	**Sedoheptulose**	1	MS, RT	C7H14O7	Carbohydrates and carbohydrate conjugates	0.37
24.439	**Methyl 22-methyl-tetracosanoate**	2	MS	C26H52O2	Fatty acid esters	0.37
7.569	**p-dichlorobenzene**	2	MS	C6H4Cl2	Halobenzenes	0.36
22.857	**1-monopalmitin**	1	MS, RT	C19H38O4	Monoacylglycerols	0.35
28.629	**Cycloartenol**	2	MS	C30H5O	Cycloartanols and derivatives	0.34
18.783	**trans-13-Octadecenoic acid**	2	MS	C18H34O2	Fatty acids and conjugates	0.33
26.073	**Cerotic acid**	2	MS	C26H52O2	Fatty acids and conjugates	0.29
14.456	**D-xylulose**	2	MS	C5H10O5	Carbohydrates and carbohydrate conjugates	0.27
8.231	**D-(-)-Pantolactone**	2	MS	C6H10O3	Gamma butyrolactones	0.27
12.059	**5-Hydroxymaltol**	2	MS	C6H6O4	Pyranones and derivatives	0.27
26.91	**Propyl docosanoate**	2	MS	C25H50O2	Fatty acid esters	0.27
12.76	**L-Iditol**	2	MS	C6H14O6	Carbohydrates and carbohydrate conjugates	0.27
17.989	**Palmitelaidic acid**	1	MS, RT	C16H30O2	Fatty acids and conjugates	0.26
24.293	**Glycerol monostearate**	2	MS	C21H42O4	Monoacylglycerols	0.26
9.022	**Eupatoriochromene**	2	MS	C13H14O3	1-benzopyrans	0.26
16.665	**Andrographolide**	2	MS	C20H30O5	Gamma butyrolactones	0.24
10.444	**3,4-Dihydroxy-5-methyl-dihydrofuran-2-one**	2	MS	C5H8O4	Gamma butyrolactones	0.24
12.562	**L-pyroglutamic acid**	1	MS, RT	C5H7NO3	Amino acids, peptides, and analogues	0.24
27.297	**Benzoic acid, 2-heptafluorobutyryloxy-, tert.-butyldimethylsilyl ester**	2	MS	C17H19F7O4Si	Benzoic acids and derivatives	0.23
8.624	**1-methoxy-2-propanol**	2	MS	C4H10O2	Alcohols and polyols	0.23
11.105	**Ethanolamine**	2	MS	C2H7NO	Amines	0.23
15.223	**3,6-anhydro-D-galactose**	1	MS, RT	C6H10O5	Tetrahydrofurans	0.23
8.509	**3-hydroxyisovaleric acid**	1	MS, RT	C5H10O3	Fatty acids and conjugates	0.22
9.054	**Benzoic acid**	1	MS, RT	C7H6O2	Benzoic acids and derivatives	0.20
14.135	**Lauric acid**	1	MS, RT	C12H24O2	Fatty acids and conjugates	0.20
16.738	**Dihydroconiferylic acid**	2	MS	C10H12O4	Phenylpropanoic acids	0.20
10.694	**Erythronic acid lactone**	1	MS, RT	C4H6O4	Gamma butyrolactones	0.19
22.444	**Methyl behenate**	1	MS, RT	C23H46O2	Fatty acid esters	0.19

9.252	**Octanoic acid**	1	MS, RT	C8H16O2	Fatty acids and conjugates	0.19
8.894	**Urea**	1	MS, RT	CH4N2O	Ureas	0.18
24.958	**Benzene, 1,2-bis(9-borabicyclo[3.3.1]non-9-yloxymethyl)-**	2	MS	C24H36B2O2	Borinanes	0.16
18.972	**1,4-Benzenediamine, N-(1-methylethyl)-N'-phenyl-**	2	MS	C15H18N2	Amines	0.16
13.692	**Glutaconic acid**	2	MS	C5H6O4	Dicarboxylic acids and derivatives	0.16
23.862	**2-monoolein**	1	MS, RT	C21H40O4	Monoacylglycerols	0.16
28.103	**beta-sitosterol**	2	MS	C29H50O	Stigmastanes and derivatives	0.13
10.537	**Pelargonic acid**	1	MS, RT	C9H18O2	Fatty acids and conjugates	0.13
21.67	**1-Pentadecyne**	2	MS	C15H28	Acetylides	0.13
6.282	**1,3-propanediol**	1	MS, RT	C3H8O2	Alcohols and polyols	0.13
8.318	**Malonic acid**	1	MS, RT	C3H4O4	Dicarboxylic acids and derivatives	0.12
17.076	**Methyl palmitate**	1	MS, RT	C17H34O2	Fatty acid esters	0.12
18.675	**Ferulic acid**	1	MS, RT	C10H10O4	Hydroxycinnamic acids and derivatives	0.12
14.104	**D-(+)-Ribono-1,4-lactone**	2	MS	C5H8O5	Carbohydrates and carbohydrate conjugates	0.10
15.387	**Arabinofuranose**	2	MS	C5H10O5	Carbohydrates and carbohydrate conjugates	0.10
11.07	**5-hydroxymethylfurfural**	2	MS	C6H6O3	Carbonyl compounds	0.10
19.168	**Margaric acid**	1	MS, RT	C17H34O2	Fatty acids and conjugates	0.09
16.447	**Senecioic acid**	2	MS	C5H8O2	Fatty acids and conjugates	0.08
26.517	**nonacosane**	2	MS	C29H60	Alkanes	0.08
11.536	**Reticuline, 6'-methyl**	2	MS	C20H25NO4	Benzylisoquinolines	0.08
15.994	**1-Tetradecyne**	2	MS	C14H26	Acetylides	0.08
17.194/17.204	**Coniferyl alcohol**	2	MS	C10H12O3	Methoxyphenols	0.07
19.023	**Methyl stearate**	1	MS, RT	C19H38O2	Fatty acid esters	0.07
22.491	**alpha-Lactose**	1	MS, RT	C12H22O11	Carbohydrates and carbohydrate conjugates	0.07
23.221	**Sucrose**	1	MS, RT	C12H22O11	Carbohydrates and carbohydrate conjugates	0.07
15.547	**D-(-)-Ribofuranose**	2	MS	C5H10O5	Carbohydrates and carbohydrate conjugates	0.07
23.973	**Methyl lignocerate**	1	MS, RT	C25H50O2	Fatty acid esters	0.06
16.438	**Methyl-beta-D-galactopyranoside**	2	MS	C7H14O6	Carbohydrates and carbohydrate conjugates	0.06
8.86	**2-butyne-1,4-diol**	1	MS, RT	C4H6O2	Alcohols and polyols	0.06
13.314	**3-phenyllactic acid**	1	MS, RT	C9H10O3	Phenylpropanoic acids	0.05
25.154	**delta-tocopherol**	2	MS	C27H46O2	Quinone and hydroquinone lipids	0.04
18.719	**Methyl linoleate**	1	MS, RT	C19H34O2	Linoleic acids and derivatives	0.04
8.406	**Aniline**	1	MS, RT	C6H7N	Aniline and substituted anilines	0.04
17.209	**4-hydroxycinnamic acid**	1	MS, RT	C9H8O3	Hydroxycinnamic acids and derivatives	0.04

22.762	**Brassidic acid**	2	MS	C22H42O2	Fatty acids and conjugates	0.04
9.723	**2-phenylacetic acid**	2	MS	C8H8O2	Benzene and substituted derivatives	0.04
14.673	**1,6-Anhydro-beta-D-glucose**	1	MS, RT	C6H10O5	Oxepanes	0.03
15.934	**Citric acid**	1	MS, RT	C6H8O7	Tricarboxylic acids and derivatives	0.03
16.705	**Glucopyranose**	2	MS	C6H12O6	Carbohydrates and carbohydrate conjugates	0.03
17.282	**Pentadecanoic acid**	2	MS	C15H30O2	Fatty acids and conjugates	0.03
10.209	**Uracil**	1	MS, RT	C4H4N2O2	Pyrimidines and pyrimidine derivatives	0.03
17.555	**D-lyxose**	2	MS	C5H10O5	Carbohydrates and carbohydrate conjugates	0.03
9.298	**1-(2-Ethyl-[1,3]dithian-2-yl)-3-methyl-butan-1-ol**	2	MS	C11H22OS2	Dithioacetals	0.03
11.787	**Capric acid**	2	MS	C10H20O2	Fatty acids and conjugates	0.02
18.618	**Oleonitrile**	2	MS	C18H33N	Organic cyanides	0.02
15.478	**D-(-)-Tagatofuranose**	2	MS	C6H12O6	Carbohydrates and carbohydrate conjugates	0.02
22.308	**L-(+)-Rhamnopyranose**	2	MS	C6H12O5	Carbohydrates and carbohydrate conjugates	0.02
13.191	**Tyrosol**	1	MS, RT	C8H10O2	Tyrosols and derivatives	0.02
20.799	**Methyl arachidate**	1	MS, RT	C21H42O2	Fatty acid esters	0.01

Table S8. PLE conditions, desirability and predicted response values at the optimum conditions predicted by the model, and experimental response values for the selected optimum conditions.

	**Including Extraction yield as response variable**		**Excluding Extraction yield as response variable**	
	Predicted Optimum	*Experimental Optimum (80% EtOH at 180 °C)*	*Predicted Optimum*	*Experimental Optimum (12.5% ethanol at 180 °C)*
Temperature (°C)	180	180	180	180
Solvent composition (% EtOH)	80.2	80	12.6	12.5
**Desirability**	0.934953		1.0000	
**Response variable**				
Extraction yield	21.22	24.0 ± 3.0		
ROS	2.94	1.5 ±1.0	1.98798	1.6 ± 1.0
TPC	168.86	91.9 ± 0.9	176.595	103.9 ± 0.5
AChE	247.00	276 ± 17	245.516	315 ± 29

Table S9. Tentative identified compounds in pracaxi nuts PLE extracts after HPLC-C18-Q-TOF MS/MS ESI (+/-) analyses.

			ESI (+)				Peak area						ESI (-)				Peak area					
**Compound number**	**Tentative compound name**	**Molecular Formula**	**Retention time**	***m/z***	**Adduct type**	**MS/MS Spectra**	**PLE 12**	**PLE 12**	**PLE 12**	**PLE 80**	**PLE 80**	**PLE 80**	**Retention time**	***m/z***	**Adduct type**	**MS/MS Spectra**	**PLE 12**	**PLE 12**	**PLE 12**	**PLE 80**	**PLE 80**	**PLE 80**
**1**	Agmatine	C5H14N4	0.497	131.12921	[M+H]+	97.07, 72.08, 60.06, 55.05	202825	173328	186034	115397	275883	201386										
**2**	L-Arginine	C6H14N4O2	0.537	175.11766	[M+H]+	70.07, 60.06	465561	437133	326465	191129	458072	424698										
**3**	Serine	C3H7NO3											0.545	104.02676	[M-H]-	74.02	265868	289990	192206	121600	198663	225002
**4**	D-Asparagine	C4H8N2O3											0.552	131.04405	[M-H]-	114.02, 113.03, 96.00, 72.01, 71.03,	1132701	1144543	327167	351796	62949	1185871

																70.03, 58.03						
**5**	Threonine	C4H9NO3	0.554	120.06519	[M+H]+	74.06, 57.03, 56.05	163989	167612	142685	108232	234263	142659										
**6**	D-Arabinonic acid	C5H10O6											0.571	165.03163	[M-H]-	87.01, 75.01, 72.99, 71.01, 59.01	854608	908332	722748	343277	1268501	475827
**7**	Galactonic acid	C6H12O7											0.572	195.04193	[M-H]-	195.05, 129.02, 99.01, 89.02, 87.01, 85.03, 75.01, 59.01	609254	683445	607868	209021	1064417	429571
**8**	Choline cation	C5H14NO	0.585	104.09943	[M]+	104.11, 60.08, 59.07, 58.06	18116185	10788031	18639726	14669455	17519303	15326008										
**9**	D-Lyxose	C5H10O5											0.611	149.04587	[M-H]-	71.01, 59.01	251990	262958	142933	23510	41118	38061
**10**	Glycerophosphocholine	C8H20NO6P	0.616	258.10812	[M+H]+	258.11, 184.07, 125.00, 104.11	426875	429884	328050	150065	230162	161092										

**11**	L-Monomethyl arginine	C7H16N4O2	0.618	116.07200	[M+H-C2H7N3]+	116.07, 70.06	9749293	10711968	11965546	6977617	11671552	7971261											
**12**	4-O-.beta.-Galactopyranosyl-D-mannopyranose	C12H22O11	0.628	365.10776	[M+Na]+	365.10, 203.06, 185.04	127340	101456	159954	104812	94675	116973	0.640	341.11005	[M-H]-	179.06 **[M-H-C6H10O5]**-, 119.04, 101.03, 89.03, 71.01, 59.01	342931	289721	349753	322776	384794	333396
			0.640	360.14896	[M+NH4]+		1230101	1458245	1665220	1258028	1392939	1356875	0.634	377.08151	[M+Cl]-	377.08 **[M+Cl]**-, 341.11 **[M-H]**-, 179.06 **[M-H-C6H10O5]**-, 161.04, 143.03, 119.03, 101.02, 89.02, 71.01, 59.01	2818602	2830567	3112748	2526314	2949596	2542230

													0.642	387.11133	[M+FA-H]-	341.11 [**M-H**]-, 179.06 [**M-H-C6H10O5**]-, 161.04, 119.03, 101.03, 89.02, 71.01, 59.01	592698	494955	609133	538969	580302	581272
													0.638	683.22595	[2M-H]-	683.23 [**2M-H**]-, 341.11 [**M-H**]-, 179.06 [**M-H-C6H10O5**]-	582713	467626	568493	428822	640834	494127
**13**	Trigonelline	C7H7NO2	0.652	138.05556	[M+H]+	138.06, 94.06	8114883	8434544	9118724	6688703	6389769	7857900										
			0.648	275.10193	[2M+H]+	275.18, 138.06, 94.07	334839	374713	412408	301762	260385	448491										
**14**	3-Hydroxypyridine	C5H5NO	0.656	96.04308	[M+H]+	96.04, 78.03, 68.05	2615453	2680147	2458912	2697515	2601682	3529302										

**15**	5-Hydroxy-2-methylpyridine	C_6H_7NO	0.673	110.06235	[M+H]+	110.06, 95.04, 94.03, 82.06, 81.06, 80.05, 67.04, 65.04	1834270	2084313	1773971	1477776	1304996	1977798										
**16**	Betaine	$C_5H_{11}NO_2$	0.674	118.08470	[M+H]+	118.09, 59.07, 58.06	1170165	1415371	1552937	1259719	2516723	1219785										
**17**	Malic acid	$C_4H_6O_5$											0.677	133.01433	[M-H]-	133.01, 115.01, 89.02, 72.99, 71.01, 59.02	7494537	7820892	6948604	8207084	10510066	7461399
**18**	Cadaverine	$C_5H_{14}N_2$	0.678	86.09517	[M+H-NH3]+	86.10, 69.07	147777	145973	132977	438966	324936	386118										
**19**	His-Pro	$C_{11}H_{16}N_4O_3$	0.680	235.11913	[M+H-H2O]+	235.12, 207.13, 166.06, 162.10, 110.07, 82.05, 70.07	860358	1048104	950386	877044	1061739	920442										
**20**	N-Methyl-L-leucine	$C_7H_{15}NO_2$	0.681	100.11154	[M+H-CH2O2]+	100.11, 58.07	45485	56696	30593	2218926	1025087	1120199										

**21**	D-Pyroglutamic acid	C5H7NO3											0.690	128.03595	[M-H]-	128.04, 82.03	1007147	1276705	772122	1302551	842456	1017140
**22**	alpha-Cyclopropyl-3-pyridinemethanol	C9H11NO	0.692	150.09009	[M+H]+	150.10, 122.07, 109.06, 108.05, 80.05	2257912	2257895	1112018	4561667	242197	2658581										
**23**	3-Hydroxypicolinic acid	C6H5NO3											0.725	138.02138	[M-H]-	94.03, 66.03	51591	31482	37011	42228	102524	21800
**24**	Succinic acid	C4H6O4											1.083	117.02073	[M-H]-	116.93, 99.93	130593	42936	47833	24195	100539	33175
**25**	2-Amino-2-methylpentanoic acid	C6H13NO2	1.135	132.10019	[M+H]+	86.09, 69.07, 58.06, 57.06	810392	377416	274955	265551	759002	540876										
**26**	4-aminobutyrate	C4H9NO2	1.379	86.05895	[M+H-H2O]+	86.06, 69.03	321977	671015	294297	692603	621034	1986478										
**27**	Itaconic acid	C5H6O4											1.788	129.02115	[M-H]-	85.03	338319	436415	121223	288950	282017	278431
**28**	Adenosine	C10H13N5O4	1.886	268.10464	[M+H]+	268.10, 136.06	1794752	1747699	1943421	1291268	1614062	1246524										

**29**	Guanosine	C10H13N5O5	1.969	284.10040	[M+H]+	152.06, 135.03, 110.03	128317	285796	290238	66468	290765	262792										
**30**	N-(2,4-Dimethylphenyl)formamide	C9H11NO	2.003	150.09045	[M+H]+	150.09, 108.04	2161186	2815859	1013584	3294648	603305	3935062										
**31**	4-Methyl-1H-benzimidazole	C8H8N2	2.055	133.07463	[M+H]+	133.08, 118.05, 106.06, 79.06, 77.04, 65.04	243470	151979	134085	591959	337910	353223										
**32**	L-Phenylalanine	C9H11NO2	2.079	166.08527	[M+H]+	121.08, 120.08, 103.05, 93.07	1361133	951893	1858648	383720	1243020	691165										
**33**	1,3-Dimethyl-1H-pyrazole-4-carbaldehyde	C6H8N2O	2.361	125.06705	[M+H]+	125.07, 97.07, 66.03, 56.05	141294	64631	140942	68742	127574	189730										
**34**	5-Hydroxymethylfurfural	C6H6O3	2.650	127.03857	[M+H]+	127.04, 109.03	160829	104313	135202	329041	210415	153670										
**35**	Catechol	C6H6O2											2.661	109.02938	[M-H]-	109.03, 108.02, 91.02	40497	90603	32055	42943	20825	61937
**36**	5,6-Dimethylbenzimidazole	C9H10N2	2.712	147.09184	[M+H]+	147.09, 132.07, 131.06, 120.08	69655	82125	26380	106980	21046	146010										

**37**	L-Tryptophan	$C_{11}H_{12}N_2O_2$	2.846	205.09726	[M+H]+	188.07 [**M+H-NH3**]+, 170.06, 159.09, 146.06, 144.08, 132.08, 131.07, 118.06	813164	1102972	1310362	513464	2434678	136033										
			2.843	188.07277	[M+H-NH3]+	188.11 [**M+H-NH3**]+, 170.06, 146.06, 144.08, 143.07, 118.06, 91.05	219496	327150	102562	32538	720029	136655										
**38**	N-Acetyl-DL-valine	$C_7H_{13}NO_3$	3.014	114.09003	[M+H-CH2O2]+	114.09, 72.08, 55.06	979616	1226513	771989	781050	468688	2002037										
**39**	N-L-.gamma.-Glutamyl-L-leucine	$C_{11}H_{20}N_2O_5$	3.061	261.14438	[M+H]+	244.12, 198.11, 132.10, 130.05, 86.10, 84.04	1771902	549550	1828917	779884	2473871	1219493	2.903	259.13132	[M-H]-	241.12, 197.13, 130.08, 128.04	490800	477755	461486	268283	581832	378303
**40**	3,4-Dihydroxybenzaldehyde	$C_7H_6O_3$											3.033	137.02615	[M-H]-	137.02, 136.02, 119.01,	600277	653845	411216	186937	189561	288094

																109.03, 108.02, 81.03						
**41**	N-(3-(Aminomethyl)benzyl)acetamidine	C10H15N3	3.125	178.13219	[M+H]+	178.13, 161.11, 120.08	107747	63977	34556	135417	107163	113666										
**42**	Diethyl L-glutamate	C9H17NO4	3.164	158.08083	[M+H-C2H6O]+	84.04, 56.05	506282	1010470	768009	9650484	7983113	1280228										
**43**	N-N'-bis-(dihydrocaffeoyl) spermidine-monoglucoside (+C6H10O5)	C31H45N3O11	3.766	636.31165	[M+H]+	636.31 [**M+H**]+, 618.33 [**M+H-H2O**]+, 600.30 [**M+H-H2O-H2O**]+, 474.26 [**M+H-C6H10O5**]+, 457.23 [**M+H-C6H10O5-NH3**]+, 384.17, 293.19 [**M+H-C6H10O5-NH3-dihydrocaffeo**	224836	270356	557888	306094	1021863	527587	3.604	634.29742	[M-H]-	634.29 [**M-H**]-, 616.28 [**M-H-H2O**]-, 472.24 [**M-H-C6H10O5**]-	49000	36843	35207	175967	138176	94256

						**yl**]+, 222.11, 112.11, 72.08																
**44**	N-N'-bis-(dihydrocaffeoyl) spermidine isomer 1	C25H35N3O6	4.016	474.26465	[M+H]+	474.26 [**M+H**]+, 457.23 [**M+H-NH3**]+, 310.21 [**M+H-dihydrocaffeoyl**]+, 293.19 [**M+H-dihydrocaffeoyl-NH3**]+, 239.14, 222.11, 165.05, 123.04, 72.08	45725887	45637899	30667725	38774055	34464216	22409077	3.657	472.24356	[M-H]-	472.24 [**M-H**]-, 350.21 [**M-H-122**]-, 308.20 [**M-H-dihydrocaffeoyl**]-, 186.16 [**M-H-122-dihydrocaffeoyl**]-, 121.03	13866180	15538475	16727529	20478262	37768759	19594869
			4.016	496.24115	[M+Na]+	496.24 [**M+H+Na**]+	1042213	1088070	1018131	428472	896164	561891										
			4.013	947.51434	[2M+H]+	474.25 [**M+H**]+, 457.23 [**M+H-NH3**]+	4757175	4324405	6039431	5521635	9282672	5796803										
			4.007	969.49170	[2M+Na]+		191214	193510	484195	387584	366225	433901										

**45**	N-N'-bis-(dihydrocaffeoyl) spermidine-monoglucoside (+C5H8O4)	C30H43N3O10	4.096	606.30280	[M+H]+	606.30 [**M+H**]+, 588.29 [**M+H-H2O**]+, 570.28 [**M+H-H2O-H2O**]+, 552.26 [**M+H-H2O-H2O-H2O**]+, 474.25 [**M+H-C5H8O4**]+, 457.22 [**M+H-C5H8O4-NH3**]+, 388.22 [**M+H-H2O-H2O-H2O-H2O-dihydrocaffeoyl**]+, 370.20 [**M+H-H2O-H2O-H2O-H2O-dihydrocaffeoyl-H2O**]+, 293.18 [**M+H-C5H8O4-NH3-dihydrocaffeoyl**]+, 222.10, 204.11, 150.08	6511369	5132566	5503461	1815844	2327723	1640619	3.726	604.28595	[M-H]-	604.29 [**M-H**]-, 586.28 [**M-H-H2O**]-, 568.26 [**M-H-H2O-H2O**]-, 545.27, 514.25, 472.24 [**M-H-C5H8O4**]-, 350.21 [**M-H-C5H8O4-122**]-	542631	768687	541977	329814	190147	248694

**46**	N-N'-bis-(dihydrocaffeoyl) spermidine-conjugate (+CH2)	C26H37N3O6	4.148	488.27515	[M+H]+	488.27 [**M+H**]+, 457.22 [**M+H-CH5N**]+, 324.23 [**M+H-dihydrocaffeoyl**]+, 293.18 [**M+H-CH5N-dihydrocaffeoyl**]+, 222.11, 165.05, 123.04, 86.10	770970	682701	612988	2808117	1404490	2762141	3.763	486.25940	[M-H]-	486.26 [**M-H**]-, 364.22 [**M-H-122**]-, 322.21 [**M-H-dihydrocaffeoyl**]-, 200.18 [**M-H-122-dihydrocaffeoyl**]-, 121.03, 111.08	2813	8531	2437	32580	41266	46007
**47**	N-N'-bis-(dihydrocaffeoyl) spermidine isomer 2	C25H35N3O6	4.230	474.25964	[M+H]+	474.26 [**M+H**]+, 457.23 [**M+H-NH3**]+, 310.21 [**M+H-dihydrocaffeoyl**]+, 293.19 [**M+H-NH3-dihydrocaffeoyl**]+, 222.11, 165.05, 123.04, 72.08	1633119	2966524	3445652	4256568	17743803	5336670	3.891	472.24435	[M-H]-		157112	64742	192422	434132	241065	39267

**48**	N-coumaroyl-N'-dihydrocaffeoyl spermidine	C25H33N3O5	4.231	456.24939	[M+H]+	456.25 [**M+H**]+, 439.23 [**M+H-NH3**]+, 293.19 [**M+H-NH3-coumaroyl**]+, 236.13, 221.13, 165.05, 123.04, 112.11, 84.08, 72.08	565690	980932	775209	3055129	1457841	3513731	3.840	454.23425	[M-H]-	454.23 [**M-H**]-, 332.20 [**M-H-122**]-, 297.15, 249.20, 233.96, 192.02, 123.30, 95.06	4798	5257	2303	12960	4833	12107
**49**	N-caffeoyl-N'-dihydrocaffeoyl spermidine-conjugate (+C5H2O)	C30H35N3O7	4.242	550.25385	[M+H]+	550.25 [**M+H**]+, 455.22 [**M+H-C5H5NO**]+, 383.16, 342.13, 326.10, 285.08, 243.07, 222.11, 165.05, 112.11	6416241	6539164	2579735	38562	3898	108345	3.886	548.24036	[M-H]-	548.24 [**M-H**]-, 453.20 [**M-H-C5H5NO**]-, 412.19, 326.09, 175.03, 135.04	13543	17349	15386	1185	419	1071
**50**	N-caffeoyl-N'-dihydrocaffeoyl spermidine isomer 1	C25H33N3O6	4.303	472.24429	[M+H]+	472.24 [**M+H**]+, 457.23 [**M+H-NH**]+, 310.21	1472412	3366534	3644219	3209044	6815905	2782821	3.945	470.22711	[M-H]-	470.23 [**M-H**]-, 350.20 [**M-H-120**]-, 308.20 [**M-**	345633	370403	195947	476301	827668	412308

						[**M+H-caffeoyl**]+, 293.19 [**M+H-NH3-caffeoyl**]+, 222.11, 165.05, 163.04, 123.04, 72.08										**H-caffeoyl**]-, 186.16 [**M-H-caffeoyl-122**]-, 161.02						
**51**	N-N'-bis-(dihydrocaffeoyl) spermidine-monoglucoside (+C6H10O4)	C31H45N3O10	4.327	620.31561	[M+H]+	620.32 [**M+H**]+, 602.31 [**M+H-H2O**]+, 584.29 [**M+H-H2O-H2O**]+, 566.28 [**M+H-H2O-H2O-H2O**]+, 474.26 [**M+H-C6H10O4**]+, 457.23 [**M+H-C6H10O4-NH3**]+, 293.18 [**M+H-C6H10O4-NH3-dihydrocaffeo**	1681890	1180123	872216	292517	961291	264824										

							**yl**]+, 222.11, 164.11, 95.05															
**52**	N-N'-bis-(dihydrocaffeoyl) spermidine-conjugate (+C2H4)	C27H39N3O6	4.334	502.28925	[M+H]+	502.29 [**M+H**]+, 457.23 [**M+H-C2H7N**]+, 338.24 [**M+H-dihydrocaffeoyl**]+, 293.19 [**M+H-C2H7N-dihydrocaffeoyl**]+, 222.11, 165.05, 123.04, 100.11	623147	651767	242328	3520552	751140	4059139	3.964	500.27341	[M-H]-	500.27 [**M-H**]-, 455.22 [**M-H-C2H7N**]-, 378.23 [**M-H-122**]-, 214.20 [**M-H-122-dihydrocaffeoyl**]-, 195.06, 135.04	7811	2166	1390	37891	4658	52416
**53**	N-dihydrocoumaroyl-N'-dihydrocaffeoyl spermidine	C25H35N3O5	4.440	458.26361	[M+H]+	458.26 [**M+H**]+, 441.24 [**M+H-NH3**]+, 310.21 [**M+H-dihydrocoumaroyl**]+, 293.19 [**M+H-NH3-dihydrocoumaroyl**]+, 277.19 [**M+H-NH3-**	16093877	17183056	20317594	12917064	26157580	14046091	4.061	456.24817	[M-H]-	456.25 [**M-H**]-, 334.41 [**M-H-122**]-, 308.20 [**M-H-dihydrocoumaroyl**]-, 186.16 [**M-H-122-dihydrocoumaroyl**]-, 137.06,	898352	1014224	1017036	1109826	3221260	995207

						**dihydrocaffeoyl**]+, 236.13, 222.11, 206.12, 165.05, 123.04, 107.05, 100.07, 72.08										121.03, 58.03						
**54**	N-N'-bis-(dihydrocaffeoyl) spermidine-conjugate (+C5H2)	C30H37N3O6	4.468	536.27423	[M+H]+	536.27 [**M+H**]+, 457.23 [**M+H-C5H5N**]+, 293.19 [**M+H-C5H5N-dihydrocaffeoyl**]+, 236.13, 222.11, 165,05, 72.08	2467812	2152761	2212920	4522878	4705013	4011294										
**55**	N-caffeoyl-N'-dihydrocaffeoyl spermidine isomer 2	C25H33N3O6	4.519	472.24384	[M+H]+	472.25 [**M+H**]+, 455.22 [**M+H-NH3**]+, 310.21 [**M+H-caffeoyl**]+, 293.19 [**M+H-NH3-caffeoyl**]+,	731814	852695	1032877	883189	2477392	1032905	4.167	470.22949	[M-H]-	470.22 [**M-H**]-, 350.21 [**M-H-120**]-, 308.20 [**M-H-caffeoyl**]-, 186.16 [**M-H-caffeoyl-122**]-,	32308	60439	18004	84804	356884	92463

						239.14, 222.11, 220.10, 163.04, 123.04, 72.08										161.02, 58.03						
**56**	N-N'-bis-(dihydrocaffeoyl) spermidine-conjugate (+C4H6)	C29H41N3O6	4.538	528.30560	[M+H]+	528.31 [**M+H**]+, 457.23 [**M+H-C4H9N**]+, 293.19 [**M+H-C4H9N-dihydrocaffeoyl**]+, 222.11, 126.13	21950	28759	10909	1502929	283047	515908										
**57**	N-N'-bis-(dihydrocaffeoyl) spermidine-conjugate (+C5H2O) isomer 1	C30H37N3O7	4.634	552.27325	[M+H]+	552.27 [**M+H**]+, 457.23 [**M+H-C5H5NO**]+, 293.18 [**M+H-C5H5NO-dihydrocaffeoyl**]+, 236.12, 222.11, 165.05, 123.04, 112.11	33058149	32452875	32943042	24444075	9156470	34298097	4.313	550.25287	[M-H]-	550.25 [**M-H**]-, 455.22 [**M-H-C5H5NO**]-, 333.18 [**M-H-C5H5NO-122**]-, 291.17 [**M-H-C5H5NO-dihydrocaffeoyl**]-, 279.14, 94.03	920226	1246058	818414	1368890	130470	1974136

**58**	N-dihydroferuloyl-N'-dihydrocaffeoyl spermidine	C26H37N3O6	4.637	488.27502	[M+H]+	488.28 [**M+H**]+, 471.25 [**M+H-NH3**]+, 310.21 [**M+H-dihydroferuloyl**]+, 292.20 [**M+H-dihydroferuloyl-NH4**]+, 250.14, 236.13, 222.11, 179.07, 165.05, 137.06, 123.04, 112.11, 100.07, 72.08	1508244	1636169	1979949	1051719	5585811	917218	4.253	486.26071	[M-H]-	486.26 [**M-H**]-, 468.17 [**M-H-H2O**]-, 441.29, 308.21 [**M-H-dihydroferuloyl**]-, 284.36, 135.04	60141	107070	107302	100786	235423	93442
**59**	N-N'-bis-(dihydrocaffeoyl) spermidine-conjugate (+C4H5N)	C29H40N4O6	4.679	541.30157	[M+H]+	541.30 [**M+H**]+, 457.23 [**M+H-C4H8N2**]+, 377.25 [**M+H-dihydrocaffeoyl**]+, 293.19 [**M+H-**	4927615	4345962	4195855	7006413	8910331	7390992										

						**C4H8N2-dihydrocaffeoyl**]+, 236.13, 222.11, 165.05, 123.04, 112.11																		
**60**	N-N'-bis-(dihydrocaffeoyl) spermidine-conjugate (+C4H3N)	C29H38N4O6	4.763	539.28522	[M+H]+	539.28 [**M+H**]+, 457.23 [**M+H-C4H6N2**]+, 375.24 [**M+H-dihydrocaffeoyl**]+, 293.19 [**M+H-C4H6N2-dihydrocaffeoyl**]+, 236.13, 222.11, 165.05, 123.04, 112.11	1837799	1787123	1559406	1828513	815700	1319551	4.374	537.27124	[M-H]-	537.27 [**M-H**]-, 373.22 [**M-H-dihydrocaffeoyl**]-	24938	26460	15184	17315	6234	20440
**61**	N-N'-bis-(dihydrocaffeoyl) spermidine-conjugate (+C4H6O2)	C29H41N3O8	4.834	560.29669	[M+H]+	560.29 [**M+H**]+, 457.23 [**M+H-C4H9NO2**]+, 396.25 [**M+H-dihydrocaffeoyl**]+, 378.24	1666879	1560483	1609421	3519999	5580890	2978043	4.425	558.28082	[M-H]-	558.28 [**M-H**]-, 436.24 [**M-H-122**]-, 394.23 [**M-H-dihydrocaffeoyl**]-,	7558	14779	14826	185478	159345	129080

						[**M+H-dihydrocaffeoyl-H2O**]+, 293.19 [**M+H-C4H9NO2-dihydrocaffeoyl**]+, 236.13, 222.11, 165.05, 158.12, 130.09, 84.08							362.20, 320.19, 121.02, 58.02
**62**	N-N'-bis-(dihydrocaffeoyl) spermidine-conjugate (+C6H5N) isomer 1	C31H40N4O6	4.831	565.30200	[M+H]+	565.30 [**M+H**]+, 457.23 [**M+H-C6H8N2**]+, 293.19 [**M+H-C6H8N2-dihydrocaffeoyl**]+, 236.13, 222.11, 165.05, 123.04, 112.11	1189782	1245561	1300458	3109323	2373886	1199376	
**63**	N-N'-bis-(dihydrocaffeoyl) spermidine-	C31H44N4O7	4.888	585.32916	[M+H]+	585.33 [**M+H**]+, 541.30 [**M+H-C2H4O**]+, 457.24 [**M+H-**	1053627	763137	787317	1020426	1185516	1096221	

	conjugate (+C6H9NO)					**C6H12N2O**]+, 293.19 [**M+H-C6H12N2O-dihydrocaffeoyl**]+, 222.11																		
**64**	N-N'-bis-(dihydrocaffeoyl) spermidine-conjugate (+C5H5N)	C30H40N4O6	4.941	553.30341	[M+H]+	553.30 [**M+H**]+, 457.23 [**M+H-C5H8N2**]+, 293.19 [**M+H-C5H8N2-dihydrocaffeoyl**]+, 236.13, 222.11, 165.05, 123.04, 112.11	4836659	3671895	4597118	4823861	5525389	3780459	4.560	551.28137	[M-H]-	551.28 [**M-H**]-, 429.25 [**M-H-122**]-, 387.23 [**M-H-dihydrocaffeoyl**]-, 121.02, 95.06	66844	81779	22474	107829	105502	37278
**65**	N-feruroyl-N'-dihydrocaffeoyl spermidine	C26H35N3O6	4.954	486.26096	[M+H]+	486.26 [**M+H**]+, 469.23 [**M+H-NH3**]+, 310.21 [**M+H-feruloyl**]+, 293.19 [**M+H-NH3-feruloyl**]+, 239.14, 222.11,	4915678	5192126	5690980	3878779	10268176	4078686	4.563	484.23907	[M-H]-	484.24 [**M-H**]-, 308.20 [**M-H-feruloyl**]-, 175.04	186121	159832	180723	220012	466668	138621

						177.06, 165.05, 145.03, 123.04, 117.03, 72.08																
**66**	N-dihydrocoumaroyl-N'-dihydrocaffeoyl spermidine conjugate (+C5H2O)	C30H37N3O6	5.065	536.27527	[M+H]+	536.27 [**M+H**]+, 441.24 [**M+H-C5H5NO**]+, 293.18 [**M+H-C5H5NO-dihydrocoumaroyl**]+, 277.19 [**M+H-C5H5NO-dihydrocaffeoyl**]+, 222.11, 220.13, 206.12, 165.05, 123.04, 112.11, 107.05, 100.07	6088769	6233424	5789682	2058679	513238	4995459	4.719	534.25928	[M-H]-	534.26 [**M-H**]-, 439.22 [**M-H-C5H5NO**]-, 386.20 [**M-H-dihydrocoumaroyl**]-, 333.17, 307.18, 291.17 [**M-H-C5H5NO-dihydrocoumaroyl**]-, 254.87, 134.89, 94.03	66510	92362	49989	50044	4582	47030
**67**	N-N'-bis-(dihydrocaffeoyl) spermidine-	C30H38N4O6	5.078	551.28613	[M+H]+	551.29 [**M+H**]+, 457.23 [**M+H-C5H6N2**]+,	122717	8882	11140	3177669	2313962	2601239	4.749	549.27106	[M-H]-	549.27 [**M-H**]-, 427.23 [**M-H-122**]-, 385.22 [**M-**	540	10038	2083	123004	108497	64405

	conjugate (+C5H3N)					387.24 [**M+H-dihydrocaffeoyl**]+, 369.23 [**M+H-dihydrocaffeoyl-H2O**]+, 293.19 [**M+H-C5H6N2-dihydrocaffeoyl**]+, 222.11, 165.05, 149.11, 123.04, 112.11										**H-dihydrocaffeoyl**]-, 121.03						
**68**	N-N'-bis-(dihydrocaffeoyl) spermidine-conjugate (+C7H7N)	C32H42N4O6	5.136	579.32111	[M+H]+	579.32 [**M+H**]+, 457.23 [**M+H-C7H10N2**]+, 293.18 [**M+H-C7H10N2-dihydrocaffeoyl**]+, 236.13, 222.11, 165.05, 123.04, 112.11	20523537	16086352	16753619	13210418	9488666	11653511	4.738	577.29773	[M-H]-	577.30 [**M-H**]-, 455.22 [**M-H-122**]-, 413.25 [**M-H-dihydrocaffeoyl**]-, 308.20, 253.20, 121.03	55021	104682	56134	43051	11064	46661
**69**	N-N'-bis-(dihydrocaffeoyl)	C30H43N3O8	5.190	574.30353	[M+H]+	574.30 [**M+H**]+, 500.26 [**M+H-**	4677123	4549235	4484879	9392726	8643320	7092403	4.796	572.29468	[M-H]-	572.30 [**M-H**]-, 498.26 [**M-H-**	140299	207136	133143	523815	341370	311403

	spermidine-conjugate (+C5H8O2)					**C3H6O2**]+, 457.22 [**M+H-C5H11NO2**]+, 410.25 [**M+H-dihydrocaffeoyl**]+, 392.24 [**M+H-dihydrocaffeoyl-H2O**]+, 336.21 [**M+H-C3H6O2-dihydrocaffeoyl**]+, 293.17 [**M+H-C5H11NO2-dihydrocaffeoyl**]+, 222.11, 172.12, 98.08										**C3H6O2**]-, 450.26 [**M-H-122**]-, 408.25 [**M-H-dihydrocaffeoyl**]-, 376.23 [**M-H-C3H6O2-122**]-, 334.22 [**M-H-C3H6O2-dihydrocaffeoyl**]-, 135.05, 121.03						
**70**	N-N'-bis-(dihydrocaffeoyl) spermidine-conjugate (+C6H7N)	C31H42N4O6	5.344	567.31769	[M+H]+	567.32 [**M+H**]+, 457.23 [**M+H-C6H10N2**]+, 293.19 [**M+H-C6H10N2-dihydrocaffeoyl**]+, 236.13, 222.11,	762485	654957	820331	2194086	3881258	1282542	4.951	565.30200	[M-H]-	565.30 [**M-H**]-, 443.26 [**M-H-122**]-, 423.06, 401.25 [**M-H-dihydrocaffeoyl**]-,	3145	3478	3835	25994	38462	7839

						1650.5, 123.04, 112.11										121.03, 109.08								
**71**	N-N'-bis-(dihydrocaffeoyl) spermidine-conjugate (+C6H5N) isomer 2	C31H40N4O6	5.351	565.30212	[M+H]+	565.30 [**M+H**]+, 457.23 [**M+H-C6H8N2**]+, 401.25 [**M+H-dihydrocaffeoyl**]+, 383.24 [**M+H-dihydrocaffeoyl-H2O**]+, 293.19 [**M+H-C6H8N2-dihydrocaffeoyl**]+, 236.13, 222.11, 123.04	747219	672220	564623	1762987	1318924	1417012	4.984	563.28424	[M-H]-		5935	4433	5149	46119	11924	12399		
**72**	N-N'-bis-(dihydrocaffeoyl) spermidine-conjugate (+CO)	C26H35N3O7	5.388	502.25519	[M+H]+	502.25 [**M+H**]+, 474.26 [**M+H-CO**]+, 338.21 [**M+H-dihydrocaffeoyl**]+, 320.20 [**M+H-dihydrocaffeoyl-H2O**]+,	1575217	1627564	1512678	1343326	2156805	2583337	5.339	500.23798	[M-H]-	500.24 [**M-H**]-, 378.20 [**M-H-122**]-, 336.19 [**M-H-dihydrocaffeoyl**]-, 214.15 [**M-H-122-dihydrocaff**	483696	548974	391409	1567636	858250	1316570		

						222.11, 165.05, 157.14, 123.04, 100.07, 72.08							**eoyl**]-, 186.16, 121.03, 115.09
**73**	N-N'-bis-(dihydrocaffeoyl) spermidine-conjugate (+C8H7N)	C33H42N4O6	5.395	591.31757	[M+H]+	591.32 [**M+H**]+, 457.23 [**M+H-C8H10N2**]+, 293.19 [**M+H-C8H10N2-dihydrocaffeoyl**]+, 236.13, 222.11, 165.05, 123.04	2572075	2162979	2116573	792934	1249662	2032375	
**74**	N-N'-bis-(dihydrocaffeoyl) spermidine-conjugate (+C8H9N) isomer 1	C33H44N4O6	5.543	593.33429	[M+H]+	593.33 [**M+H**]+, 457.23 [**M+H-C8H12N2**]+, 293.19 [**M+H-C8H12N2-dihydrocaffeoyl**]+, 236.13, 222.11, 165.05, 123.04, 112.11	2745762	2423423	2353024	7486439	5842468	4685784	

**75**	N-N'-bis-(dihydrocaffeoyl) spermidine-conjugate (+C2H2O)	C27H37N3O7	5.563	516.26849	[M+H]+	516.27 [**M+H**]+, 457.23 [**M+H-C2H5NO**]+, 352.22 [**M+H-dihydrocaffeoyl**]+, 334.21 [**M+H-dihydrocaffeoyl-H2O**]+, 293.19 [**M+H-C2H5NO-dihydrocaffeoyl**]+, 222.11, 171.15, 165.05, 123.04, 114.09, 100.07	6835322	6959581	6878591	3708138	4616669	4237937	5.511	514.25507	[M-H]-	514.26 [**M-H**]-, 472.25 [**M-H-C2H2O**], 392.22 [**M-H-122**]-, 350.21 [**M-H-dihydrocaffeoyl**]-, 228.17 [**M-H-122-dihydrocaffeoyl**]-, 121.03	1815931	2023693	1460453	1552007	1225567	1487164
**76**	N-N'-bis-(dihydrocaffeoyl) spermidine-conjugate (+C4H2O3)	C29H37N3O9	5.747	572.26086	[M+H]+	572.26 [**M+H**]+, 408.21 [**M+H-dihydrocaffeoyl**]+, 390.20 [**M+H-dihydrocaffeoyl-H2O**]+, 227.14,	1271795	1197199	780446	4933818	1152121	4358162	5.697	570.24347	[M-H]-	570.24 [**M-H**]-, 552.23 [**M-H-H2O**]-, 430.20 [**M-H-H2O-122**]-, 388.19 [**M-H-H2O-**	506724	566490	352522	3367120	519363	2349849

						222.11, 213.12, 165.06, 123.04										**dihydrocaffeoyl**]-, 279.13, 135.04, 121.03						
**77**	N-N'-bis-(dihydrocaffeoyl) spermidine-conjugate (+C8H9N) isomer 2	C33H44N4O6	5.756	593.33380	[M+H]+	593.33 [**M+H**]+, 457.23 [**M+H-C8H12N2**]+, 293.19 [**M+H-C8H12N2-dihydrocaffeoyl**]+, 236.13, 222.11, 165.06, 137.11, 123.04, 112.11	816861	672083	255571	2306634	527038	1282529										
**78**	N-N'-bis-(dihydrocaffeoyl) spermidine-conjugate (+C6H4O5)	C31H39N3O11	5.824	630.26556	[M+H]+	630.27 [**M+H**]+, 466.22 [**M+H-dihydrocaffeoyl**]+, 448.21 [**M+H-dihydrocaffeoyl-H2O**]+, 285.14 [**M+H-dihydrocaffeo dihydrocaffeo**	747936	613390	467242	3824028	1920165	2819037	5.783	628.24738	[M-H]-	628.25 [**M-H**]-, 610.24 [**M-H-H2O**]-, 566.25 [**M-H-62**]-, 472.25 [**M-H-C6H4O5**], 456.21,	580810	774677	396543	4586986	1881164	3172977

						**yl-dihydrocaffeoyl-NH3**]+, 271.13, 222.11, 165.05, 123.04										444.21[**M-H-62-122**]-, 402.20 [**M-H-62-dihydrocaffeoyl**]-, 322.18 [**M-H-62-122-122**]-, 280.17 [**M-H-62-122-dihydrocaffeoyl**], 163.04, 121.03						
**79**	N-dihydrocoumaroyl-N'-dihydrocaffeoyl spermidine-conjugate (+C2H2O)	C27H37N3O6	6.058	500.27502	[M+H]+	500.27 [**M+H**]+, 352.22 [**M+H-dihydrocoumaroyl**]+, 334.21 [**M+H-dihydrocoumaroyl-H2O**]+, 318.22 [**M+H-dihydrocoumaroyl-H2O2**]+, 293.18,	1462573	1516464	1351552	414085	491667	506333	6.010	498.26086	[M-H]-	498.26 [**M-H**]-, 376.22 [**M-H-122**]-, 350.21 [**M-H-dihydrocoumaroyl**]-, 228.17 [**M-H-122-dihydrocoumaroyl**]-,	128340	129794	96581	63328	51807	61343

						222.11, 206.11, 165.05, 123.04										157.10, 121.03						
**80**	N-N'-bis-(dihydrocaffeoyl) spermidine-conjugate (+C8H6O3)	C33H41N3O9	6.160	624.29431	[M+H]+	624.29 [**M+H**]+, 579.32 [**M+H-C2H5O**]+, 457.23 [**M+H-C8H9NO3**]+, 293.19 [**M+H-C8H9NO3-dihydrocaffeoyl**]+, 236.13, 222.11, 165.05, 123.04, 112.11	51163	34489	57119	508995	121004	439669										
**81**	N-N'-bis-(dihydrocaffeoyl) spermidine-conjugate (+C4H2O2)	C29H37N3O8	6.230	556.26532	[M+H]+	556.26 [**M+H**]+, 392.22 [**M+H-dihydrocaffeoyl**]+, 374.21 [**M+H-dihydrocaffeoyl-H2O**]+, 222.11, 211.15, 206.12,	577081	564741	448832	1308490	290794	1184771	6.179	554.25006	[M-H]-	554.25 [**M-H**]-, 536.24 [**M-H-H2O**]-, 432.21 [**M-H-122**]-, 390.20 [**M-H-dihydrocaffeoyl**]-, 268.38 [**M-**	178214	195918	120070	516415	105072	421122

						197.13, 165.05, 154.09, 123.04										**H-122-dihydrocaffeoyl**]-, 163.04, 124.75, 98.05						
**82**	N-N'-bis-(dihydrocaffeoyl) spermidine-conjugate (+C3H2O2)	C28H37N3O8	6.233	544.26624	[M+H]+	544.26 [**M+H**]+, 380.22 [**M+H-dihydrocaffeoyl**]+, 362.21 [**M+H-dihydrocaffeoyl-H2O**]+, 222.11, 199.14, 185.13, 123.04	669164	576949	226231	420269	325429	799669	6.188	542.24951	[M-H]-	542.25 [**M-H**]-, 420.21 [**M+H-122**]+, 378.20 [**M+H-dihydrocaffeoyl**]+, 279.14, 256.16 [**M+H-122-dihydrocaffeoyl**]+, 167.12, 129.10, 121.03	140758	226740	116886	225988	101542	299469
**83**	N-N'-bis-(dihydrocaffeoyl) spermidine-	C31H39N3O8	6.944	582.28229	[M+H]+	582.29 [**M+H**]+, 564.27 [**M+H-H2O**]+, 536.28 [**M+H-**	2431763	2401755	2585092	3346932	2770973	3672842	6.896	580.26270	[M-H]-	580.26 [**M-H**]-, 550.26 [**M-H-30**]-, 458.23 [**M-H-122**]-,	911159	1011736	881257	2447886	1318666	2159128

conjugate (+C6H4O2)				**CH2O2**]+, 400.22 [**M+H-H2O-dihydrocaffeoyl**]+, 382.21 [**M+H-H2O-dihydrocaffeoyl-H2O**]+, 222.11, 219.15, 162.09, 134.10, 123.04										428.22 [**M-H-30-122**]-, 416.22 [**M-H-dihydrocaffeoyl**]-, 386.21 [**M-H-30-dihydrocaffeoyl**]-, 124.03, 94.03						
	6.943	604.26300	[M+Na]+	604.26 [**M+Na**]+	907705	869736	1178325	1013437	1050863	1261939	6.897	616.23987	[M+Cl]-	580.26 [**M-H**]-, 416.22 [**M-H-dihydrocaffeoyl**]-	107137	125844	108022	250874	151213	230961
	6.945	564.27081	[M+H-H2O]+	564.27 [**M+H-H2O**]+, 546.26 [**M+H-H2O-H2O**]+, 536.28 [**M+H-CH2O2**]+, 400.22 [**M+H-H2O-dihydrocaffeoyl**]+, 382.22	588594	590243	635962	956986	659198	943461	6.898	643.26221	[M-H+HNO3]-	580.27 [**M-H**]-, 550.26 [**M-H-30**]-, 416.22 [**M-H-dihydrocaffeoyl**]-	351773	389616	349118	732630	448512	647050

						[**M+H-H2O-dihydrocaffeoyl-H2O**]+, 222.11, 162.09, 134.10																
			6.943	620.23828	[M+K]+	620.24 [**M+K**]+	186727	178355	204348	216042	225335	268871										
**84**	N-N'-bis-(dihydrocaffeoyl) spermidine-conjugate (+C8H8O5)	C33H43N3O11	7.217	658.29688	[M+H]+	658.30 [**M+H**]+, 494.25 [**M+H-dihydrocaffeoyl**]+, 476.24 [**M+H-dihydrocaffeoyl-H2O**]-, 313.18, 299.16, 222.11, 165.05, 123.04	59090	85198	25819	2680935	773268	1376557	7.169	656.27997	[M-H]-	656.28+ [**M-H**]-, 638.27 [**M-H-H2O**]-, 592.23 [**M-H-64**]-, 568.23 [**M-H-88**]-, 470.19 [**M-H-64-122**]-, 428.17 [**M-H-64-dihydrocaffeoyl**]-, 402.20	26134	7923	4705	1349122	265693	526236
			7.212	680.27924	[M+Na]+	680.28 [**M+Na**]+	4165	16708	4734	978626	372088	570602	7.169	692.25745	[M+Cl]-	656.28 [**M-H**]-, 638.27 [**M-H-H2O**]-,	2463	1050	1014	197544	37911	93730

			7.219	696.25415	[M+K]+	696.25 [**M+K**]+	694	1720	1329	192742	76191	116388	7.169	719.27820	[M-H+HNO3]-	592.23 [**M-H-64**]-, 402.20 656.28 [**M-H**]-, 638.27 [**M-H-H2O**]-, 592.23 [**M-H-64**]-, 402.20	5521	4426	749	507438	130829	256045
**85**	N-N'-bis-(dihydrocaffeoyl) spermidine-conjugate (+C6H5N) isomer 3	C31H40N4O6	7.359	565.30371	[M+H]+	565.30 [**M+H**]+, 548.28 [**M+H-NH3**]+, 401.25 [**M+H-dihydrocaffeoyl**]+, 383.24 [**M+H-dihydrocaffeoyl-H2O**]+, 222.11, 163.15, 134.09, 123.04	74645	81394	32070	314627	102601	221023	7.423	563.28552	[M-H]-	563.28 [**M-H**]-, 441.25 [**M-H-122**]-	4008	3813	3211	15112	6689	22681
**86**	N-N'-bis-(dihydrocaffeoyl) spermidine-	C30H37N3O8	7.603	568.26349	[M+H]+	568.27 [**M+H**]+, 404.22 [**M+H-dihydrocaffeo dihydrocaffeo**	419697	470919	319114	3426882	1190683	2742594	7.563	566.24799	[M-H]-	566.25 [**M-H**]-, 444.21 [**M-H-122**]-, 402.21 [**M-**	133210	121418	78234	1515507	325068	906257

conjugate (+C5H2O2)				**yl**]+, 386.21 [**M+H-dihydrocaffeoyl-H2O**]+, 223,.14, 222.11, 209.13, 165.05, 123.04										**H-dihydrocaffeoyl**]-, 322.18 [**M-H-122-122**]-, 280.16 [**M-H-122-dihydrocaffeoyl**]-, 135.04, 121.03						
	7.611	590.24493	[M+Na]+	590.25 [**M+Na**]+	165243	184563	129420	905692	450065	770007	7.563	602.22510	[M+Cl]-	566.25 [**M-H**]-, 444.21 [**M-H-122**]-, 402.21 [**M-H-dihydrocaffeoyl**]-, 322.18 [**M-H-122-122**]-, 121.03	9168	5656	3008	47715	26908	73063
	7.615	606.22345	[M+K]+	606.22 [**M+K**]+	12950	37124	4349	189282	81392	168735	7.560	629.24579	[M-H+HN	566.25 [**M-H**]-, 444.21 [**M-H-122**]-, 402.20 [**M-**	37372	66604	42883	479591	161001	354644

															O3]-	**H-dihydrocaffeoyl**]-						
**87**	N-N'-bis-(dihydrocaffeoyl) spermidine-conjugate (+C5H2O) isomer 2	C30H37N3O7	7.929	552.27155	[M+H]+	552.27 [**M+H**]+, 524.28 [**M+H-CO**]+, 388.22 [**M+H-dihydrocaffeoyl**]+, 370.21 [**M+H-dihydrocaffeoyl-H2O**]-, 360.23 [**M+H-CO-dihydrocaffeoyl**]+, 342.22 [**M+H-CO-dihydrocaffeoyl-H2O**]+, 222.11, 207.15, 165.05, 123.04	6129684	6304636	6214365	7204356	2450469	9286402	7.885	550.25378	[M-H]-	550.25 [**M-H**]-, 428.22 [**M-H-122**]-, 386.21 [**M-H-dihydrocaffeoyl**]-, 333.18, 157.10, 121.03, 94.03	1517219	1591384	1352586	2811808	559718	3205740
			7.930	574.25171	[M+Na]+	574.25 [**M+Na**]+	1787705	1820296	1992567	1752953	727796	2370048	7.887	586.23016	[M+Cl]-	550.25 [**M-H**]-, 428.22 [**M-H-122**]-, 386.21 [**M-**	87073	47210	74691	157080	36565	185074

			7.930	590.22827	[M+K]+	590.23 [**M+K**]+	316862	334163	343111	330874	141230	455153	7.885	613.24902	[M-H+HNO3]-	**H-dihydrocaffeoyl**]- 550.25 [**M-H**]-, 428.22 [**M-H-122**]-, 386.21 [**M-H-dihydrocaffeoyl**]-, 333.18, 94.03	511851	508241	460759	859058	233582	1023360
**88**	N-N'-bis-(dihydrocaffeoyl) spermidine-conjugate (+C4H2)	C29H37N3O6	8.247	524.27649	[M+H]+	524.28 [**M+H**]+, 360.23 [**M+H-dihydrocaffeoyl**]+, 342.22 [**M+H-dihydrocaffeoyl-H2O**]+, 222.11, 165.05, 123, 122.10	931261	924013	1110526	2118503	966497	2278672	8.224	522.25897	[M-H]-	522.26 [**M-H**]-, 400.22 [**M-H-122**]-, 358.21 [**M-H-dihydrocaffeoyl**]-, 236.18 [**M-H-122-dihydrocaffeoyl**]-, 121.03, 66.03	437379	425774	456261	1951869	613278	1766210

			8.245	546.26013	[M+Na]+	546.26 [**M+Na**]+	172468	170417	226919	252968	170548	292422	8.223	558.23486	[M+Cl]-		31511	32995	30803	93488	35591	85340
			8.246	562.23535	[M+K]+		40368	34458	54736	73355	43753	81758	8.224	585.25641	[M-H+HNO3]-	522.26 [**M-H**]-, 400.22 [**M-H-122**]-, 358.21 [**M-H-dihydrocaffeoyl**]-, 236.18 [**M-H-122-dihydrocaffeoyl**]-, 163.04, 121.03	176578	181274	199754	489940	196914	465218
**89**	N-N'-bis-(dihydrocaffeoyl) spermidine-conjugate (+C6H4O)	C31H39N3O7	8.257	566.28680	[M+H]+	566.28 [**M+H**]+, 538.29 [**M+H-CO**]+, 524.27 [**M+H-42**]+, 402.24 [**M+H-dihydrocaffeoyl**]+, 384.23 [**M+H-dihydrocaffeoyl-H2O**]+,	1395748	1574753	1607628	1292540	641791	1771296	8.233	564.27063	[M-H]-	564.27 [**M-H**]-, 442.22 [**M-H-122**]-, 424.22 [**M-H-122-H2O**]-, 400.22 [**M-H-dihydrocaffeoyl**]-, 348.81,	394675	360191	368369	492456	210512	585041

						222.11, 165.05, 136.11, 123.04										280.20, 157.10, 117.93						
			8.257	588.27075	[M+Na]+	588.27 [**M+Na**]+	294717	314772	371007	210146	147673	294214	8.236	600.24597	[M+Cl]-		32784	29353	31737	36036	17418	40164
			8.257	604.24597	[M+K]+		60404	66349	78887	45425	28593	60505	8.235	627.26727	[M-H+HNO3]-	564.27 [**M-H**]-, 442.22 [**M-H-122**]-	149767	147974	160479	148258	75904	177725
**90**	N-dihydrocoumaroyl-N'-dihydrocaffeoyl spermidine-conjugate (+C5H2O)	C30H37N3O6	8.278	536.27673	[M+H]+	536.28 [**M+H**]+, 388.22 [**M+H-dihydrocoumaroyl**]+, 370.21 [**M+H-dihydrocoumaroyl-H2O**]+, 354.22 [**M+H-dihydrocoumaroyl-H2O2**]+, 222.11, 206.12, 165.05, 107.05	568662	617394	679334	347513	125529	602221	8.254	534.26086	[M-H]-	534.26 [**M-H**]-, 386.21 [**M-H-dihydrocoumaroyl**]-, 333.80, 264.14 [**M-H-dihydrocoumaroyl-122**]-, 121.03, 94.03	71758	68355	68281	67155	19852	84900

			8.279	558.25879	[M+Na]+	558.26 [**M+Na**]+	136413	148539	175673	73383	38377	116019	8.254	570.23633	[M+Cl]-		18405	8804	17932	10942	3524	13177
**91**	N-N'-bis-(dihydrocaffeoyl) spermidine-conjugate (+C8H8O2)	C33H43N3O8	8.452	610.31274	[M+H]+	610.29 [**M+H**]+, 564.27 [**M+H-46**]+, 536.27 [**M+H-74**]+, 400.22 [**M+H-46-dihydrocaffeoyl**]+, 382.21 [**M+H-46-dihydrocaffeoyl-H2O**]+, 222.11, 162.09, 134.10	218021	287287	194199	581056	67628	599814	8.437	608.29578	[M-H]-	608.30 [**M-H**]-, 486.26 [**M-H-122**]-, 444.25 [**M-H-dihydrocaffeoyl**]-, 122.02	105831	94074	71122	271473	27886	242147
**92**	Hederagenin-tetraglucoside	C53H86O22	8.847	1075.56775	[M+H]+	1075.55 [**M+H**]+, 455.35 [**M+H-C6H10O5-C6H10O5-C6H10O4-C5H8O4-H2O**]+, 309.11, 279.11	113194	125668	143942	95924	112689	104260	8.835	1073.55347	[M-H]-	1073.55 [**M-H**]-, 911.50 [**M-H-C6H10O5**]-, 765.44 [**M-H-C6H10O5-C6H10O4**]-, 749.45 [**M-**	1884968	1759630	2083888	1612647	1740375	1604058

													**H-C6H10O5-C6H10O5**]-, 603.39 [**M-H-C6H10O5-C6H10O5-C6H10O4**]-, 585.37 [**M-H-C6H10O5-C6H10O5-C6H10O4-H2O**]-, 471.35 [**M-H-C6H10O5-C6H10O5-C6H10O4-C5H8O4**]-						
8.865	1097.55151	[M+Na]+	1097.55 [**M+Na**]+	170462	173768	193264	128957	163609	149822	8.835	1109.53284	[M+Cl]-	1109.53 [**M+Cl**]-, 1073.55 [**M-H**]-	448036	436518	519788	356744	413383	370140
8.853	913.51672	[M+H-C6H1	913.50 [**M+H-C6H10O5**]+, 751.47 [**M+H-**	257862	283121	298902	192389	228184	204402	8.834	1136.54944	[M-H+HN	1136.55 [**M-H+HNO3**]-,	546390	526631	727042	357434	477111	377209

		0O5]+	**C6H10O5-C6H10O5**], 455.35 [**M+H-C6H10O5-C6H10O5-C6H10O4-C5H8O4-H2O**]+, 437.34 [**M+H-C6H10O5-C6H10O5-C6H10O4-C5H8O4-H2O-H2O**]+, 309.12, 279.11									O3]-	1073.55 [**M-H**]-, 911.50 [**M-H-C6H10O5**]-, 870.40, 806.39, 765.44 [**M-H-C6H10O5-C6H10O4**]-						
8.849	751.46271	[M+H-C12H20O10]+	751.46 [**M+H-C6H10O5-C6H10O5**]+, 455.35 [**M+H-C6H10O5-C6H10O5-C6H10O4-C5H8O4-H2O**]+, 437.34 [**M+H-C6H10O5-C6H10O5-**	216815	269614	312747	198500	245903	210635	8.835	1191.49463	[M-H+118]-	1191.49 [**M-H+118**]-, 1174.47, 1073.55 [**M-H**]-	455414	425867	500287	416875	474699	396211

			8.845	455.35321	[M+H-C33H40O19]+	**C6H10O4-C5H8O4-H2O-H2O**]+, 279.11 455.35 [**M+H-C6H10O5-C6H10O5-C6H10O4-C5H8O4-H2O**]+, 437.34 [**M+H-C6H10O5-C6H10O5-C6H10O4-C5H8O4-H2O-H2O**]+, 409.34	337826	378155	434528	278109	349459	296182										
			8.847	437.34244	[M+H-C33H42O20]+	437.34 [**M+H-C6H10O5-C6H10O5-C6H10O4-C5H8O4-H2O-H2O**]+, 391.34, 215.18	294359	336867	387444	246018	302383	265939										
**93**	Hederagenin-triglucoside	C47H76O17	8.948	913.51947	[M+H]+	913.50 [**M+H**]+, 751.46 [**M+H-C6H10O5**]+, 619.42 [**M+H-**	335366	363973	428999	281073	338189	289419	8.935	911.50269	[M-H]-	911.50 [**M-H**]-, 749.44 [**M-H-C6H10O5**]-, 603.39 [**M-**	636814	574177	635054	535092	563789	518642

			**C6H10O5-C5H8O4**]+, 455.35 [**M+H-C6H10O5-C6H10O4-C5H8O4-H2O**]+, 437.34 [**M+H-C6H10O5-C6H10O4-C5H8O4-H2O-H2O**]+, 309.12, 279.11										**H-C6H10O5-C6H10O4**]-, 471.35 [**M-H-C6H10O5-C6H10O4-C5H8O4**]-						
8.965	935.49963	[M+Na]+	935.50 [**M+Na**]+	359316	383199	451234	243333	358409	303273	8.936	947.47931	[M+Cl]-	947.48 [**M+Cl**]-, 911.50 [**M-H**]-	410252	395332	471948	356391	396357	361015
8.950	751.46436	[M+H-C6H10O5]+	751.46 [**M+H-C6H10O5**]+, 455.35 [**M+H-C6H10O5-C6H10O4-C5H8O4-H2O**]+, 437.34 [**M+H-C6H10O5-C6H10O4-**	671696	743290	884280	538614	675127	590384	8.937	957.50531	[M+FA-H]-	957.49 [**M+FA-H**]-, 911.50 [**M-H**]-, 749.45 [**M-H-C6H10O5**]-	1561694	1476031	1775353	1457883	1622730	1425804

			**C5H8O4-H2O-H2O**]+, 279.11																
8.952	619.42194	[M+H-C11H18O9]+		87627	94838	78847	68439	81181	73330	8.935	974.49597	[M-H+HNO3]-	974.49 [**M-H+HNO3**]-	1153838	1114537	1498428	945098	1165588	979426
8.951	601.41229	[M+H-C11H20O10]+	601.40 [**M+H-C6H10O5-C5H8O4-H2O**]+, 455.35 [**M+H-C6H10O5-C6H10O4-C5H8O4-H2O**]-, 437.34 [**M+H-C6H10O5-C6H10O4-C5H8O4-H2O-H2O**]	129769	144462	79026	107676	126805	117425	8.935	1029.43445	[M-H+118]-	1029.44 [**M-H+118**]-, 911.51 [**M-H**]-	923794	840359	1014165	943122	1058665	867558
8.948	455.35168	[M+H-C17H30O14]+	455.35 [**M+H-C6H10O5-C6H10O4-C5H8O4-H2O**]+, 437.34 [**M+H-**	849147	961922	1122942	687127	877333	758295										

			8.948	437.34216	[M+H-C17H32O15]+	**C6H10O5-C6H10O4-C5H8O4-H2O-H2O**]+, 409.35 437.34 [**M+H-C6H10O5-C6H10O4-C5H8O4-H2O-H2O**]+, 391.34, 203.18	712827	820881	958012	586134	770718	636016										
**94**	Hederagenin-diglucoside	C41H66O13	9.078	767.45996	[M+H]+	767.47 [**M+H**]+, 455.35 [**M+H-C6H10O5-C5H8O4-H2O**]+, 437.34 [**M+H-C6H10O5-C5H8O4-H2O-H2O**]+	154245	167668	192613	43007	1592	79413	9.061	765.44226	[M-H]-	765.44 [**M-H**]-, 603.37 [**M-H-C6H10O5**]-, 471.35 [**M-H-C6H10O5-C5H8O4**]-	132573	121652	135242	45225	966	72971
			9.077	789.43835	[M+Na]+	789.44 [**M+Na**]+	881706	913901	969883	300546	28268	485026	9.062	801.41968	[M+Cl]-		113347	112751	119569	55864	3724	77765
			9.076	605.40704	[M+H-C6H1		33463	35455	42970	11935	533	17127	9.062	811.44574	[M+FA-H]-	811.44 [**M+FA-H**]-, 765.44 [**M-H**]-, 603.39	847562	778840	868883	288524	19133	480752

		0O5]+											[**M-C6H10O5**]-, 471.34 [**M-H-C6H10O5-C5H8O4**]-						
9.077	587.39667	[M+H-C6H12O6]+	587.38 [**M+H-C6H10O5-H2O**]+, 455.35 [**M+H-C6H10O5-C5H8O4-H2O**]+, 437.34 [**M+H-C6H10O5-C5H8O4-H2O-H2O**]+	148429	158374	185988	46844	4403	84330	9.062	828.43970	[M-H+HNO3]-		352272	326043	401948	114860	9566	186026
9.078	455.35263	[M+H-C11H20O10]+	455.35 [**M+H-C6H10O5-C5H8O4-H2O**]+, 437.34 [**M+H-C6H10O5-C5H8O4-H2O-H2O**]+, 409.35	1299863	1492286	1628569	464728	65442	743852	9.060	883.37891	[M-H+118]-	883.38 [**M-H+118**]-, 765.44 [**M-H**]-	357455	322618	360084	161747	8130	226486

			9.084	437.33984	[M+H-C11H22O11]+	437.34 [**M+H-C6H10O5-C5H8O4-H2O-H2O**]+, 391.34, 203.18	1146665	1289198	1404875	263305	218536	656660										
**95**	Oleanolic acid-tetraglucoside	C53H86O21	9.199	1081.55713	[M+Na]+	1081.56 [**M+Na**]+	215682	219728	236766	151571	195310	179074	9.183	1057.55688	[M-H]-	1057.56 [**M-H**]-, 895.51 [**M-H-C6H10O5**]-, 733.45 [**M-H-C6H10O5-C6H10O5**]-, 587.40 [**M-H-C6H10O5-C6H10O5-C6H10O4**]-, 455.35 [**M-H-C6H10O5-C6H10O5-C6H10O4-C5H8O4**]-	1611676	1536721	1673980	1392663	1389177	1390980

			9.200	1076.59875	[M+NH4]+	1076.58 [**M+NH4**]+	126022	140139	149344	96845	120269	109310	9.178	1093.53284	[M+Cl]-	1093.54 [**M+Cl**]-, 1057.56 [**M-H**]-, 895.51 [**M-H-C6H10O5**]-, 733.45 [**M-H-C6H10O5-C6H10O5**]-	459164	464705	456746	423076	449883	417159
													9.179	1120.55090	[M-H+HNO3]-	1120.56 [**M-H+HNO3**]-, 1057.56 [**M-H**]-	703335	688708	741978	585145	628334	604024
													9.179	1175.49622	[M-H+118]-		426819	379660	386386	455765	464107	417635
**96**	alpha-hederin	C41H66O12	9.243	751.46484	[M+H]+	751.45 [**M+H**]+, 455.35 [**M+H-C6H10O4-C5H8O4-H2O**]+, 437.34 [**M+H-C6H10O4-**	1879338	2128350	2466941	1709793	2082227	1882934	9.245	749.44702	[M-H]-	749.45 [**M-H**]-, 603.38 [**M-H-C6H10O4**]-, 585.38 [**M-H-C6H10O4-H2O**]-,	346400	338034	222958	229029	205696	333044

			**C5H8O4-H2O-H2O**]+, 409.35, 279.11										471.35 [**M-H-C6H10O4-C5H8O4**]-						
9.250	773.44818	[M+Na]+	773.45 [**M+Na**]+	272777	295699	352754	231083	282464	267967	9.251	785.42456	[M+Cl]-	785.42 [**M+Cl**]-, 749.45 [**M-H**]-, 471.35 [**M-H-C6H10O4-C5H8O4**]-	305792	407156	433484	297751	326960	310018
9.247	619.42322	[M+H-C5H8O4]+	619.42 [**M+H-C5H8O4**]+, 455.35 [**M+H-C6H10O4-C5H8O4-H2O**]+, 437.34 [**M+H-C6H10O4-C5H8O4-H2O-H2O**]+	224161	239607	289053	195140	234562	218245	9.245	795.45148	[M+FA-H]-	795.45 [**M+FA-H**]-, 749.45 [**M-H**]-, 603.39 [**M-H-C6H10O4**]-, 585.38 [**M-H-C6H10O4-H2O**]-, 471.35 [**M-H-C6H10O4-C5H8O4**]-	2668320	2550296	2801925	2609240	2688183	2540111

9.242	601.40973	[M+H-C5H10O5]+	601.41 [**M+H-C5H8O4-H2O**]+, 437.34 [**M+H-C6H10O4-C5H8O4-H2O-H2O**]+	559757	596664	761651	517015	657971	530852	9.247	812.44098	[M-H+HNO3]-	812.44 [**M-H+HNO3**]-	1870495	1799986	1999535	1587279	1673117	1658024
9.245	455.35260	[M+H-C11H20O9]+	455.35 [**M+H-C6H10O4-C5H8O4-H2O**]+, 437.34 [**M+H-C6H10O4-C5H8O4-H2O-H2O**]+, 409.35	2980442	3361987	3790378	2561191	3170931	2855285	9.248	867.38312	[M-H+118]-		1405907	1254375	1441835	1483438	1481128	1376862
9.243	437.34204	[M+H-C11H22O10]+	437.34 [**M+H-C6H10O4-C5H8O4-H2O-H2O**]+, 391.34, 203.18	2533516	2828326	3223261	2239233	2795120	2438830										
9.247	409.34869	[M+H-C12H22O11]+		338317	373624	441389	283257	354655	320132										

**97**	Oleanolic acid-triglucoside	C47H76O16	9.351	897.51782	[M+H]+		15075	44097	25101	20301	31588	24277	9.336	895.50769	[M-H]-	895.51 [**M-H**]-, 749.45 [**M-H-C6H10O4**]-, 733.46 [**M-H-C6H10O5**]-, 587.39 [**M-H-C6H10O5-C6H10O4**]-, 455.36 [**M-H-C6H10O5-C6H10O4-C5H8O4**]-	205853	173985	268712	188947	185756	149469
			9.341	919.50067	[M+Na]+	919.50 [**M+Na**]+	231830	277325	272146	221850	296007	250369	9.389	931.48492	[M+Cl]-	931.48 [**M+Cl**]-, 895.51 [**M-H**]-, 733.45 [**M-H-C6H10O5**]-, 455.34 [**M-H-C6H10O5-C6H10O4-C5H8O4**]-	495612	445526	475282	674421	709021	658544

			9.359	914.54871	[M+NH4]+	914.55 [**M+NH4**]+, 439.36 [**M+H-C6H10O5-C6H10O4-C5H8O4-H2O**]+, 279.11	892122	2192853	1159181	2641671	2324891	2894213	9.353	941.51050	[M+FA-H]-	941.51 [**M+FA-H**]-, 895.51 [**M-H**]-, 733.45 [**M-H-C6H10O5**]-, 587.41 [**M-H-C6H10O5-C6H10O4**]-, 455.35 [**M-H-C6H10O5-C6H10O4-C5H8O4**]-	3104724	1586626	1861677	2543019	2937999	2426224
			9.360	439.35159	[M+H-C17H30O14]+	439.35 [**M+H-C6H10O5-C6H10O4-C5H8O4-H2O**]+, 203.18, 191.18	1933752	2150995	2347072	1727169	2379941	1997828	9.384	958.50183	[M-H+HNO3]-	958.50 [**M-H+HNO3**]-	1582442	1499828	1745771	1867823	2291349	1807070
													9.385	1013.44208	[M-H+118]-	1013.44 [**M-H+118**]-	885255	797681	854281	1695698	1819672	1222960
**98**	Hederagenin-monoglucoside	C35H56O8	9.393	605.40674	[M+H]+		25507	22809	29619	6903	440	15124	9.396	603.39038	[M-H]-	603.39 [**M-H**]-	308124	309225	289670	100702	1564	157633

9.403	627.38733	[M+Na]+		64405	121953	123560	35393	1708	60178	9.399	639.36450	[M+Cl]-	639.35 [**M+Cl**]-, 603.39 [**M-H**]-	105780	107526	106995	34728	2395	58993
9.394	587.39349	[M+H-H2O]+	587.38 [**M+H-H2O**]+, 437.34 [**M+H-H2O-C5H8O4-H2O**]+	166582	174468	207669	58438	4828	97935	9.391	649.39410	[M+FA-H]-	649.40 [**M+FA-H**]-, 603.39 [**M-H**]-	504712	496487	540432	188909	9768	306224
9.393	569.38190	[M+H-2H2O]+		46493	49409	51093	15686	1335	27994	9.393	666.38336	[M-H+HNO3]-	666.39 [**M-H+HNO3**]-	547834	562766	528041	188283	12617	286946
9.393	455.34970	[M+H-C5H10O5]+	455.35 [**M+H-H2O-C5H8O4**]+, 437.34 [**M+H-H2O-C5H8O4-H2O**]+, 409.35	1482099	1623635	1747762	496655	70737	874270	9.387	1207.78918	[2M-H]-	1207.79 [**2M-H**]-, 603.39 [**M-H**]-	222769	223636	222519	29082	231	75962
9.393	409.34555	[M+H-C6H12O7]+		164719	180397	180376	55796	11597	106565										

**99**	Oleanolic acid-diglucoside	C41H66O12	9.563	773.44318	[M+Na]+	773.44 [**M+Na**]+	2229534	1141274	1055202	1377595	308757	1804257	9.544	749.44763	[M-H]-	749.45 [**M-H**]-, 587.38 [**M-H-C6H10O5**]-	226468	255352	370782	348035	109064	425504
			9.565	768.49200	[M+NH4]+	768.49 [**M+NH4**]+, 439.36 [**M+H-C6H10O5-C5H8O4-H2O**]+	12232098	13406362	14540392	4947224	366553	7677270	9.543	785.42419	[M+Cl]-	785.43 [**M+Cl**]-, 749.45 [**M-H**]-, 587.39 [**M-H-C6H10O5**]-, 455.35 [**M-H-C6H10O5-C5H8O4**]-	344241	341507	377438	247076	50881	276535
			9.569	439.35754	[M+H-C11H20O10]+	439.36 [**M+H-C6H10O5-C5H8O4-H2O**]+, 203.18	2056654	2361282	2619927	726548	331800	1447377	9.544	795.45117	[M+FA-H]-	795.45 [**M+FA-H**]-, 749.45 [**M-H**]-, 587.39 [**M-H-C6H10O5**]-, 455.35 [**M-H-C6H10O5-C5H8O4**]-	2720882	2553061	2791875	1584504	383074	1965191
													9.543	812.44202	[M-H+HN	812.44 [**M-H+HNO3**]-	1882175	1782059	2010240	768792	204173	1219075

													9.539	867.38489	O3] - [M-H+118]-		733617	614872	674423	469300	119943	555091
**100**	1,2-dioleoyl-sn-glycero-3-phosphatidylcholine	C44H84NO8P	9.665	786.60046	[M+H]+	786.60 [**M+H**]+, 184.07 [**M-C39H70O4+H**]+, 86.10 [**M-C39H73O8P+H**]+	108657	134247	198916	170153	166469	135990										
**101**	Beta-hederin	C41H66O11	9.734	752.49518	[M+NH4]+	752.50 [**M+NH4**]+, 439.36 [**M+H-C6H10O4-C5H8O4-H2O**]+, 279.11	419886	106921	443420	288945	183271	440599	9.762	733.45026	[M-H]-	733.45 [**M-H**]-, 689.46, 587.39 [**M-H-C6H10O4**]-, 455.35 [**M-H-C6H10O4-C5H8O4**]-	827032	811144	861977	937187	1519889	820387
			9.731	457.37094	[M+H-C11H18O8]+	457.35 [**M+H-C6H10O4-C5H8O4**]+, 439.36 [**M+H-C6H10O4-C5H8O4-**	46981	131126	77405	39102	81312	122430	9.798	769.43079	[M+Cl]-	769.43 [**M+Cl**]-, 733.45 [**M-H**]-, 455.35 [**M-H-**	342840	453944	472249	200670	391732	224230

						**H2O**]+, 411.36, 203.18										**C6H10O4-C5H8O4**]-						
			9.730	439.35403	[M+H-C11H20O9]+	439.36 [**M+H-C6H10O4-C5H8O4-H2O**]+, 249.19, 203.18	2746361	3293100	1246833	2603060	3118169	1373710	9.754	779.45654	[M+FA-H]-	779.46 [**M+FA-H**]-, 733.45 [**M-H**]-, 689.46, 587.39 [**M-H-C6H10O4**]-, 455.35 [**M-H-C6H10O4-C5H8O4**]-	2225654	2181606	2284448	2583437	3285358	2336386
													9.757	796.44623	[M-H+HNO3]-	796.45 [**M-H+HNO3**]-	2022354	1969526	1905295	1830890	1999808	2013468
													9.763	851.38910	[M-H+118]-		474241	462253	482299	581624	566802	574412
**102**	1-Oleoyl-2-palmitoyl-sn-glycero-3-phosphocholine	C42H82NO8P	9.816	760.58789	[M+H]+	760.58 [**M+H**]+, 184.08 [**M-C37H68O4+H**]+, 125.00 [**M-C40H77NO4+**	133735	209028	296533	327755	343085	135767										

						**H**]+, 86.10 [**M-C37H71O8P+H**]+																
			9.836	473.36429	[M+H]+		80567	83998	92309	5559	11262	19814	9.822	471.34564	[M-H]-	471.35 [**M-H**]-	2621522	2745376	2521143	504387	25972	977996
			9.836	495.34656	[M+Na]+		147907	150473	141155	1644	1395	20495	9.824	943.70270	[2M-H]-	943.70 [**2M-H**]-, 471.35 [**M-H**]-	749691	798996	691906	32043	50	110002
**103**	Hederagenin	C30H48O4	9.831	455.35291	[M+H-H2O]+	455.35 [**M+H-H2O**]+, 437.34 [**M+H-H2O-H2O**]+, 409.35	2167640	2190886	2222524	269765	30124	617383	9.824	965.68677	[2M+Na-2H]-	965.69 [**2M+Na-2H**]-, 805.99, 471.35 [**M-H**]-	205563	226322	189784	20682	83	55561
			9.834	437.34259	[M+H-2H2O]+	437.34 [**M+H-H2O-H2O**]+, 391.34	1814648	1656393	1716036	167745	23125	353639										
			9.835	945.71558	[2M+H]+	945.71 [**2M+H**]+, 473.36 [**M+H**]+, 455.35 [**M+H-H2O**]+, 437.34	297260	297590	288373	2175	144	31839										

			9.836	967.69879	[2M+Na]+	[**M+H-H2O-H2O**]+, 207.18 967.70 [**2M+Na**]+, 495.35 [**M+Na**]+	222313	206989	190859	2603	69	19429										
**104**	1-Palmitoyl-sn-glycero-3-phosphocholine	C24H50NO7P	9.852	496.34091	[M+H]+	496.34 [**M+H**]+, 478.96 [**M+H-H2O**]+, 184.07 [**C19H36O3+H**]+, 104.11 [**C19H37O6P+H**]+	405358	73006	113472	995	2842	335413										
**105**	Oleanolic acid-monoglucoside	C35H56O7	10.002	606.44220	[M+NH4]+	606.43 [**M+NH4**]+, 439.36 [**M+H-C5H8O4-H2O**]+, 393.36, 247.17	64689	118903	60603	28010	591	71566	9.993	587.39233	[M-H]-	587.39 [**M-H**]-	4396015	4186456	4547642	2030496	134213	2766899
			10.002	611.39447	[M+Na]+		33092	18967	12570	21954	1744	28435	9.992	623.37067	[M+Cl]-	587.39 [**M-H**]-	57323	134763	91049	112149	7754	146936
			10.001	439.35693	[M+H-C5H1	439.35 [**M+H-C5H8O4-H2O**]+, 205.19	292057	1229954	661844	437294	42317	966012	9.946	633.39948	[M+FA-H]-		201360	188553	193966	47374	1250	83331

					0O5]+																	
			10.003	1194.83533	[2M+NH4]+		16057	24871	17631	4042	71	6898	9.989	1175.79749	[2M-H]-	1175.80 [**2M-H**]-, 587.39 [**M-H**]-	5828845	5522528	6024044	1265907	5185	2487571
			10.007	1199.79968	[2M+Na]+	1199.79 [**2M+Na**]+, 611.39 [**M+Na**]+	205273	78439	73085	215222	1285	94449	9.993	1197.78455	[2M+Na-2H]-	1197.79 [**2M+Na-2H**]-, 587.40 [**M-H**]-	436747	258427	442060	231752	1439	345879
**106**	Palmitoyl ethanolamide	C18H37NO2	10.581	300.28903	[M+H]+	282.28 [**M+H-H2O**]+, 239.24 [**M-C2H7NO+H**]+, 109.10 [**M-C10H25NO2-H**]+, 95.08 [**M-C11H27NO2+H**]+, 85.10 [**M-C12H25NO2+H**]+, 71.09 [**M-C13H27NO2+H**]+, 62.06 [**M-C16H30O+H**]+, 57.07 [**M-**	420312	484106	640269	212028	99728	298755										

					**C14H29NO2+ H**]+							
**107**	1-Oleoyl-sn-glycero-3-phosphocholine	C26H52NO7P	10.689	522.35638	522.35 [**M+H**]+, 504.34 [**M+H-H2O**]+, 184.07 [**M-C21H38O3+H**]+, 104.11 [**M-C21H39O6P+H**]+	1211476	8645	273420	1099	673	2109709	
**108**	N-Oleoylethanolamine	C20H39NO2	10.700	326.30621	326.31 [**M+H**]+, 308.30 [**M+H-H2O**]+, 135.12 [**M-C10H25NO2-H**]+, 121.10 [**M-C11H27NO2-H**]+, 109.10 [**M-C12H27NO2-H**]+, 95.09 [**M-C13H29NO2-H**]+, 83.08 [**M-C14H29NO2-**	4895680	5333005	6076014	2497780	492165	4025264	

						**H**]+, 69.07 [**M-C15H31NO2-H**]+, 62.06 [**M-C18H32O+H**]+																
**109**	16-Hydroxypalmitic acid	C16H32O3											10.566	271.22910	[M-H]-		111059	114272	137588	42867	22365	74484
			10.702	457.36810	[M+H]+		222809	214720	204580	19944	5305	60183	10.687	455.34976	[M-H]-	455.35 [**M-H**]-	8487607	8293719	8015387	1778127	133248	3441036
			10.702	439.35730	[M+H-H2O]+	439.36 [**M+H-H2O**]+, 249.19, 203.18	1786500	1844413	1597305	168409	50243	411806	10.685	911.71344	[2M-H]-	867.51, 455.35 [**M-H**]-	198937	189436	184645	14721	146	49128
**110**	Oleanolic acid	C30H48O3	10.700	479.35083	[M+Na]+	479.35 [**M+Na**]+	381533	402787	336954	3598	917	26462	10.685	933.69366	[2M+Na-2H]-	933.70 [**2M+Na-2H**]-, 455.35 [**M-H**]-	1131123	1081951	1084945	153328	649	344425
			10.701	935.70819	[2M+Na]+	935.71 [**2M+Na**]+, 479.35 [**M+Na**]	593467	560416	468363	2981	97	27360										
**111**	Triethylene Glycol Bis(2-	C22H42O6	10.834	403.30231	[M+H]+	171.14 [**M+H-C10H16O6**]+, 73.03, 57.07	1148684	1496093	1660674	2258676	2437811	2397342										

	ethylhexanoate)		10.835	425.28665	[M+Na]+	425.29 [**M+Na**]+	2874561	3785995	4256551	5454772	6430159	5640178										
			10.833	420.33414	[M+NH4]+	171.14 [**M+H-C10H16O6**]+, 57.07	19366511	23400948	25315765	26917560	31291395	29321350										
			10.833	171.13455	[M+H-C10H16O6]+	171.14 [**M+H-C10H16O6**]+, 73.03, 57.07	2398409	3025630	3197590	4258554	4777309	4308624										
**112**	Diisodecyl phthalate	C28H46O4	12.202	433.33344	[M+H-CH2]+	149.02, 85.10, 71.08, 57.07	84538	105520	231255	1156054	1125710	1073544										
**113**	Oleic acid	C18H34O2											11.195	281.24533	[M-H]-	281.25 [**M-H**]-	304499	290691	347554	273556	245309	285611
**114**	Brassicasterol 3-monoglucoside	C34H56O6											11.708	605.40302	[M+FA-H]-	605.41 [**M+FA-H**]-, 559.39 [**M-H**]-	186555	182882	132048	12424	374	31104
